# Quantifying intermolecular interactions of ionic liquids using cohesive energy densities

**DOI:** 10.1098/rsos.171223

**Published:** 2017-12-06

**Authors:** Kevin R. J. Lovelock

**Affiliations:** Department of Chemistry, University of Reading, Reading, UK

**Keywords:** ionic liquids, cohesive energy density, intermolecular interactions

## Abstract

For ionic liquids (ILs), both the large number of possible cation + anion combinations and their ionic nature provide a unique challenge for understanding intermolecular interactions. Cohesive energy density, *ced*, is used to quantify the strength of intermolecular interactions for molecular liquids, and is determined using the enthalpy of vaporization. A critical analysis of the experimental challenges and data to obtain *ced* for ILs is provided. For ILs there are two methods to judge the strength of intermolecular interactions, due to the presence of multiple constituents in the vapour phase of ILs. Firstly, *ced*_IP_, where the ionic vapour constituent is neutral ion pairs, the major constituent of the IL vapour. Secondly, *ced*_C+A_, where the ionic vapour constituents are isolated ions. A *ced*_IP_ dataset is presented for 64 ILs. For the first time an experimental *ced*_C+A_, a measure of the strength of the total intermolecular interaction for an IL, is presented. *ced*_C+A_ is significantly larger for ILs than *ced* for most molecular liquids, reflecting the need to break all of the relatively strong electrostatic interactions present in ILs. However, the van der Waals interactions contribute significantly to IL volatility due to the very strong electrostatic interaction in the neutral ion pair ionic vapour. An excellent linear correlation is found between *ced*_IP_ and the inverse of the molecular volume. A good linear correlation is found between IL *ced*_IP_ and IL Gordon parameter (which are dependent primarily on surface tension). *ced* values obtained through indirect methods gave similar magnitude values to *ced*_IP_. These findings show that *ced*_IP_ is very important for understanding IL intermolecular interactions, in spite of *ced*_IP_ not being a measure of the total intermolecular interactions of an IL. In the outlook section, remaining challenges for understanding IL intermolecular interactions are outlined.

## 1. Why are intermolecular interactions of ionic liquids important?

Ionic liquids (ILs), liquids composed entirely of mobile cations and anions, are important from both an academic and an industrial standpoint [1,2]. ILs have been proposed as electrolytes, solvents for synthesis and catalysis, gas capture/storage media, lubricants, engineering fluids and in sensors [3–10].

The strength of intermolecular interactions have historically been used to understand and predict a range of properties for molecular liquids, including surface tension [11], wettability [12], the ability of a solvent to promote solute self-assembly [13,14], solubility [15] and viscosity [16–19]. All of these properties are very important for potential IL applications. For example, surface tension and wettability are key properties in understanding supercapacitors [20,21], and solubility is crucial for understanding and predicting gas separation/capture/storage [22]. Therefore, establishing links between these and other properties will give significant insight into the properties that underpin a wide range of applications.

A key property of ILs is their very low vapour pressure [23]. Hence, it is of interest to quantitatively investigate the intermolecular interactions of ILs to answer the fundamental question: do ILs have strong intermolecular interactions? In many publications it has been assumed that strong electrostatic intermolecular interactions drive the very low vapour pressures of ILs [24–27].

A pure molecular liquid contains only one constituent, the molecule; intermolecular interactions are controlled by molecule–molecule interactions. The situation is more complicated for ILs, as there are two constituents in each liquid: a cation and an anion. Both constituents will strongly influence the intermolecular interactions, making understanding intermolecular interactions of ILs much tougher than for molecular liquids. There are also many possible ILs and, therefore, many possible cation–anion combinations, providing an additional challenge to understanding intermolecular interactions of ILs.

In this article, attempts are made to find correlations for experimental measures of intermolecular interactions that hold across both molecular liquids and ILs; such correlations are particularly valuable, as these would reveal remarkable underlying similarities in intermolecular interactions. However, such correlations are expected to be very rare, given the very different chemical constituents of molecular liquids and ILs. Almost as valuable would be correlations for all ILs; such relationships that hold across ILs with a wide range of structural features are relatively rare. Lastly, family-based correlations can give significant insights into IL properties, although such correlations for relatively small groups of ILs have limited use for predicting IL properties. In this article the aim is to develop correlations for ILs using only experimental data. For certain values the required amount of experimental data does not yet exist. In such cases, quantitative correlations were not sought; however, qualitative observations were made. Data from simulations and calculations can provide an excellent complement to experimental data, and in some cases provide access to information that has not yet been experimentally measured.

In this article I use abbreviations to refer to individual ILs, instead of the IL names. The IL names, along with the relevant abbreviations, are given in the ESI, electronic supplementary material, table S1 (for cations) and electronic supplementary material, table S2 (for anions).

## 2. Quantifying the strength of intermolecular interactions of molecular liquids

### 2.1. How are the strengths of intermolecular interactions of molecular liquids quantified?

For molecular liquids the strength of intermolecular interactions is quantified using the cohesive energy density, *ced*_ML_: [28,29]

2.1 $${\text{ced}}_{\mathrm{ML}}=\frac{\Delta_{\mathrm{vap}}U}{V_{m}}=\frac{\text{ce}}{V_{m}}=\frac{\Delta_{\mathrm{vap}}H-RT}{V_{m}},$$

where Δ_vap_*U* is the internal energy of vaporization, i.e. the energy required to vaporize the liquid to its saturated vapour, *V*_m_ is the liquid molar volume (*V*_m_ = *M*/*ρ*, where *M* = molar mass and *ρ* = liquid density), *ce* is the cohesive energy, Δ_vap_*H* is the enthalpy of vaporization, *R* is the gas constant and *T* is the liquid temperature. *ced*_ML_ is a measure of the total intermolecular cohesion per unit volume in the liquid phase, assuming negligible intermolecular interactions in the vapour [28]. Alternatively, *ced*_ML_ quantifies the work required to produce a hole (often called a cavity) of unit volume in the molecular liquid [30].

### 2.2. Conditions used for vaporization measurements for molecular liquids

Vaporization is a physical process; no chemical change to form new products occurs. There are two types of vaporization: evaporation and boiling. Evaporation is a surface process; molecules escape from the liquid surface in the gas phase, where the gas phase is not saturated with the vapour of that molecule. Boiling is a bulk process; bubbles of saturated vapour are formed by molecules in the liquid phase. In this article the focus is on evaporation. ILs have been categorized as superheated liquids, and hence, observing IL boiling is very unlikely [31].

There are two extremes of vaporization conditions. Firstly, those close to equilibrium conditions, which are preferred for obtaining thermodynamic data. These conditions are generally produced using a Knudsen effusion cell, which is a heated source containing a pinhole to allow vapour to slowly escape under effusive conditions, thus giving near-equilibrium conditions. Secondly, those far from equilibrium conditions. Evaporation occurs from the liquid–gas surface and the vapour is detected directly (with no further interactions and no chance of condensation then re-evaporation of the vaporized sample); these conditions are known as free or Langmuir evaporation. Methods for measuring Δ_vap_*H* that involve a Knudsen effusion cell require higher sample temperatures (and, therefore, higher vapour pressures) than methods involving Langmuir vaporization (due to the effective smaller sample surface area available for vaporization). Higher sample temperatures also increase the probability of the chemical process of thermal decomposition (TD) occurring. For most molecular liquids the probability of vaporization, compared to the probability of TD, is sufficiently large (i.e. only sample vaporization occurs) that near-equilibrium methods are used to study vaporization, meaning that *ced*_ML_ can be readily determined for most molecular liquids [32].

For most molecular liquids there are negligible intermolecular interactions in the vapour, as they generally vaporize as individual isolated molecules. Therefore, *ced*_ML_ is a measure of the total intermolecular cohesion per unit volume in the liquid phase.

### 2.3. Results for the cohesive energy density for molecular liquids

*ced*_ML,298_ values range from 195 J cm^−3^ for squalane, through 385 J cm^−3^ for acetone, 679 J cm^−3^ for ethanol, 1055 J cm^−3^ for ethanolamine and up to 2293 J cm^−3^ for water [32]. Liquid hydrocarbons can be used to explain how Δ_vap_*H*_298_ and *ced* relate to intermolecular interaction strengths, and how size is vital. Δ_vap_*H*_298_ increases with increasing size, from *n-*hexane (Δ_vap_*H*_298_ = 31 kJ mol^−1^) to *n-*hexadecane (Δ_vap_*H*_298_ = 81 kJ mol^−1^) to squalane (Δ_vap_*H*_298_ = 105 kJ mol^−1^) [32,33]. However, clearly only van der Waals (vdW) interactions are added as the hydrocarbons increase in size. This fact is reflected in the *ced*_ML,298_ values, which are in the range of approximately 190 to approximately 270 J cm^−3^ for liquid hydrocarbons [32]. These *ced*_ML,298_ values are all very small, as expected when only vdW interactions are involved. Each liquid-phase squalane molecule forms a large number of relatively weak intermolecular interactions, all of which need to be broken in order to vaporize a squalane molecule. The increase in Δ_vap_*H*_298_ from *n-*hexane to squalane is not due to stronger intermolecular interactions; there are simply more intermolecular interactions per molecule (but not per unit volume).

Many molecular liquids have significant electrostatic interactions, e.g. water, glycerol, formamide, ethanolamine. For water Δ_vap_*H*_298_ = 44 kJ mol^−1^ and for formamide Δ_vap_*H*_298_ = 61 kJ mol^−1^, much lower than Δ_vap_*H*_298_ for *n-*hexadecane and squalene [32,33]. However, water and formamide are significantly smaller than *n-*hexadecane and squalene [32,33]. Therefore, water and formamide give much larger *ced*_ML_ values than for hydrocarbons [32], demonstrating that the intermolecular interactions in molecular liquids that have significant electrostatic interactions are much stronger than in hydrocarbons.

## 3. How the cohesive energy density relates to other properties for molecular liquids

Given the interest in correlating *ced* with other properties, it is vital to know how *ced*_ML_ values correlate to other liquid-phase properties.

### 3.1. Surface tension

Surface tension, *γ*, is a key parameter for colloid science and engineering [14]. Surface tension represents the energy required to separate a bulk liquid to create two new liquid–gas surfaces, i.e. the energy per surface area. Forming such surfaces requires breaking bulk intermolecular interactions [34–38]. In 1896, Stefan proposed that the work necessary to bring a molecule from the interior of the liquid to the surface is half that needed to vaporize it [37]. For samples that will thermally decompose before vaporization occurs, i.e. involatile liquids and polymers, the Gordon parameter, *G* [11], has been used as a substitute for *ced*: [14,39]

**Figure 1.**
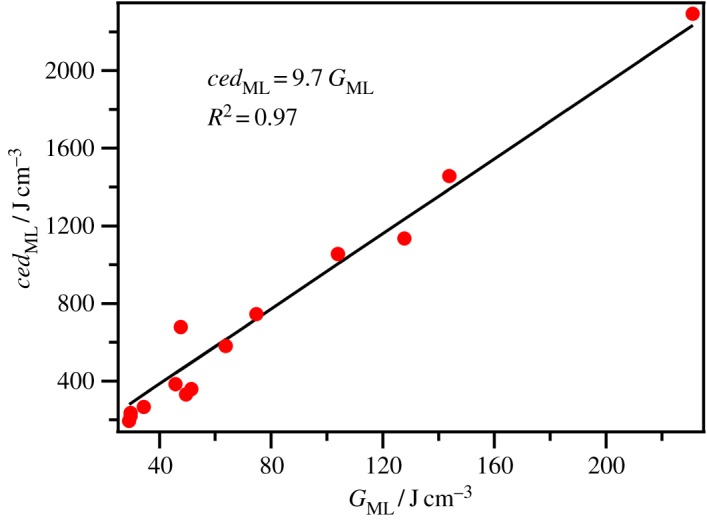
*ced*_ML_ versus *G*_ML_ for select molecular liquids.

3.1 $$G=\frac{\gamma }{V_{\mathrm{mol}}^{1/3}},$$

where *γ* is the surface tension and *V*_mol_ is the molecular volume (*V*_mol_ = *V*_m_/*N*_a_, where *N*_a_ = Avogadro's number). Owing to the possible influence of the surface structure on *γ* and therefore *G*, *ced* is generally preferred over *G* for investigating intermolecular interactions. However, for non-volatile samples *G* is easier to measure than Δ_vap_*H*. A very good linear correlation between *ced*_ML_ and *G*_ML_ is observed for a small selection of molecular liquids (figure 1).

### 3.2. Liquids as solvents for self-assembly

Solute self-assembly in solvents is vital in many areas, including in nature (in particular in water), drug delivery, inorganic materials and many more [13,14]. Until recently only approximately 20 solvents were known to promote self-assembly [40], although more have been found recently [41]. In particular, many protic ILs and a number of aprotic ILs have been found to promote self-assembly [40,42,43]. For ILs there are essentially two categories of cation: aprotic ILs are formed by transfer of an alkyl chain from e.g. an alkylchloride to a base, whereas protic ILs are formed by transfer of a proton from a Brønsted acid to a Brønsted base [44,45]. *ced* can be considered to represent the tightness or structuredness of liquids as caused by intermolecular interactions [30]. It has been demonstrated that structured liquids support self-assembly of amphiphilic molecules, due to the large *ced* [13,41]. Structural properties of liquids acting as solvents can be categorized by stiffness, as measured by the work that must be expended to create a hole in the liquid, as expressed by the liquid *ced* [46]. Evans has proposed that liquids with *G* (as a substitute for *ced*) > ∼ 110 J cm^−3^ tend to promote aggregation of amphiphilic molecules, i.e. *G* and *ced* can be used as measures of a solvent's solvophobic strength [13,14,47].

### 3.3. Solubility

Predicting the solubilities/miscibilities of gases/solvents in/with ILs is of vital importance [4,22], and a general prediction scheme would be of great use. Hildebrand showed that for non-polar liquids:

3.2 $$\delta_{H}={\text{ced}}^{0.5},$$

where *δ*_H_ is the Hildebrand solubility parameter [28]. *δ*_H_ is typically small for non-polar solvents and large for polar solvents [48]. If *δ*_H_ is similar for solvent and solute then it is predicted that solvation will occur, as similar strength interactions will be broken and formed. *δ*_H_ predicts solubility well for non-polar and slightly polar molecules, where vdW interactions are dominant. For systems where electrostatic interactions (e.g. polar liquids) are important, a one-component parameter such as *δ*_H_ does not represent the system sufficiently, and more components are required to explain solubility [15,49]. Hansen solubility parameters comprise: polar, non-polar and hydrogen bonding, and they can be estimated using indirect methods.

Activity coefficients give a measure of interactions between solute and solvent, and are used to obtain *δ*_H,indirect_ for liquids (*δ*_H_ values for ILs determined from activity coefficients are referred to in this article as *δ*_H,indirect_, to differentiate between *δ*_H_ values obtained from *ced*). Activity coefficients at infinite dilution for solutes in ILs have been measured using inverse gas chromatography, and *δ*_H,indirect_ was obtained (*ced* values for ILs determined from these *δ*_H,indirect_ values are referred to in this article as *ced*_indirect_) [50].

### 3.4. Viscosity

Viscosity is a key variable in process design, e.g. mixing, separation, lubrication. Viscous flow is often modelled by the activated jumping of a molecule from an initial configuration to a second position, separated by an intermediate activated state [18]. Eyring and co-workers postulated that the activated state of the molecule requires a larger volume than the initial state. As this larger volume requires hole creation in the liquid, the activation energy for viscous flow, *E*_a,vis_, should be a fraction of Δ_vap_*U*:

3.3 $$\Delta_{\mathrm{vap}}U=\text{ce}=\text{ced}.V_{m}=C.E_{a,\mathrm{vis}},$$

where *C* is a constant for ‘normal’ liquids. For non-spherical molecules (e.g. hexane and acetone) *C* ≈ 4; for small spherical molecules (e.g. CCl_4_ and cyclohexane) *C* ≈ 3. For both liquid metals and molten salts the unit of flow was considered to be important; to obtain a satisfactory linear correlation between Δ_vap_*U* and *E*_a,vis_, atomic/ionic radii were included in correlations [19,51]. A further possible complication is that the relationship between viscosity and temperature does not follow Arrhenius-like behaviour just above the glass transition temperature for all ILs (i.e. many ILs are fragile liquids), leading to possible errors in measured *E*_a,vis_ for ILs [52].

*δ*_H_, and therefore *ced*, can also be determined using measurements of intrinsic viscosity (*ced* values for ILs are again referred to as *ced*_indirect_). The viscosity of a dilute solution of a solute (e.g. a polymer) is a maximum in the ‘best’ solvent, i.e. the solution where cohesion properties of solvent and solute are comparable [53].

### 3.5. Internal pressure

The internal pressure of a liquid, *P*_int_, is another property that can be used to quantify intermolecular interactions. *ced*_ML_ depends on breaking all intermolecular interactions. *P*_int_ for molecular liquids depends on small isothermal expansion; intermolecular distances increase slightly so not all intermolecular interactions are broken. The ratio:

3.4 $$r=\frac{P_{\mathrm{int}}}{\text{ced}},$$

is the standard method to compare *P*_int_ to *ced*. *r* can be used to categorize solvents. Liquids where *r* > 1.2 are labelled as loose, e.g. fluorocarbons, triethylamine (repulsive interactions are relatively large) [54]. Liquids with *r* < 0.8 are collectively labelled as associated/tight/stiff liquids, e.g. water, ethanolamine (attractive interactions are strong) [54]. Liquids with *r* ∼ 1 are non-polar liquids, e.g. hydrocarbons, diethyl ether, toluene [28,54]. This ratio also highlights that *P*_int_ and *ced* have the same units, generally MPa or J cm^−3^.

## 4. Measuring the cohesive energy density for ionic liquids: overcoming the challenges

There are two major experimental challenges for obtaining *ced* for ILs (*V*_m_ can be readily measured with good accuracy for ILs [55]). Firstly, measuring the amount of IL ionic vapour at different temperatures to obtain Δ_vap_*H*. The inherently low vapour pressure of ILs makes these measurements difficult. For [C_2_C_1_Im][NTf_2_] at *T* = 298 K the vapour pressure can be estimated as approximately 10^−9^ Pa [56], a much lower value than the expected detection limits for any apparatus used for studying vaporization of molecular liquids. Secondly, the ionic vapour composition needs to be known under the conditions for which Δ_vap_*H* is measured (ionic vapour refers only to the vaporization of intact IL) [57], so that the nature of the intermolecular interactions broken is known.

### 4.1. Ionic liquids can be distilled

For many years, it was assumed that IL TD followed by vaporization of the non-ionic TD products occurred more readily than IL vaporization of intact ionic vapour. Hence, it was assumed that ILs had no detectable vapour pressure [58–60].

Since 2005 it has become accepted that ILs can be vaporized, in contradiction of previous assumptions. The major evidence for IL vaporization was their successful distillation, i.e. the physical process of IL vaporization, followed by condensation of the same species that was vaporized. Bulk-scale distillation of intact IL has been achieved for 1,3-dialkylimidazolium-, 1,1-dialkylpyrrolidinium- and tetraalkylammonium-based ILs at a temperature range of 475 to 575 K [61–63]. Most of the focus has been on [NTf_2_]^−^-based ILs, which have the required combination of relatively good thermal stability and relatively high volatility [31,64,65]. Aprotic ILs are the focus of this article, as very few vaporization studies of protic ILs have been published, in particular on identification of the vapour phase species [66–69].

### 4.2. The vapour phase composition of ionic liquids: the ionic vapour

In the seminal 2006 *Nature* article demonstrating distillation of ILs, it was speculated that IL vaporization occurred as isolated ions or as ion aggregates [61,70]. Additionally, a chemical process of IL transfer could not be ruled out.

#### 4.2.1. The ionic vapour: intact ions in the vapour phase

For a wide range of ILs using positive mode mass spectrometry (MS) (with electron ionization, field ionization or photoionization) the parent cation, [C]^+^ [57,63,66,71–89], has been detected intact in the vapour phase (at 350 K < *T* < 700 K). In addition, the parent anion, [A]^−^ [66,83,85], has been detected intact in the vapour phase using negative mode MS (with electron ionization or photoionization). The cations investigated include [C*_n_*C_1_Im]^+^, [C*_n_*C_1_Pyrr]^+^, [C*_n_*Py]^+^, tetraalkylphosphonium and alkylisouronium; the anions investigated include [NTf_2_] ^−^, [PF_6_] ^−^ and [TfO] ^−^. If IL TD products were vaporizing instead of intact IL vaporizing, one would not expect to detect intact parent cations and parent anions. For example, electron ionization MS of the products of heating [C*_n_*C_1_Im][A] ILs (A^−^ = halide ion) found non-ionic TD products such as 1-methylimidazole and alkyl halides [77,90]. Therefore, intact ionic has definitely been detected for a wide range of ILs. Leading on from this detection of intact parent ions in the mass spectra, there are two key questions to be answered about the ionic vapour.

#### 4.2.2. The ionic vapour is composed of neutral species, not isolated ions

For a wide range of ionic vapours, when the MS ionization source, e.g. electrons, was turned off, no ions were detected; it was concluded that the vapour phase of ILs is composed primarily of neutral species, not isolated ions [57,63,66,71–77]. More recently, using a specialist Knudsen effusion cell to produce the vapour of [C_2_C_1_Im][NTf_2_] and electric fields to extract any overall charged ions in the vapour phase, [C]^+^, [C_2_A]^+^, [A] ^−^ and [CA_2_] ^−^ were detected [87,91]. However, the amount of ions in the vapour phase was approximately 10^8^ to 10^11^ times lower than the amount of CA ion pairs [56], demonstrating the lack of significant amounts of overall charged ions in the vapour phase for [C_2_C_1_Im][NTf_2_]. Lastly, the intact molecular ion, [CA]^+•^ = [C_4_C_1_ImC(CN)_3_]^+•^, was detected for [C_4_C_1_Im][C(CN)_3_] using both field ionization and photoionization at *m/z* 229 [78,82], demonstrating that the lack of [CA]^+•^ detected for other ILs should not be taken as an indication of the lack of neutral vapour prior to ionization. Overall, these findings show that the vapours of the ILs studied to date are composed predominantly of neutral species.

#### 4.2.3. The ionic vapour is composed of one cation and one anion, not larger clusters

No higher mass clusters of the form [C*_m_*A*_m_*_–1_]^+^ (where *m* ≥ 2) have been observed after either electron ionization or photoionization of a wide range of ILs [57,63,66,71–77,79–82]. It has been suggested that for electron ionization too much excess energy is deposited into the ionic vapour, which will lead to the break-up of higher mass clusters such as C_3_A_3_ [92] (if such clusters are present in the ionic vapour). Vaporization as C*_m_*A*_m_* where *m* ≥ 2 occurs for alkali halides such as LiCl, and after electron ionization cluster ions such as [Li_3_Cl_2_]^+^ were detected [93]. This finding suggests that electron ionization MS may be able to detect higher mass clusters such as C_3_A_3_ (if such clusters are present in the vapour phase). For [C_4_C_1_Im][C(CN)_3_] the intact molecular ion, [CA]^+•^, has been detected using field ionization and photoionization [78,82], but no higher mass clusters were detected up to *m*/*z* 600; no [C_2_A]^+^ (*m*/*z* 368), [C_2_A_2_]^+•^ (*m*/*z* 458) or [C_3_A_2_]^+^ (*m*/*z* 597) were observed [82]. [CA]^+•^ is expected to have a significantly weaker intermolecular cation–radical interaction than the cation–anion intermolecular interactions in [C_2_A]^+^. Therefore, observation of [CA]^+•^ but not [C_2_A]^+^, [C_2_A_2_]^+•^ or [C_3_A_2_]^+^ shows that [C_2_A]^+^, C_2_A_2_ or [C_3_A_2_]^+^ were not present in significant concentrations in the ionic vapour.

Reactions of the form [C]^+^ + (ionic vapour) → (product ions) and [A] ^−^ + (ionic vapour) → (product ions) were carried out for [C_6_C_1_Im][NTf_2_], [P_6,6,6,14_][TfO] and [C_4_C_1_Pyrr][NTf_2_] [66]. [C_2_A]^+^ and [CA_2_] ^−^ were detected for the respective reactions, as would be expected if the ionic vapour was composed of CA neutral ion pairs. However, larger cluster ions, e.g. [C_3_A_2_]^+^, were not detected, demonstrating that the ionic vapour was not composed of C_2_A_2_ clusters, or any larger clusters.

Two ionization mechanisms have been proposed for ILs, demonstrated below for photoionization: dissociative ionization [71] (mechanism 1) and CA neutral ion pair dissociation [83] (mechanism 2):

mechanism 1 $$\begin{array}{rl} \text{CA}+h\nu & \to {\left[\text{CA}\right]}^{+∙}\to {\left[\text{C}\right]}^{+}+A^{∙} \end{array}$$

mechanism 2 $$\begin{array}{rl} \text{CA}+h\nu & \to {\left[\text{C}\right]}^{+}+{\left[\text{A}\right]}^{-} \end{array}$$

It should also be noted that one dicationic IL has successfully been vaporized. The ionic vapour was shown to consist of CA_2_ neutral ion triplets [94,95].

#### 4.2.4. The ionic vapour under equilibrium conditions

Most of the studies described in §§4.2.1 to 4.2.3 were performed under Langmuir vaporization conditions (for ILs with a wide range of different structures), i.e. not under equilibrium conditions [57,63,66,71–85]. Therefore, it is very important to highlight those studies that were performed under near-equilibrium conditions. A combination of a Knudsen effusion cell with a small orifice with MS detection has been used by a number of research groups to study the ionic vapour of [C*_n_*C_1_Im][NTf_2_] [86–89,91]. All evidence is in agreement with that given already in §4, demonstrating that the equilibrium ionic vapour for [C*_n_*C_1_Im][NTf_2_] ILs is composed of neutral ion pairs. At present ILs with other anions have not been successfully vaporized using a Knudsen effusion cell, e.g. [C*_n_*C_1_Im][PF_6_] ILs thermally decomposed in a Knudsen effusion cell [96].

When flash heating an IL/acetonitrile mixture, i.e. heating from room temperature to very high temperature very rapidly (nearly instantaneously to incandescence, expected to give *T* > 1000 K), larger clusters of the type [C_2_A]^+^ and [CA_2_] ^−^ were detected using MS [92]. It was suggested that these clusters were formed via gas-phase decomposition of neutral clusters, e.g. C_2_A_2_ [92]. However, such conditions are very far from equilibrium conditions and Δ_vap_*H* was not measured in this study; the composition of the ionic vapour at such temperatures does not appear to be relevant for Δ_vap_*H* measurements.

#### 4.2.5. Investigating the ionic vapour using simulations and calculations

Simulations and calculations in the temperature range relevant to IL experimental vaporization studies (360 K < *T* < 630 K, see electronic supplementary material, table S3) have shown mainly isolated, neutral ion pairs in the ionic vapour [97,98]. Simulations at temperatures well above those at which vaporization experiments have been carried out (i.e. *T* > 800 K) have suggested the presence of higher mass clusters [97,99–102]. In addition, below *T* approximately 350 K calculations found that the major constituent of the ionic vapour was C_4_A_4_ for [C_4_C_1_Im][CF_3_SO_3_] [97]. Measurements of Δ_vap_*H*_T_ have been made at a wide variety of different *T*, ranging from 370 to 625 K [57]. As will be explained in §4.4.2, extrapolations of Δ_vap_*H*_T_ are regularly made to *T* values for below those at which Δ_vap_*H*_T_ was measured, especially to *T* = 298 K. An important assumption for such extrapolations is that the ionic vapour remains the same at the target *T* as that *T* at which Δ_vap_*H*_T_ was measured. For example, to be confident in an extrapolation to *T* = 298 K, one needs to know what the ionic vapour composition is at 298 K. At present, the best effort to experimentally determine the ionic vapour composition near *T* = 298 K is at *T* = 340 K for [C_2_C_1_Im][NTf_2_], which suggested that the ionic vapour was composed of neutral ion pairs [57], although checking for C_4_A_4_ was not possible on the apparatus used due to *m*/*z* 1285 for [C_4_A_3_]^+^ being beyond the detector *m*/*z* range. Thus, there is currently no experimental evidence to confirm or deny the presence of clusters larger than ion pairs in the vapour of ILs at *T* = 298 K.

#### 4.2.6. Isolated ions in the ionic vapour

It has previously been stated that the enthalpy of desorption for an IL ion pair from the bulk IL, Δ_des_*H*(total), cannot be measured directly, which were reasonable statements at the time, given the very challenging nature of these experiments [103,104]. As noted in §4.2.2, for [C_2_C_1_Im][NTf_2_] high temperature-capable thermal ion emission MS has been used to detect ionic vapour species with net overall charge, i.e. very small quantities of isolated [C]^+^ and [A] ^−^ ions have been detected in the ionic vapour [87,91]. From measurements of the amount of [C]^+^ and [A] ^−^ ions in the equilibrium ionic vapour at different temperatures the enthalpy of cation desorption from the bulk IL, Δ_des_*H*([C]^+^) and the enthalpy of anion desorption from the bulk IL, Δ_des_*H*([A] ^−^) have been determined, respectively [87,91]. Therefore, experimental values for Δ_des_*H*([C]^+^) and Δ_des_*H*([A] ^−^) (and therefore, Δ_solv_*H*([C]^+^) and Δ_solv_*H*([A] ^−^)) have now be obtained [87,91].

#### 4.2.7. Summary

All evidence leads to the conclusion that vaporization occurs primarily as CA neutral ion pairs (i.e. no net overall charge), and vaporization as C*_m_*A*_m_* where *m* ≥ 2 does not occur to any significant level. There are very small amounts of isolated ions in the ionic vapour (i.e. species with net overall charge). There are open questions about the nature of the equilibrium ionic vapour composition for many ILs and the ionic vapour composition at room temperature; these will be outlined in §9.

### 4.3. Two different measures of cohesive energy density for ionic liquids

The molecular unit for an IL is made up of one cation and one anion, i.e. the molar mass of an IL is always taken from one cation and one anion, *M*_IP_. Much research has been carried out into the occurrence of ion pairs in bulk ILs; the general consensus is that long-lived ion pairs do not exist as individual entities in the bulk of ILs, at least at room temperature [105]. Therefore, the molecular units in the bulk IL (i.e. isolated ions, see §4.2) and in the ionic vapour (i.e. isolated neutral ion pairs) are different. As the bulk IL is made up of individual ions, the cation–anion interaction present in the neutral ion pair ionic vapour is deemed as intermolecular, as has been concluded elsewhere [104]. This judgement leads to two different versions of *ced* for ILs, here labelled *ced*_IP_ (*ced* when the ionic vapour is a neutral ion pair CA) and *ced*_C+A_ (*ced* when the ionic vapour is isolated ions, [C]^+^ and [A] ^−^).

**Scheme 1.**
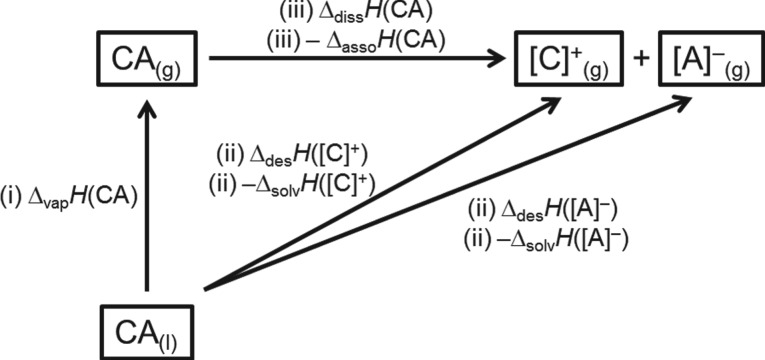
Extended Born–Fajans–Haber cycle for transformations of an ionic liquid in terms of enthalpies. Adapted from Preiss *et al.* [103].

**Scheme 2.**
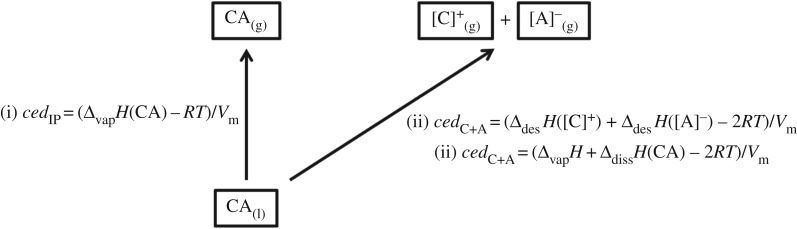
Transformations of an ionic liquid in terms of *ced*.

The traditional equation for *ced* (see equation (2.1)) can be used for ILs:

4.1 $${\text{ced}}_{\mathrm{IP}}=\frac{\Delta_{\mathrm{vap}}H-RT}{V_{m}},$$

where IP denotes ion pair, as the ionic vapour is a neutral ion pair (scheme 1, step (i)) and *V*_m_ is the IL molar volume (*V*_m_ = *M*_IP_/*ρ*, where *M*_IP_ = IL molar mass and *ρ* = IL density). Therefore, *ced*_IP_ of an IL can readily be obtained using Δ_vap_*H* (scheme 2, step (i)).

The energy to form isolated cations and isolated anions in the gas phase from the liquid phase can be obtained from Δ_des_*H*(total) (scheme 1, step (ii)). Δ_des_*H*(total) = −Δ_solv_*H*(total), where Δ_solv_*H*(total) represents the sum of the enthalpy of cation solvation from the vapour phase into the bulk IL, Δ_solv_*H*([C]^+^), and the enthalpy of anion solvation from the vapour phase into the bulk IL, Δ_solv_*H*([A] ^−^). *ced*_C+A_ can be obtained by measuring Δ_des_*H*(total) (scheme 2, step (ii)).

4.2 $$\begin{array}{rl} \Delta_{\mathrm{des}}H(\text{total}) & =\Delta_{\mathrm{des}}H({\left[\text{C}\right]}^{+})+\Delta_{\mathrm{des}}H({\left[\text{A}\right]}^{-}) \end{array}$$

4.3 $$\begin{array}{rl} {\text{ced}}_{C+A} & =\frac{\Delta_{\mathrm{des}}H(\text{total})-2RT}{V_{m}}. \end{array}$$

Isolated gas-phase cations and anions can also be formed by vaporizing a neutral ion pair (scheme 1, step (i)) and then breaking the cation–anion intermolecular interaction (scheme 1, step (iii)). Therefore, *ced*_C+A_ can be obtained by measuring Δ_vap_*H* and the enthalpy of vapour phase neutral ion pair dissociation, Δ_diss_*H*(CA) (scheme 1, step (ii)).

4.4 $$\begin{array}{rl} \Delta_{\mathrm{des}}H(\text{total}) & =\Delta_{\mathrm{vap}}H+\Delta_{\mathrm{diss}}H(\text{CA}) \end{array}$$

4.5 $$\begin{array}{rl} {\text{ced}}_{C+A} & =\frac{\Delta_{\mathrm{vap}}H+\Delta_{\mathrm{diss}}H(\text{CA})-2RT}{V_{m}}. \end{array}$$

As there are significant intermolecular interactions in the vapour, *ced*_IP_ does not capture all liquid-phase intermolecular interactions for an IL, i.e. *ced*_IP_ is not a measure of the total intermolecular cohesion per unit volume in the IL. *ced*_C+A_ captures all liquid-phase intermolecular interactions for an IL, i.e. *ced*_C+A_ is a measure of the total intermolecular cohesion per unit volume in the IL.

### 4.4. Problems caused by the low vapour pressure of ionic liquids

#### 4.4.1. Monitoring the amount of ionic liquid vaporized

Accurately detecting the very small amounts of IL vaporization with respect to temperature is very challenging experimentally, especially with apparatus developed for molecular liquids. Hence, methods have been developed to measure IL vaporization at the temperatures required. Improvements in the sensitivity of vaporization detection mean that measurements have been made at increasingly lower temperature; therefore, TD is expected to be less of a complication for measurements made at lower temperatures.

**Scheme 3.**
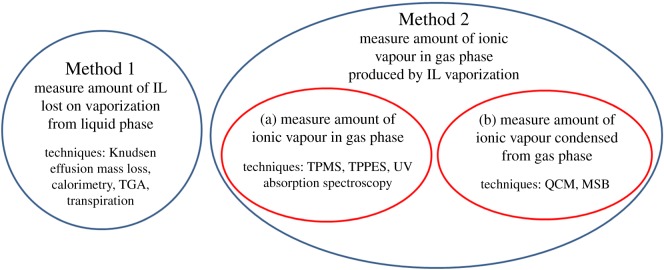
The two methods used to monitor the amount of IL vaporized with respect to temperature. Within Method 2, there are two approaches to determine the amount of ionic vapour in the gas phase. TGA, thermogravimetric analysis; TPMS, temperature-programmed mass spectrometry; TPPES, temperature-programmed photoelectron spectroscopy; QCM, quartz crystal microbalance; MSB, magnetic suspension balance.

There are broadly two methods to monitor the amount of IL vaporized with respect to temperature (scheme 3). Method 1: measure the amount of IL lost on vaporization from the liquid phase. Techniques that can be used to monitor the mass lost as the IL vaporizes include Knudsen effusion mass loss [56,88], calorimetry [106,107], thermogravimetric analysis (TGA) [31,64,65,108–116] and transpiration [117]. Method 2: measure the amount of ionic vapour in the gas phase produced by IL vaporization. Within Method 2, there are two approaches to determine the amount of ionic vapour in the gas phase: (a) measure the amount of ionic vapour in the gas phase (temperature-programmed MS (TPMS) [57,63,71–77,86], temperature-programmed photoelectron spectroscopy (TPPES) [80], UV absorption spectroscopy [118]), (b) measure the amount of ionic vapour that has condensed from the gas phase onto a solid surface (quartz crystal microbalance (QCM) [96,110–112,114–116,119–136], magnetic suspension balance (MSB) [31]).

#### 4.4.2. Comparing measured Δ_vap_*H*_T_: adjusting Δ_vap_*H*_T_ to a common temperature

Measurements of Δ_vap_*H*_T_ have been made at a wide range of *T*, from 370 to 625 K [57]. To make valid comparisons of Δ_vap_*H*_T_ a common *T* is needed. To obtain Δ_vap_*H*_T_ at a constant *T* (usually *T* = 298 K) an extrapolation is required from the measurement *T* to the constant *T* used for Δ_vap_*H*_T_ comparisons. Such an extrapolation requires knowledge of the heat capacity at constant pressure, Δ^g^_l_*C*_p_, between the IL in the vapour and liquid phases, over the *T* range of interest. It has been noted that this extrapolation was a major source of discrepancies for published Δ_vap_*H*_298_ values for ILs [111]. For ILs, the liquid phase heat capacities can be readily measured [137]. However, the gas-phase heat capacities have not been measured to date, and have been judged to be impossible to measure [111]. Thus, different methods of obtaining Δ^g^_l_*C*_p_, have been proposed. However, the variety of different methods and Δ^g^_l_*C*_p_ values used means that a summation is required.

Quantum mechanical and statistical thermodynamic calculations on ionic vapour neutral ion pairs have been used to obtain the gas-phase heat capacity for [C*_n_*C_1_Im][PF_6_] [138]. Based upon this value, a value for Δ^g^_l_*C*_p_ in the region of −100 J K^−1^ mol^−1^ has been used for a wide variety of ILs [56,57,63,71–73,75,77,108,117,118,122,123,131,134,136]. Quantum mechanical and statistical thermodynamic calculations on ionic vapour neutral ion pairs have been used to obtain gas-phase heat capacities for [C*_n_*C_1_Im][NTf_2_] (*n* = 2, 4, 6, 8) [139]. Correlations developed from these values have then been used to obtain Δ^g^_l_*C*_p_ for [C*_n_*C*_m_*Im][NTf_2_] ILs, ranging from Δ^g^_l_*C*_p_ = −112 J K^−1^ mol^−1^ for [C_2_C_1_Im][NTf_2_] to Δ^g^_l_*C*_p_ = −203 J K^−1^ mol^−1^ for [C_10_C_10_Im][NTf_2_] [129,130,132,133,135]. Clearly, there are very large and unsatisfactory discrepancies between these Δ^g^_l_*C*_p_ values and the widely used Δ^g^_l_*C*_p_ = −100 J K^−1^ mol^−1^ value; these differences can lead to significant variation in Δ_vap_*H* values, up to 20 kJ mol^−1^ for ILs with long alkyl chains [111].

In 2013, Verevkin *et al.* [111] analysed their Δ_vap_*H* data for [C*_n_*C_1_Im][NTf_2_] (*n* = 1–18) measured using two different techniques, TGA and QCM. As the *T* at which the Δ_vap_*H*_T_ data were recorded using the two techniques were different by approximately 150 K, values for Δ^g^_l_*C*_p_ could be indirectly determined using this experimental Δ_vap_*H*_T_ data. Δ^g^_l_*C*_p_ was found to range from Δ^g^_l_*C*_p_ = −56 J K^−1^ mol^−1^ for *n* = 2 to Δ^g^_l_*C*_p_ = −170 J K^−1^ mol^−1^ for *n* = 16. This procedure using variable −*T* Δ_vap_*H*_T_ data has since been applied to more ILs, although to date they have all been for ILs with an [NTf_2_] ^−^ anion [88,106,114–116]; for example, Δ^g^_l_*C*_p_ = −67 J K^−1^ mol^−1^ for *n* = 4 has been found, in excellent agreement with the findings of Verevkin and co-workers [88]. Overall, Δ^g^_l_*C*_p_ has generally been found to be in the range of −40 to −120 J K^−1^ mol^−1^ for ILs with relatively few CH_2_ groups, whereas for ILs with relatively large amounts of CH_2_ groups Δ^g^_l_*C*_p_ has generally been found to be in the range of −120 to −200 J K^−1^ mol^−1^. All groups have found a chain length dependence of Δ^g^_l_*C*_p_. Most recently, Δ^g^_l_*C*_p_ = −62 J K^−1^ mol^−1^ has been proposed using a combination of experimental Δ_vap_*H*_T_ data measured at different *T* and educated guesses [140]. When compared to Δ^g^_l_*C*_p_ derived from both methods explained above (calculations and indirectly from experimental data), Δ^g^_l_*C*_p_ = −62 J K^−1^ mol^−1^ appears too small for ILs with relatively large numbers of CH_2_ groups, e.g. [C_12_C_1_Im][NTf_2_] and [C_6_C_6_Im][NTf_2_].

Overall, there are two possible options for obtaining Δ_vap_*H*_T_ at a common *T*. Firstly, use Δ^g^_l_*C*_p_ values defined in the literature where available, and otherwise use a constant, IL independent Δ^g^_l_*C*_p_ value. This mix-and-match approach has the advantage of including the expected alkyl chain dependence of Δ^g^_l_*C*_p_ for certain ILs [111]; the clear disadvantage is the glaring inconsistency, as Δ^g^_l_*C*_p_ has not been determined by any method for many ILs for which Δ_vap_*H* values have been measured. The second option is to use a constant, IL independent Δ^g^_l_*C*_p_ value for all ILs. This approach has the advantages of simplicity and consistency, but with the considerable disadvantage of missing the alkyl chain length dependence. In this contribution a constant, IL independent Δ^g^_l_*C*_p_ = −100 J K^−1^ mol^−1^ was used, as this approach is a reasonable compromise, as it represents the approximate mid-point of Δ^g^_l_*C*_p_ values published to date.

In terms of selecting a common *T*, in almost all literature examples for ILs *T* = 298 K has been chosen; thus, Δ_vap_*H*_298_ is obtained. Using *T* = 298 K allows ready comparison to many other properties of ILs, e.g. surface tension. However, *T* = 450 K has also been used to ensure that the *T* extrapolation is minimal, as *T* = 450 K is similar to the *T* at which Δ_vap_*H* is measured [140]. In this contribution *T* = 298 K is used. As noted above, when using *T* = 298 K and Δ^g^_l_*C*_p_ = −100 J K^−1^ mol^−1^, large differences can occur for certain ILs. However, *T* = 450 K was also tested; using *T* = 450 K gave the same correlation observed in figure 3*b* (see electronic supplementary material, figure S1).

#### 4.4.3. Monitoring ionic liquid vaporization only

IL vaporization only must be measured to ensure reliable Δ_vap_*H* data, i.e. the vaporization of liquid-phase non-ionic TD products must not be measured. Methods that can distinguish *in situ* between IL vaporization and vaporization of liquid-phase TD products are ideal. However, there are relatively few such methods available; for ILs, TPMS and TGA (using two carrier gases) are the main methods used for distinguishing IL vaporization and vaporization of liquid-phase TD products [31,64,65,77].

Methods that involve measuring the amount of vapour that condenses from the gas phase onto a solid surface (e.g. QCM and MSB), when operated under high vacuum conditions (i.e. apparatus base system pressure less than 10^−8^ mbar), can be used to detect only IL vaporization. ILs are clearly sufficiently involatile that they will condense onto a solid surface at room temperature, whatever the system pressure. Many TD products of ILs are highly volatile, e.g. for halide-ion ILs [77,90]. Therefore, it is expected that such volatile TD products will not condense under high vacuum conditions at room temperature. Consequently, using QCM and MSB under high vacuum conditions would detect only IL vaporization, as long as the TD products were relatively volatile. However, not all TD products for ILs are highly volatile, e.g. for [C*_n_*C_1_Im][BF_4_] ILs [141].

A number of popular methods of obtaining Δ_vap_*H* for ILs involve measuring the total amount of vaporization. Almost all methods that involve measuring the mass lost from the liquid-phase measure the total amount of IL vaporization, e.g. TGA using only one carrier gas [108–116], Knudsen effusion mass loss [56] and calorimetry [142]. Also, some methods that involve measuring the amount of ionic vapour rely on there being no significant vaporization of liquid-phase non-ionic TD products, e.g. vapour phase spectroscopy [80,118]. Therefore, these methods rely on no significant vaporization of liquid-phase TD products occurring. In some studies attempts have been made to check if TD occurred by characterizing either the condensate or the remaining IL that had not vaporized. Neither of these approaches is foolproof. For the condensate, as noted above, TD products may be sufficiently volatile that they do not condense under experimental conditions; for the remaining IL sample, observing no TD products in the liquid phase does not mean there was no TD product vaporized.

For [C*_n_*C_1_Im][NTf_2_] ILs (*n* = 2 and 4) it has been demonstrated, using two carrier gas TGA, that Δ_vap_*H* is far smaller than the activation energy of TD [31,64]. In addition, [C*_n_*C_1_Im][NTf_2_] ILs (along with [N_2,2,2,2_][NTf_2_] and [C_4_C_1_Pyrr][NTf_2_]) have been readily distilled without significant TD occurring [61–63]. These results indicate that for [cation][NTf_2_] ILs, where the cation does not contain relatively reactive functional groups, only IL vaporization is significant under the experimental conditions generally used for vaporization experiments. Therefore, the assumption that many researchers make that only IL vaporization occurs for [cation][NTf_2_] ILs appears to be valid, making Δ_vap_*H* values measured for [cation][NTf_2_] more trustworthy than for other ILs.

There is the absurd situation where the same technique, TGA with a single carrier gas, for the same families of ILs has been used to determine either Δ_vap_*H* or the activation energy of TD, i.e. in different papers it is assumed that only IL vaporization or only liquid-phase non-ionic TD product vaporization was occurring. This situation has occurred for carboxylate-based ILs [143,144], [NTf_2_] ^−^-based ILs [110,111,145,146] and Cl^−^-based ILs [147,148]. Therefore, when using TGA it would appear best to use at least two different carrier gases (to allow ionic vapour and TD products to be distinguished) or only study ILs for which vaporization is known to be dominant, e.g. [cation][NTf_2_]. Hence, for the correlations discussed in §§7 and 8, Δ_vap_*H* values measured using TGA (and the transpiration method) for ILs other than [cation][NTf_2_] ILs have been excluded. This exclusion of ILs such as [cation]Cl reflects the unknown composition of the vapour phase; such ILs are known to have relatively similar Δ_vap_*H* and the activation energies of TD, and are known to thermally decompose, even under vacuum using Langmuir vaporization conditions [77,90]. For the correlations by Kabo *et al*. [140] of literature Δ_vap_*H* values with *ρ* and *γ*, all TGA data were excluded due to uncertainty over the data analysis methods. However, in this article TGA data were selected based upon the ILs studied and concerns over competition from TD; the analysis methods used were trusted.

The Δ_vap_*H* value from the group of Vaghjiani and co-workers for [C_4_C_1_Pyrr][NTf_2_] is excluded from correlations here, as it appears to be a very large outlier [80] relative to the other three published Δ_vap_*H* values for the same IL [73,112]. The microcalorimetry Δ_vap_*H* values for [C*_n_*C_1_Im][NTf_2_] are very large outliers [142] and were excluded for reasons explained in [129]. A contributing factor may have been the relatively high temperatures at which these measurements were carried out. The TPUV Δ_vap_*H* data from Ogura *et al*. [149] was excluded due to the very large Δ_vap_*H* values and the very large errors given.

The Δ_vap_*H* values from Verevkin and co-workers for three protic ILs are excluded [150]. It is expected that the vapour phase for the three ILs studied will mainly comprise ionic vapour [67] and not non-ionic TD product vapour (most likely the products of proton transfer from the cation to the anion), but no evidence is presented to confirm this supposition. For protic ILs, ideally a Δ_vap_*H* technique would be used that can distinguish between IL vaporization and TD product vaporization.

#### 4.4.4. Comparing vaporization conditions: Knudsen effusion versus Langmuir

For ILs, both Knudsen effusion [56,86–89,91,119,129–133,135,151,152] and Langmuir [31,57,63,64,71–77,80,96,106–128] vaporization methods have been used to obtain Δ_vap_*H*. For Langmuir (i.e. non-equilibrium) vaporization, an activation barrier to vaporization due to the IL–gas surface structure could exist. Consequently, measurements under Langmuir vaporization conditions would not probe the vaporization energy required to obtain a reliable and accurate Δ_vap_*H* value, i.e. vaporization kinetics would be contributing to any measurement.

Any possible effect of kinetics on vaporization energetics can be judged by comparing data for techniques that used near-equilibrium and far-from-equilibrium methods, e.g. Knudsen mass loss versus TPMS, respectively. For [C*_n_*C_1_Im][NTf_2_], Δ_vap_*H* values published in 2006 and 2007 from Knudsen cell mass loss [56] and from Langmuir vaporization [71] matched extremely well (note that the Δ^g^_l_*C*_p_ values used were very similar and thus no mismatch was caused by Δ^g^_l_*C*_p_). Since then, both near-equilibrium and Langmuir methods have confirmed agreement in Δ_vap_*H* values for [C*_n_*C_1_Im][NTf_2_] [111,129]. That the values are the same within the relatively small errors strongly suggests that there is no significant kinetic effect for Langmuir vaporization; therefore, there is no significant activation barrier for vaporization at the IL–gas surface. Good matches of Δ_vap_*H* values from near-equilibrium and far-from-equilibrium methods have also been published for [C*_n_*C_1_Pyrr][NTf_2_] and [C*_n_*Py][NTf_2_] ILs. For ILs with anions other than [NTf_2_] ^−^ there is currently no data to judge kinetic effects on vaporization, as no Knudsen cell (i.e. near-equilibrium) measurements have been made for ILs with anions other than [NTf_2_] ^−^. For example, for [C_4_C_1_Im][PF_6_] the primary process for near-equilibrium conditions was vaporization of TD products [153], whereas the primary process for Langmuir evaporation was vaporization of intact IL [96]. This difference is due to the higher temperature required for near-equilibrium conditions (compared to Langmuir evaporation) to detect vaporization.

#### 4.4.5. Δ_vap_*H* values for approximately 115 ionic liquids

There is now a significant quantity of Δ_vap_*H* data available in the literature (approx. 40 papers); there are Δ_vap_*H*_298_ values published for approximately 115 ILs (see electronic supplementary material, table S3). Δ_vap_*H* values published in the literature to date have mainly been produced by three groups, who are led principally by Verevkin, Santos and Jones. The Santos group have published only for [cation][NTf_2_] ILs, whereas the Verevkin and Jones groups have published for [cation][NTf_2_] ILs and also for a wider variety of anions, e.g. [PF_6_] ^−^ and [BF_4_] ^−^. Publications from groups other than these three groups have mainly focused on [cation][NTf_2_] ILs. This concentration on [cation][NTf_2_] ILs reflects firstly the wish for researchers new to the area to investigate ILs for which Δ_vap_*H* values have already been published, secondly the relative thermal stability of [cation][NTf_2_] ILs (as noted in §4.4.3, and quantified in [31,64]) and thirdly the large number of [cation][NTf_2_] ILs that are liquid at room temperature.

#### 4.4.6. Measuring Δ_des_*H*

Measuring *ced*_C+A_ requires *V*_m_ and either Δ_des_*H*([C]^+^) and Δ_des_*H*([A] ^−^) (equation (4.3)) or Δ_vap_*H* and Δ_diss_*H*(CA) (equation (4.5)). Therefore, there are two potential experimental approaches to obtain *ced*_C+A_.

Experimental values for Δ_des_*H*([C]^+^) and Δ_des_*H*([A] ^−^) can be obtained [87,91]. These measurements have been carried out in the range of *T* approximately 490 K. To obtain Δ_des_*H*([C]^+^) and Δ_des_*H*([A] ^−^) at *T* = 298 K, an extrapolation is required. Therefore, the heat capacity at constant pressure for the isolated ions in the vapour phase will be required (the heat capacity for the liquid phase can be measured, as noted in §4.4.2). At present, these values are not available, although they could be readily calculated.

Δ_diss_*H*(CA) has not been measured experimentally to date. For a [C_2_C_1_Im][NTf_2_] ionic vapour neutral ion pair the energy to produce electronically excited but bound ([C_2_C_1_Im][NTf_2_])* was found to be 5.5 eV, i.e. 531 kJ mol^−1^ [85]. This value represents an upper limit for Δ_diss_*H*(CA) for [C_2_C_1_Im][NTf_2_].

### 4.5. Obtaining the cohesive energy density from simulations and calculations

Δ_vap_*H* can be obtained using simulations and calculations. Enthalpies for the ionic vapour neutral ion pair can be calculated using high-level methods. The most widely used approach to obtain the liquid-phase enthalpy per ion pair is from molecular dynamics simulations [26,154–156]; an ion pair in a continuum solvation model has also been used [157,158]. *V*_m_ can also readily be obtained from molecular dynamics simulations; hence, *ced*_IP_(calc.) can be obtained from simulations and calculations for ILs.

Δ_des_*H*(total) (i.e. *ce*_C+A_ + 2*RT*) can be obtained either from a combination of experimental (Δ_vap_*H*, table 1) and calculated (Δ_diss_*H*_298_(CA), table 2) [103] results or solely from calculations [104]. Δ_diss_*H*(CA) values have been published for many ILs (e.g. [103,196,197]). A combination of simulations and calculations has been used for ILs to obtain *ce*_C+A_ for a limited selection of ILs ([C*_n_*C_1_Im][BF_4_] and [C*_n_*C_1_Im][PF_6_], where *n* = 2, 4, 6) [104]; *ced*_C+A_ were not provided in the article.

**Table 1.** Δ_vap_*H*_298_, *V*_m_ (at 298 K), *V*_mol_ (at 298 K), *γ* (at 298 K), *G* (at 298 K) and *ced*_IP,298_ data for ILs (for which all of this data could be found in the literature from experimental measurements). For Δ_vap_*H*_298_ and *ced*_IP,298_ a constant Δ^g^_l_*C*_p_ = −100 J K^−1^ mol^−1^ value was used in all cases. Δ_vap_*H*_298_ values were determined for each IL by taking the average (see electronic supplementary material, table S4) of all reliable individual Δ_vap_*H*_298_ literature values (how these individual Δ_vap_*H*_298_ values were chosen is explained in §4.4.3). All literature references used to obtain Δ_vap_*H*_298_, *V*_m_, *V*_mol_ (liquid density, *ρ*, for both *V*_m_ and *V*_mol_) and *γ* are given. .

ionic liquid	Δ_vap_*H*_298_kJ mol^−1^	ref. for Δ_vap_*H*_298_	*V*_m_cm^−3^ mol^−1^	*V*_mol_nm^−3^	ref. for *ρ*	*γ*mN^−1^ m^−1^	ref. for *γ*	*G *J cm^−3^	*ced*_IP,298_J cm^−3^
[C_1_C_1_Im][NTf_2_]	132	[111,132]	241	0.400	[55]	36.3	[55]	49.3	539
[C_2_C_1_Im][NTf_2_]	136	[56,57,71,86,106,108,111,117,118,129]	257	0.427	[159]	36.9	[160]	49.1	519
[C_3_C_1_Im][NTf_2_]	131	[111,129]	275	0.456	[161]	34.9	[160]	45.3	469
[C_4_C_1_Im][NTf_2_]	136	[56,71,88,108,111,113,118,129]	292	0.486	[55]	33.6	[160]	42.8	457
[C_5_C_1_Im][NTf_2_]	138	[111,129]	309	0.513	[161]	32.9	[160]	41.1	439
[C_6_C_1_Im][NTf_2_]	142	[56,71,108,111,129]	328	0.545	[55]	32.3	[160]	39.6	425
[C_7_C_1_Im][NTf_2_]	143	[111,129]	341	0.567	[162]	32.0	[160]	38.7	412
[C_8_C_1_Im][NTf_2_]	149	[56,71,108,111,129]	363	0.603	[55]	31.9	[160]	37.8	403
[C_10_C_1_Im][NTf_2_]	152	[108,111,129]	397	0.659	[55]	32.1	[160]	36.9	378
[C_12_C_1_Im][NTf_2_]	156	[111,129]	427	0.710	[55]	32.3	[163]	36.2	358
[C_3_C_1_C_1_Im][NTf_2_]	142	[86,108,152]	288	0.478	[164]	41.0	[165]	52.4	485
[C_4_C_1_C_1_Im][NTf_2_]	145	[121,152]	306	0.509	[166]	37.4	[167]	46.9	466
[C_3_(C_1_Im)_2_][NTf_2_]_2_	183	[94]	476	0.791	[168]	44.7	[168]	48.3	378
[Me(EG)_1_C_1_Im][NTf_2_]	130	[115]	280	0.465	[169]	35.6	[169]	45.9	455
[Me(EG)_2_C_1_Im][NTf_2_]	136	[115]	320	0.531	[55]	36.5	[55]	45.1	419
[C_2_C_2_Im][NTf_2_]	126	[130]	275	0.457	[170]	35.6	[163]	46.2	450
[C_3_C_3_Im][NTf_2_]	131	[130]	310	0.515	[170]	32.4	[163]	40.4	416
[C_4_C_4_Im][NTf_2_]	134	[130]	344	0.571	[170]	31.1	[163]	37.5	383
[C_5_C_5_Im][NTf_2_]	142	[130]	379	0.629	[170]	30.1	[163]	35.1	368
[C_6_C_6_Im][NTf_2_]	157	[130]	412	0.685	[170]	29.5	[163]	33.5	373
[C_7_C_7_Im][NTf_2_]	150	[133]	445	0.740	[170]	29.3	[163]	32.4	332
[C_8_C_8_Im][NTf_2_]	146	[133]	480	0.798	[170]	29.2	[163]	31.5	298
[C_9_C_9_Im][NTf_2_]	150	[133]	515	0.855	[170]	29.5	[163]	31.1	286
[C_10_C_10_Im][NTf_2_]	146	[133]	547	0.908	[170]	29.6	[163]	30.6	262
[C_3_C_2_Im][NTf_2_]	132	[132]	292	0.485	[171]	33.4	[171]	42.5	445
[C_3_C_1_Pyrr][NTf_2_]	147	[112]	284	0.472	[172]	32.5	[167]	41.8	510
[C_4_C_1_Pyrr][NTf_2_]	150	[73,112]	300	0.498	[172]	34.9	[167]	44.1	490
[C_6_C_1_Pyrr][NTf_2_]	152	[73,112]	341	0.567	[173]	31.7	[173]	38.3	438
[C_10_C_1_Pyrr][NTf_2_]	161	[112]	405	0.673	[173]	31.4	[173]	35.8	391
[C_2_Py][NTf_2_]	138	[124,131]	253	0.420	[174]	37.4	[174]	50.0	536
[C_4_Py][NTf_2_]	143	[118,124,131]	286	0.476	[174]	33.4	[174]	42.8	491
[C_5_Py][NTf_2_]	144	[124]	303	0.503	[174]	32.5	[174]	40.9	469
[C_6_Py][NTf_2_]	151	[73,124]	320	0.532	[174]	31.7	[174]	39.1	463
[C_2_C_1_Im][NPf_2_]	136	[108,118]	308	0.512	[175]	33.9	[175]	42.4	432
[C_4_C_1_Im][NPf_2_]	137	^104, 114^	343	0.570	[175]	31.7	[175]	38.2	392
[C_6_C_1_Im][NPf_2_]	139	[108]	378	0.627	[175]	30.3	[175]	35.4	361
[C_8_C_1_Im][NPf_2_]	145	[108]	412	0.685	[55]	27.7	[55]	31.4	346
[C_2_C_1_Im][BF_4_]	143	[73,123]	155	0.257	[175]	54.4	[175]	85.5	905
[C_4_C_1_Im][BF_4_]	154	[75]	188	0.313	[175]	46.9	[175]	69.1	806
[C_8_C_1_Im][BF_4_]	163	[71]	257	0.427	[55]	30.8	[55]	40.9	626
[P_6,6,6,14_][BF_4_]	199	[75]	605	1.005	[176]	28.3	[176]	28.2	325
[C_4_Py][BF_4_]	161	[73,127]	184	0.305	[177]	46.6	[177]	69.2	860
[^3^C_1_^1^C_4_Py][BF_4_]	153	[127]	200	0.333	[178]	44.8	[178]	64.7	752
[^4^C_1_^1^C_4_Py][BF_4_]	152	[127]	200	0.333	[178]	45.5	[178]	65.6	746
[C_2_C_1_Im][C_2_SO_4_]	155	[57,123]	191	0.317	[179]	47.0	[179]	68.9	800
[C_2_C_1_Im][C_8_SO_4_]	172	[123]	292	0.485	[180]	31.0	[180]	39.5	580
[C_4_C_1_Im][C_8_SO_4_]	182	[75]	328	0.544	[181]	26.7	[181]	32.7	548
[C_2_C_1_Im][TfO]	138	[123]	188	0.312	[182]	41.3	[183]	60.9	721
[C_8_C_1_Im][TfO]	152	[71]	302	0.501	[55]	28.5	[55]	35.9	495
[C_4_C_1_Im][PF_6_]	150	[96]	207	0.345	[173]	44.0	[184]	62.8	710
[C_6_C_1_Im][PF_6_]	153	[96]	241	0.401	[185]	38.2	[184]	51.8	624
[C_8_C_1_Im][PF_6_]	164	[71,96]	276	0.458	[55]	32.5	[55]	42.2	585
[C_2_C_1_Im][SCN]	153	[73,123]	152	0.252	[186]	57.8	[186]	91.5	993
[C_8_C_1_Im][N(CN)_2_]	163	[73]	256	0.426	[187]	36.9	[188]	49.0	627
[C_4_C_1_Pyrr][N(CN)_2_]	162	[72]	198	0.329	[189]	56.4	[189]	81.7	806
[C_2_C_1_Im][C(CN)_3_]	139	[122]	186	0.308	[190]	50.4	[191]	74.6	733
[C_4_C_1_Im][C(CN)_3_]	143	[122]	218	0.363	[190]	49.2	[191]	69.0	643
[C_2_C_1_Im][B(CN)_4_]	136	[123]	217	0.361	[192]	48.7	[191]	68.4	613
[C_4_C_1_Im][FeCl_4_]	171	[73]	247	0.410	[193]	46.0	[193]	62.0	684
[C_2_C_1_Im][FAP]	126	[123]	342	0.568	[194]	32.5	[194]	39.2	361
[C_6_C_1_Im][FAP]	144	[75]	395	0.656	[194]	31.6	[194]	36.4	359
[C_4_C_1_Pyrr][FAP]	153	[73]	370	0.614	[195]	38.0	[165]	44.7	408
[C_8_C_1_Im]Cl	167	[77]	229	0.380	[55]	30.9	[55]	42.7	718
[C_8_C_1_Im]I	167	[77]	247	0.410	[55]	32.7	[55]	44.0	667

**Table 2.** Δ_vap_*H*_298_ (constant Δ^g^_l_*C*_p_) and *ced*_IP,298_ (constant Δ^g^_l_*C*_p_) are taken from table 1. Δ_diss_*H*_490_(CA) (exp.) = Δ_des_*H*_490_(total) − Δ_vap_*H*_490_. Δ_diss_*H*_298_(CA) (calc.) are taken from Preiss *et al.* [103]. *ced*_C+A_(exp. + calc.) have been rounded to three significant figures.

ionic liquid	Δ_vap_*H*_298_(exp.) kJ mol^−1^	*ced*_IP,298_(exp.) J cm^−3^	Δ_diss_*H*_298_(CA) (calc.)kJ mol^−1^	Δ_des_*H*_298_ (total) (calc.) kJ mol^−1^	*ced*_C+A,298_ (exp. + calc.) J cm^−3^
[C_2_C_1_Im][NTf_2_]	136	519	344	480	1850
[C_4_C_1_Im][NTf_2_]	136	457	351	487	1650
[C_2_C_1_Im][BF_4_]	143	905	372	515	3290
[C_4_C_1_Im][BF_4_]	154	806	372	526	2770
[C_4_C_1_Im][PF_6_]	150	710	353	503	2400
[C_2_C_1_Im][C_2_SO_4_]	155	800	385	540	2800
[C_2_C_1_Im][SCN]	153	993	371	524	3420
[C_2_C_1_Im][C(CN)_3_]	139	733	340	479	2550

As explained in §3.3, *δ*_H_ (and therefore *ced*_ML_) can be separated, using indirect methods, into non-polar, polar and hydrogen bonding contributions [15]. For ILs, at least two different methods have been used to obtain contributions to *ced*_IP_ (or contributions to Δ_vap_*H*, from which contributions to *ced*_IP_ can be obtained), and these methods are explained in the next two paragraphs. Methods to separate the different contributions to intermolecular interactions, and which contributions get grouped together, can vary widely between different researchers.

Electrostatic contributions are generally those from Coulomb's Law; vdW interactions are usually considered as all other intermolecular interactions (therefore, induction, dispersion and hydrogen bonding are included) [155,196]. Δ_vap_*H* can be separated into electrostatic and vdW contributions [155,156]. To obtain the electrostatic and vdW contributions to *ced*_IP_, the electrostatic and vdW contributions to Δ_vap_*H* simply need to be divided by *V*_m_. To obtain the electrostatic and vdW contributions to *ced*_C+A_, the electrostatic and vdW contributions to Δ_des_*H*(total) (or Δ_vap_*H* + Δ_diss_*H*(CA)) need to be divided by *V*_m_. Such information is available in the literature [155,196].

A second method to separate *ced*_IP_ labels contributions as polar (similar to electrostatic) and non-polar (similar to vdW) [26]. These two contributions are determined by the user defining atoms in the IL that are polar and atoms that are non-polar. Interaction energies for these atoms are then summed to obtain the polar and non-polar contributions to ce_IP_ (when there is an interaction between a polar atom and a non-polar atom, the energy is shared equally). The polar and non-polar contributions to *ced*_IP_ are then obtained by dividing by the partial liquid volumes for the polar and non-polar atoms, respectively.

## 5. Results for Δ_vap_*H*, Δ_des_*H*([C]^+^) and Δ_des_*H*([A] ^−^) for ionic liquids

Δ_vap_*H* for ILs are much larger than Δ_vap_*H* for MLs, as expected. Δ_vap_*H*_298_ values for the ILs investigated to date range from approximately 130 kJ mol^−1^ for [C*_n_*C_1_Im][NTf_2_] (*n* = 1 to 3) to 199 kJ mol^−1^ for [P_6,6,6,14_][BF_4_] (table 1 and electronic supplementary material, table S4). For molecular liquids, Δ_vap_*H*_298_ ranges from 30 kJ mol^−1^ for hexane to 102 kJ mol^−1^ for triethanolamine and 105 kJ mol^−1^ for squalane; for water Δ_vap_*H*_298_ = 44 kJ mol^−1^ [32,33,198].

### 5.1. Δ_vap_*H* from experiments: trends for ionic liquids

**Figure 2.**
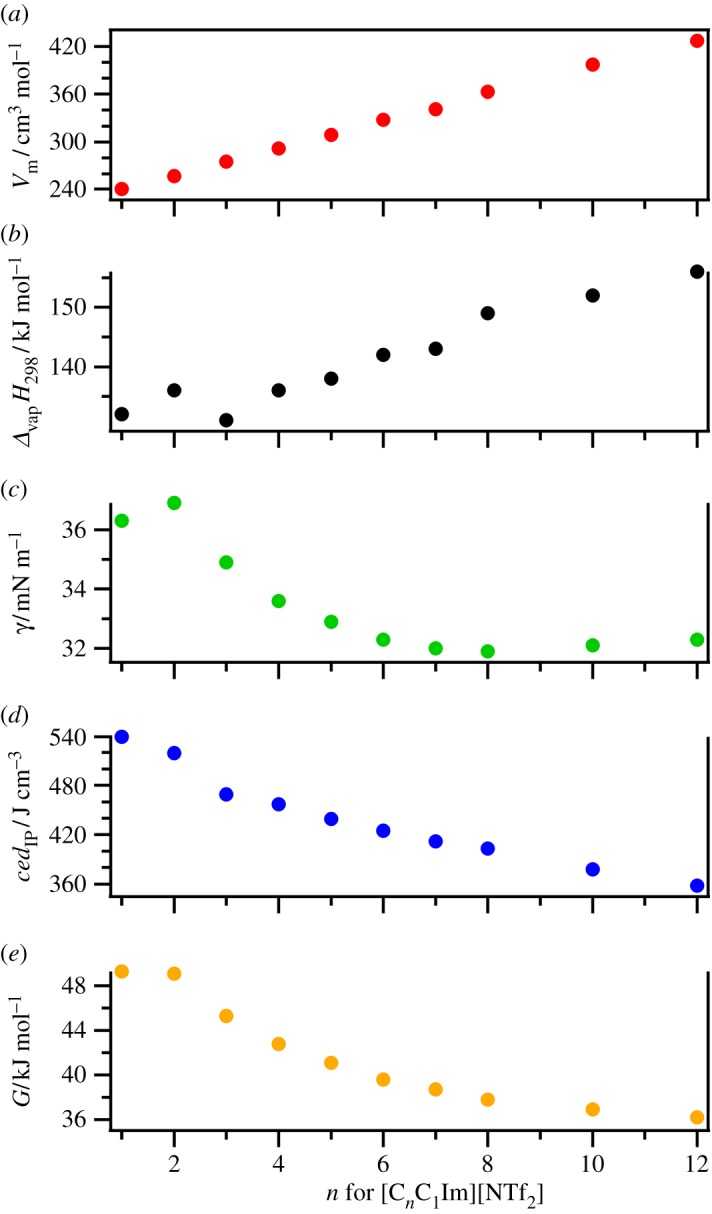
Variation of properties with respect to *n* for [C*_n_*C_1_Im][NTf_2_]: (*a*) molar volume, *V*_m_, (*b*) enthalpy of vaporization at 298 K, Δ_vap_*H*_298_, (*c*) surface tension, *γ*, (*d*) cohesive energy density with an ion pair as the molecular unit, *ced*_IP_ and (*e*) Gordon parameter, *G*. The data used and references are given in table 1. The Δ_vap_*H*_298_ values (and, therefore, the *ced*_IP_ values) were obtained using a constant Δ^g^_l_*C*_p_ = −100 J K^−1^ mol^−1^; it has been discussed elsewhere how using such an approach can lead to non-linearity in Δ_vap_*H*_298_ values for [C*_n_*C_1_Im][NTf_2_] at small *n*, but when variable Δ^g^_l_*C*_p_ values are used the relationship is closer to linear with respect to *n* [111].

For ILs, Δ_vap_*H* values increase as *n* is increased (either on the cation or the anion). This conclusion has been demonstrated for [C*_n_*C_1_Im][NTf_2_] (figure 2*b*) [56,71,108,111,129], [C*_n_*C_1_Im][NPf_2_] [108,118], [C*_n_*C_1_Pyrr][NTf_2_] [73,112], [C*_n_*Py][NTf_2_] [124], [C*_n_*C*_m_*Py][NTf_2_] [134], [C*_n_*C_1_Im][PF_6_] [96], and [C_2_C_1_Im][C*_n_*SO_4_] [57,123]. A broad conclusion for Δ_vap_*H* values is that [NTf_2_] ^−^-based ILs exhibit lower Δ_vap_*H* values than for ILs with anions such as [BF_4_] ^−^ or [N(CN)_2_] ^−^ (when the cation is the same, table 1 and electronic supplementary material, table S4). For ILs with the same anion and different cation cores, there is relatively limited data available. A broad conclusion is that, where *n* is the same, [C*_n_*C_1_Im][NTf_2_] gave lower Δ_vap_*H*_298_ values than [C*_n_*C_1_Pyrr][NTf_2_] and [C*_n_*Py][NTf_2_] (where *n* = 4 or *n* = 6) [56,71,73,108,111,112,124,129].

### 5.2. Δ_Vap_*H* from simulations and calculations

There is a vast amount of Δ_vap_*H*(calc.) data available in the literature, far too much to give a complete summary here. Therefore, the focus here will be on key examples. The most widely used force field for ILs is the CL&P force field [199]. The aim of the developers of this force field was to provide a model that could describe a large range of ILs; hence, only liquid-phase densities and structural data were used for parametrization [199]. Δ_vap_*H*_298_(calc.) using the CL&P force field are in the range of 150 to 250 kJ mol^−1^ [23,156,199]. These Δ_vap_*H*(calc.) are larger than Δ_vap_*H*(exp.), which are in the range of 130 to 180 kJ mol^−1^ for these ILs. This finding is not surprising, given the relatively low levels of parametrization used to produce the CL&P force field. However, the trend of Δ_vap_*H* values increasing as *n* increases is found, and [C_4_C_1_Im][NTf_2_] giving a small Δ_vap_*H*_298_(calc.) relative to other [C_4_C_1_Im][A] ILs, suggests that correct trends in intermolecular interactions are captured. Köddermann *et al.* [154,155] parametrized a force field against experimental data for [C_2_C_1_Im][NTf_2_], including structural, dynamic and thermodynamic data (liquid-phase density, self-diffusion coefficients of cations and anions and NMR rotational correlation times for cations). Δ_vap_*H*_298_(calc.) for [C*_n_*C_1_Im][NTf_2_] (*n* = 2, 4, 6, 8) using this force field matched very well to Δ_vap_*H*_298_(exp.) [56,71,154], demonstrating that force fields can be produced for ILs that do an excellent job of capturing the intermolecular interactions. Schröder and Coutinho used the continuum solvation model COSMO–RS for a structurally diverse set of ILs to obtain Δ_vap_*H*_298_(calc.) [158]. Δ_vap_*H*_298_(calc.) matched very well to Δ_vap_*H*_298_(exp.) (±10 kJ mol^−1^), demonstrating that the continuum solvation model captured the bulk liquid very well.

### 5.3. Δ_des_*H*([C]^+^), Δ_des_*H*([A] ^−^) and Δ_diss_*H*(CA) from experiments, simulations and calculations

Δ_des_*H*([C]^+^) and Δ_des_*H*([A] ^−^) have been experimentally measured for [C_2_C_1_Im][NTf_2_] only. Therefore, *ced*_C+A_ can only be obtained from experimental values for [C_2_C_1_Im][NTf_2_]. Δ_des_*H*_490_([C]^+^) = 206 kJ mol^−1^ and Δ_des_*H*_490_([A] ^−^) = 207 kJ mol^−1^ at *T* ∼ 490 K. Therefore, Δ_des_*H*_490_([C]^+^) +Δ_des_*H*_490_([A] ^−^) =Δ_des_*H*_490_(total) = 413 kJ mol^−1^. Using Δ_des_*H*_490_(total) (exp.) = 413 kJ mol^−1^ and Δ_vap_*H*_490_(exp.) = 117 kJ mol^−1^ (constant Δ^g^_l_*C*_p_) an experimentally derived value of Δ_diss_*H*_490_(CA) (exp.) = 296 kJ mol^−1^ can be obtained for the first time.

For [C_2_C_1_Im][NTf_2_] Δ_des_*H*_490_(total) (exp.) = 413 kJ mol^−1^ (table 3) is approximately 3½ times larger than Δ_vap_*H*_490_(exp.) = 117 kJ mol^−1^ (constant Δ^g^_l_*C*_p_ = −100 J K^−1^ mol^−1^). It takes 3½ times more energy to vaporize two isolated ions than to vaporize a neutral ion pair.

**Table 3.** Δ_vap_*H*_490_ was extrapolated using constant Δ^g^_l_*C*_p_ = −100 J K^−1^ mol^−1^. Δ_des_*H*_490_([C]^+^) and Δ_des_*H*_490_([A] ^−^) are taken from Dunaev *et al.* [91]. Δ_diss_*H*_490_(CA) (exp.) = Δ_des_*H*_490_(total) − Δ_vap_*H*_490_. *ced*_C+A,490_(exp.) has been rounded to three significant figures.

ionic liquid	Δ_vap_*H*_490_ (exp.) kJ mol^−1^	Δ_des_*H*_490_([C]^+^) (exp.) kJ mol^−1^	Δ_des_*H*_490_([A] ^−^) (exp.) kJ mol^−1^	Δ_des_*H*_490_(total) (exp.) kJ mol^−1^	Δ_diss_*H*_490_(CA) (exp.) kJ mol^−1^	*ced*_C+A,490_ (exp.) J cm^−3^
[C_2_C_1_Im][NTf_2_]	117	207	206	413	296	1580

Calculated Δ_diss_*H*_298_(CA) for [C*_n_*C_1_Im][A] ILs gives 340 to 430 kJ mol^−1^ at 298 K (table 2) [103]. Δ_diss_*H*(CA) varies very little with increasing *n* for the high-level calculations in [103,196], whereas for the lower-level calculations in [104] Δ_diss_*H*(CA) decreases with increasing *n* (which is a very surprising finding, given one would expect both the electrostatic and vdW interactions for a neutral ion pair to be relatively unaffected by alkyl chain length). A combination of these Δ_diss_*H*_298_(CA) (calc.) values from [103] and Δ_vap_*H*_298_(exp.) gave 480 kJ mol^−1^ < Δ_des_*H*_298_(total) (calc.) < 540 kJ mol^−1^ (table 2).

### 5.4. Separating Δ_vap_*H*, Δ_diss_*H*(CA), Δ_des_*H*([C]^+^) and Δ_des_*H*([A] ^−^) into electrostatic and vdW contributions

The electrostatic and vdW contributions to Δ_vap_*H* obtained from molecular dynamics simulations of the liquid phase vary greatly for different ILs (and often give overestimates of the experimental Δ_vap_*H* values, §5.2). The electrostatic contributions range from 60 to 200 kJ mol^−1^, depending on the IL; the vdW contributions range from 70 to 210 kJ mol^−1^ [156]. For example, for [C_8_C_1_Im][NTf_2_] the electrostatic and vdW contributions to Δ_vap_*H* are 73 and 114 kJ mol^−1^ respectively; in contrast, for [C_4_C_1_Im][BF_4_] the electrostatic and vdW contributions to Δ_vap_*H* are 117 and 64 kJ mol^−1^, respectively [156].

Using molecular dynamics simulations for [C*_n_*C_1_Im][NTf_2_] it has been found that as *n* increases the electrostatic contribution stays approximately constant while the vdW contribution increases significantly [155,156]; the same has been found for [C*_n_*C_1_Im][NO_3_] and [C*_n_*C_1_Im][PF_6_] [26,156]. Therefore, the increase in Δ_vap_*H* with increasing *n* is due to extra vdW contributions. For the IL [P_6,6,6,14_][NTf_2_], which contains 28 CH_2_ groups and 4 CH_3_ groups, the electrostatic and vdW contributions to Δ_vap_*H* are 67 and 200 kJ mol^−1^, respectively, demonstrating that vdW contributions can dominate for certain ILs [156].

For [C_4_C_1_Im][A] ILs the vdW contributions are all in the range of 60 to 100 kJ mol^−1^, whereas the electrostatic contributions have a far larger range, from 80 to 210 kJ mol^−1^. [C_4_C_1_Im][NTf_2_] has a far smaller electrostatic contribution than [C_4_C_1_Im][BF_4_]. These findings demonstrate that [C_4_C_1_Im][BF_4_] has a larger Δ_vap_*H* than [C_4_C_1_Im][NTf_2_] due to stronger electrostatic contributions.

Electrostatic intermolecular interaction contributions dominate Δ_diss_*H*(CA), with vdW contributions near zero, for the ILs studied ([C*_n_*C_1_Im][A] and [C*_n_*C_1_Pyrr][A] with a wide range of anions) [155,196]. For example, for [C_2_C_1_Im][NTf_2_] the electrostatic contribution to Δ_diss_*H*(CA) is actually larger than Δ_diss_*H*(CA) itself, i.e. the vdW interactions are stronger in the isolated [C]^+^ and isolated [A] ^−^ than in the CA neutral ion pair [196]. Given that Δ_diss_*H*(CA) are always significantly larger than Δ_vap_*H*, this finding shows that electrostatic contributions dominate Δ_des_*H*.

## 6. Cohesive energy densities: results

As noted in §4.4.5, there are many Δ_vap_*H* values available in the literature for ILs. Unsurprisingly, some of these Δ_vap_*H* values have been used to obtain *ced*_IP_, e.g. in [200–203]. A key difference is that in this article the Δ_vap_*H* data used is rigorously selected; only Δ_vap_*H* values obtained directly from heating experiments are included, and where doubts exist over the Δ_vap_*H* values they are not included (as outlined in §4.4.3).

### 6.1. Trends in *ced*_IP_ for ionic liquids

**Figure 3.**
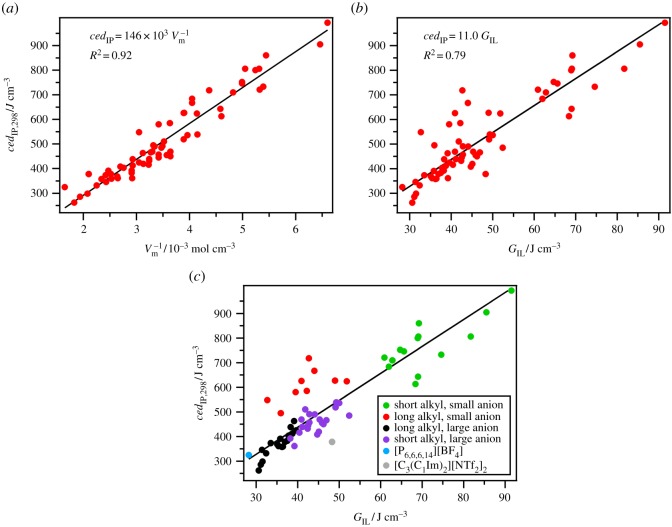
*ced*_IP,298_ for ILs (extrapolated to *T* = 298 K using constant Δ^g^_l_*C*_p_ = −100 J K^−1^ mol^−1^) versus: (*a*) $V_{m}^{-1}$, (*b*) *G*_IL_, (*c*) *G*_IL_. The same data are plotted in (*b*) and (*c*); in (*c*) different IL families are plotted as the same colour data points. Details on the families are given in §7.1; [P_6,6,6,14_][BF_4_] and the dicationic IL [C_3_(C_1_Im)_2_][NTf_2_]_2_ do not fit into any of the four categories used here.

*ced*_IP,298_ values range from 262 J cm^−3^ for [C_10_C_10_Im][NTf_2_] to 9932 J cm^−3^ for [C_2_C_1_Im][SCN] (table 1). The range for Δ_vap_*H*_298_ is approximately 130 kJ mol^−1^ to approximately 200 kJ mol^−1^. The range for *V*_m_ is approximately 150 cm^3^ mol^−1^ to approximately 600 cm^3^ mol^−1^. The range for *ced*_IP,298_ is clearly larger than for Δ_vap_*H*_298_, caused by the larger range for *V*_m_ over Δ_vap_*H*_298_ (table 1). A plot of *ced*_IP,298_ against $V_{m}^{-1}$ (figure 3*a*) shows a very good linear correlation (*R*^2^ = 0.92). Marcus recently found a similar correlation between *ced*_IP_ (using Δ_vap_*H* both from experimental and indirect measurements) and the ionic volume (which is proportional to *V*_m_) [202]. This finding demonstrates conclusively that the Δ_vap_*H*_298_ values are not the key factor in determining *ced*_IP_ for ILs; the key factor is *V*_m_, i.e. IL size. Large ILs have small *ced*_IP_, and small ILs have large *ced*_IP_; size matters. *ced*_IP_ can now be predicted with good reliability from calculations alone with no experimental input, as you just need to know *V*_m_ (or *V*_mol_), and *V*_m_ can be predicted without IL synthesis [195,204].

### 6.2. *ced*_c+A_ for ionic liquids

Despite Δ_des_*H*([C]^+^) and Δ_des_*H*([A] ^−^) being experimentally measured for an IL, *ced*_C+A_ has not been obtained from the experimental data to date, as far as I am aware. For [C_2_C_1_Im][NTf_2_] experimentally derived *ced*_C+A,490_ = 1580 J cm^−3^ (rounded to three significant figures) at approximately 490 K.

*ced*_C+A,298_ obtained from a combination of experimental (Δ_vap_*H*_298_, table 1) and calculated (Δ_diss_*H*_298_(CA)) results gives approximately 1600 J cm^−3^ < *ced*_C+A,298_ < approximately 3400 J cm^−3^. *ced*_C+A,298_ obtained solely from calculations for [C*_n_*C_1_Im][BF_4_] and [C*_n_*C_1_Im][PF_6_] gave approximately 2000 J cm^−3^ to approximately 3200 J cm^−3^ [104].

*ced*_C+A,298_ obtained from a combination of experimental (Δ_vap_*H*_298_, table 1) and calculated (Δ_diss_*H*_298_(CA)) [103] results gives approximately 1850 J cm^−3^ for [C_2_C_1_Im][NTf_2_]. Given the potential for errors from both experiments (the signals to measure Δ_des_*H*([C]^+^) and Δ_des_*H*([A] ^−^) in particular are very small and this measurement has only been made for one IL to date) and calculations (they have not been validated against experimental data) the match between the experimentally derived *ced*_C+A,490_ = 1580 J cm^−3^ and the value above *ced*_C+A,298_ = 1850 J cm^−3^ is good (and the *T* values are also different). This match gives confidence that the *ced*_C+A,298_ values derived from a combination of experimental and calculated data give a reasonable measure of the IL total intermolecular interactions; certainly comparisons between two *ced*_C+A,298_ values can be trusted when the difference between the two *ced*_C+A,298_ values is large.

### 6.3. Separating *ced* into electrostatic and vdW contributions

There is currently little data in the literature from MD simulations for electrostatic and vdW contributions to *ced*_IP_. Data needed to obtain electrostatic and vdW contributions to *ced*_IP_ is available [155,156], but the simple calculations have not been carried out; such data are presented in table 4. As explained in §5.2, the Δ_vap_*H*_298_(calc) values in [156], and, therefore, *ced*_IP_, are overestimates of the Δ_vap_*H*_298_(exp.) values; it is expected that the vdW contributions are too large [156]. The electrostatic contributions to *ced*_IP,298_ are between 90 and 620 J cm^−3^, whereas the vdW contributions to *ced*_IP,298_ are between 160 and 350 J cm^−3^.

**Table 4.** Separating *ced* into electrostatic and vdW contributions using a combination of simulations and calculations, along with experimental molar volume, *V*_m_. The references given are for the simulation/calculation literature.

ionic liquid	Δ_vap_*U* (vdW) kJ mol^−1^	Δ_vap_*U* (elec) kJ mol^−1^	*V*_m_ (exp.) cm^3^ mol^−1^	*ced*_IP_ (exp.) J cm^−3^	*ced*_IP_ (vdW) J cm^−3^	*ced*_IP_ (elec) J cm^−3^	*δ*_H,IP_ J cm^−3^	*δ*_H,IP_ (vdW) J cm^−3^	*δ*_H,IP_ (elec) J cm^−3^	ref.
[C_2_C_1_Im][NTf_2_]	42	82	258	481	162	318	21.9	12.7	17.8	[155]
[C_4_C_1_Im][NTf_2_]	50	78	292	438	171	268	20.9	13.1	16.4	[155]
[C_6_C_1_Im][NTf_2_]	60	78	328	421	182	237	20.5	13.5	15.4	[155]
[C_8_C_1_Im][NTf_2_]	71	79	363	413	195	217	20.3	14.0	14.7	[155]
[C_2_C_1_Im][NTf_2_]	89	81	258	659	345	314	25.7	18.6	17.7	[156]
[C_4_C_1_Im][NTf_2_]	99	79	292	610	339	270	24.7	18.4	16.4	[156]
[C_6_C_1_Im][NTf_2_]	108	76	328	561	329	232	23.7	18.1	15.2	[156]
[C_8_C_1_Im][NTf_2_]	114	73	363	515	314	201	22.7	17.7	14.2	[156]
[P_6,6,6,14_][NTf_2_]	200	67	728	367	275	92	19.2	16.6	9.6	[156]
[C_4_C_1_Im][PF_6_]	68	116	207	889	329	560	29.8	18.1	23.7	[156]
[C_4_C_1_Im][BF_4_]	64	117	188	963	340	622	31.0	18.4	24.9	[156]

For [C*_n_*C_1_Im][NO_3_] (*n* = 2, 4, 6, 8) the polar contributions to *ced*_IP,298_ are between 1300 and 1400 J cm^−3^ for all four ILs, whereas the non-polar contribution to *ced*_IP,298_ are between 290 and 400 J cm^−3^ for all four ILs [26]. These values are not comparable to the values given in the previous paragraph, as their origins are very different (see §4.5).

Using data from [155] and [196] for [C_2_C_1_Im][NTf_2_], Δ_des_*H*_298_(elec) = 82 + 358 = 440 kJ mol^−1^ and Δ_des_*H*_298_(vdW) = 42 – 16 = 26 kJ mol^−1^. Therefore, the electrostatic contribution to *ced*_C+A,298_ for [C_2_C_1_Im][NTf_2_] is 1710 J cm^−3^, whereas the vdW contribution to *ced*_C+A,298_ is 100 J cm^−3^. The electrostatic contribution dominates the total intermolecular interaction for [C_2_C_1_Im][NTf_2_]. Such dominance is expected to hold for other ILs; compared to [C_2_C_1_Im][NTf_2_], the vdW contribution is expected to be even less important for [C_2_C_1_Im][SCN], but more important for [C_8_C_1_Im][NTf_2_].

## 7. Can cohesive energy densities be used to understand other properties?

A key question to answer is: are either of the two measures of IL intermolecular interactions, *ced*_IP_ and *ced*_C+A_, useful for understanding IL liquid-phase properties? The only way to answer that question is through obtaining data and developing trends using *ced*_IP_ and *ced*_C+A_. At this stage only *ced*_IP_ can be used to understand other properties; there are insufficient experimental *ced*_C+A_ values to make meaningful comparisons and develop trends.

### 7.1. Gordon parameter, *G*

A range of methods have been used to measure *γ* for ILs [205]. There is a large variation in *γ* values for the same IL across different publications [205]. This occurrence is most likely due to impurities present in the sample. Many IL *γ* values are measured under conditions where water and other volatile impurities can contaminate the sample; in addition, grease-type impurities have been identified at IL–gas surfaces [206]. These contaminants are likely to affect *γ*.

ILs have *γ* values in the range of 26 to 60 mN m^−1^ [181,205,207,208]. Molecular liquids have *γ* values in the range of 18 to 72 mN m^−1^ [209–211]. Therefore, ILs and molecular liquids have *γ* values of the same magnitude (unlike Δ_vap_*H*_298_). For ILs, *γ* values decrease as *n* is increased [205]. It is more difficult to draw conclusions on the effect of the anion on IL *γ* values, due to both anion complexity and variations in *γ* values across different publications. However, a broad conclusion is that [NTf_2_] ^−^-based ILs exhibit lower *γ* values than for ILs with anions such as [BF_4_] ^−^ or [N(CN)_2_] ^−^ (for the same cation) [205].

*γ* and Δ_vap_*H* are not linearly correlated. This observation is most readily highlighted by considering the effect of increasing the alkyl chain length, *n*; Δ_vap_*H* increased and surface tension decreased (e.g. for [C*_n_*C_1_Im][NTf_2_], figure 2*b,c*). Most importantly, this observation clearly demonstrates that *γ* and Δ_vap_*H* should not be used for quantfying the strength of intermolecular interactions of ILs; comparisons need to be made for values in the same units.

*G* values range from 28.2 J cm^−3^ for [P_6,6,6,14_][BF_4_] to 91.5 J cm^−3^ for [C_2_C_1_Im][SCN] (for those ILs for which experimental Δ_vap_*H* values are also available, table 1). *G* for molecular liquids range from 29 J cm^−3^ for squalane, 48 J cm^−3^ for ethanol, 128 J cm^−3^ for glycerol and 231 J cm^−3^ for water (electronic supplementary material, table S5).

There is a good linear correlation between *ced*_IP_ and *G* for ILs (figure 3*b*). Marcus recently found a similar correlation between *ced*_IP_ (using Δ_vap_*H* both from experimental and indirect measurements) and *G* [212]. This observation strongly suggests that *ced*_IP_ and *G* both capture the same intermolecular interactions for ILs. *G* shows a good linear correlation with the inverse of size, $V_{m}^{-1}$, i.e. a small IL has a large *G* value (electronic supplementary material, figure S2). This is as expected, given the correlations given previously, and reiterates that the strength of intermolecular interactions in ILs can be obtained, to a good degree of accuracy, from their size.

**Figure 4.**
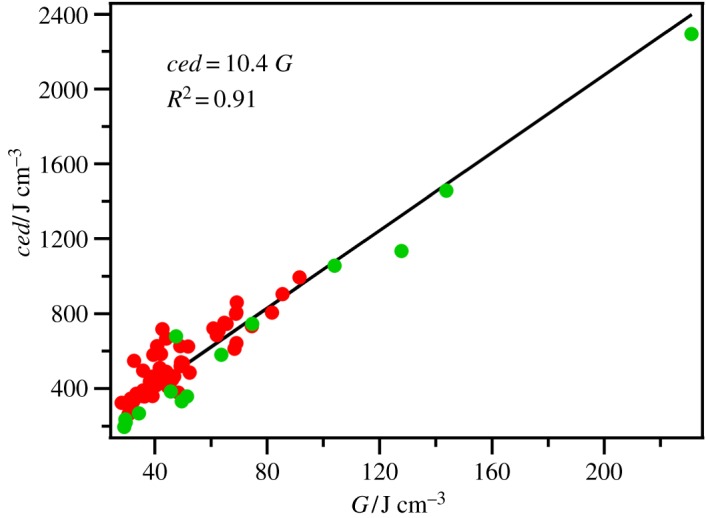
*ced* (including both *ced*_ML_ and *ced*_IP_) versus *G* (including *G* for ILs and *G* for a selection of molecular liquids).

There is a good linear correlation between *ced* and *G* when both molecular and ILs are considered (figure 4). This relationship strongly suggests that there is an underlying relationship between *ced* and *G* that is not dominated by the ionic nature of ILs.

Although a major aim of this article is to find correlations that hold across all ILs, it is important to compare *ced*_IP_ and *G*_IL_ for select IL families/categories. Categorizing the ILs in figure 3 into different families is potentially arbitrary. ILs with a large anion [75,195] ([NTf_2_] ^−^, [NPf_2_] ^−^ and [FAP] ^−^) are grouped together here. It must be noted that [C_4_SO_4_] ^−^ and [C_8_SO_4_] ^−^ are relatively large, but this is caused by the large alkyl chain, and hence these anions are classified here as small (the reasons for this classification will become clear shortly). ILs with small anions are categorized here as all those that do not contain [NTf_2_] ^−^, [NPf_2_] ^−^ or [FAP] ^−^.

For ILs with a large anion and any length alkyl chain both *ced*_IP_ and *G*_IL_ were relatively small (figure 3*c*), as all data points are located in the bottom left of figure 3*c*. In addition, all of these ILs with large anions match relatively well to the line of best fit for all ILs, first presented in figure 3*b*. Within that family, ILs with long alkyl chains (defined here as having at least one alkyl chain of length C_6_H_13_, i.e. [C_6_C_1_Im][NTf_2_], although ILs with two intermediate alkyl chains were also included in this category, i.e. [C_4_C_4_Im][NTf_2_]) are coloured black (figure 3*c*), and ILs with short alkyl chains are coloured purple (figure 3*c*). Clearly, ILs with long alkyl chains gave smaller *ced*_IP_ and *G*_IL_ than those with long short alkyl chains.

ILs with small anions and short alkyl chains (plotted as green points on figure 3*c*) gave the largest *ced*_IP_ and *G*_IL_. Therefore, to obtain an IL with the combination of both a large *ced*_IP_ and a large *G*_IL_ a short alkyl chain and a small anion is required. ILs with small anions and long alkyl chains (plotted as red points on figure 3*c*) gave relatively large *ced*_IP_ but smaller than expected *G*_IL_, based upon their *ced*_IP_. While conclusions based on individual data points should be treated with caution, the presence of a cluster of red data points (figure 3*c*) suggests that the relationship for ILs with small anions and long alkyl chains is different to other ILs reported here. One possible explanation for this observation for ILs with small anions and long alkyl chains is the influence of the IL–gas surface structure on *G*_IL_. There is a large amount of literature showing that ILs tend to orient with their longer alkyl chains, on average, at the outer IL–gas surface, with the rest of the IL, on average, located just below the outer IL–gas surface [213,214]. Intermolecular interactions for alkyl chains are weaker than those for the other parts of the ILs (e.g. ion–ion electrostatic intermolecular interactions). Therefore, having more alkyl chains located near the outer IL–gas surface than expected based on the stoichiometry will lead to lower *G*_IL_ but will have no effect on *ced*_IP_. At this stage, there is insufficient evidence to draw this conclusion with high confidence, but there is a growing amount of evidence to support it.

### 7.2. Ionic liquids as solvents for self-assembly

In 2013 a summary was presented of aprotic ILs that support amphiphile self-assembly; at least 11 aprotic ILs were found, in addition to at least 37 protic ILs [40]. ILs that promote self-assembly for which *ced*_IP_ and *G* have also been measured are listed in table 5. Clearly, the rule [13,14] given by Evans of self-assembly only occurring in solvents with *G* > 110 J cm^−3^ does not hold. The lower limit from this data would appear to be *G*_IL_ > 42.8 J cm^−3^ and *ced*_IP_ > 457 J cm^−3^. This would suggest that any IL with *G*_IL_ and *ced*_IP_ greater than these values would be able to support self-assembly. ILs that meet these criteria include [C_6_C_1_Im][PF_6_] and [C_8_C_1_Im][N(CN)_2_]. Certain ILs meet the *ced*_IP_ rule but not the *G* rule, e.g. [C_8_C_1_Im]Cl and [C_4_C_1_Im][C_8_SO_4_]. This lack of agreement between *ced*_IP_ and *G*_IL_ may well be due to the influence of the IL–gas surface structure, as outlined in §7.1. Therefore, *ced*_IP_ would appear not to be the best guide for determining self-assembly in ILs; *G*_IL_ is a much better guide for determining whether an IL can support self-assembly.

**Table 5.** Aprotic ILs which are known to support amphiphile self-assembly [40] and for which *ced*_IP_ and *G* have also been measured.

ionic liquid	*ced*_IP,298_ / J cm^−3^	*G*_IL_/J cm^−3^
[C_4_C_1_Im][NTf_2_]	457	42.8
[C_2_C_1_Im][NTf_2_]	519	49.1
[C_4_C_1_Im][PF_6_]	710	62.8
[C_2_C_1_Im][C_2_SO_4_]	800	68.9
[C_4_C_1_Im][BF_4_]	806	69.1
[C_4_C_1_Pyrr][N(CN)_2_]	806	81.7

### 7.3. Solubility

*δ*_H,indirect_ for ILs have been obtained from activity coefficients at infinite dilution, giving 19 J^1/2^ cm^−3/2^ < *δ*_H,indirect_ < 30 J^1/2^ cm^−3/2^ [50,200,201,215–218] and, therefore, 350 J cm^−3^ < *ced*_indirect_ < 900 J cm^−3^. At present, comparisons of *ced*_IP_ and *ced*_indirect_ obtained from measurements of *δ*_H_ for ILs are difficult, as there is insufficient data to draw conclusions across a sufficiently large dataset. Marcus has compared *δ*_H,IP_ for ILs (obtained from *ced*_IP_ data) with the solubility of liquid organic solutes in ILs [203]. The *ced*_IP_ data (and therefore, *δ*_H,IP_ for ILs) were obtained from both experimental Δ_vap_*H* values and Δ_vap_*H* from less reliable indirect/calculation methods. However, as outlined in §6.1, there is an excellent correlation of *ced*_IP_ and $V_{m}^{-1}$, so less reliable Δ_vap_*H* values are unlikely to have a large effect on *ced*_IP_ values. Therefore, the findings in this paper are expected to hold if only experimental Δ_vap_*H* values were used to obtain *ced*_IP_. Overall, no satisfactory correlation was found, demonstrating that the theories that underpin the use of *δ*_H_ for predicting solubilities do not hold for ILs, at least for organic solutes.

Hansen solubility parameters have been obtained for a range of ILs using indirect methods [219–222]. These values could potentially be compared to *δ*_H,IP_(vdW) and *δ*_H,IP_(elec) (obtained from *ced*_IP_(vdW) and *ced*_IP_(elec) respectively), examples of which are given in table 4, even though the categories are different. However, at this stage there is insufficient data both in terms of Hansen solubility parameters from indirect measurement and *ced*_IP_(vdW) and *ced*_IP_(elec) to make satisfactory comparisons.

### 7.4. Viscosity

*ced* values for ILs have been estimated from viscosity measurements of ILs and IL-based solutions [166,221,223–227]. These give values in the range 600 J cm^−3^ < *ced*_indirect_ < 900 J cm^−3^. At present, as for data from measurements of solubility, there is an insufficiently large dataset to draw definitive conclusions on any correlations with *ced*_IP_. Very recently, a linear correlation has been presented between *ce* and *E*_a,vis_ for three protic ILs [228]. *C* = 5 was found, for *ce*_IP_ = *C · E*_a,vis_. The Δ_vap_*H* values used to obtain this correlation were not included in correlations in this article, for reasons outlined in §4.4.3. It is assumed that, at temperatures at which the vaporization measurements were made, the unit of viscous flow in the liquid phase was ion pairs. In such a situation, using data based on Δ_vap_*H* and not Δ_des_*H*(total) makes sense.

### 7.5. Conclusions of *ced*_IP_ versus *ced*_indirect_

Indirect methods of obtaining *ced*_indirect_ for ILs (i.e. intrinsic viscosity or solubility data) have so far proved unsuccessful in matching *ced*_IP_ data. There are a number of possible reasons for this finding: insufficient/poor quality data (ILs are known for containing impurities), entropy may be vital (especially for solubility), ILs are large and only need to break select intermolecular interactions for processes such as solvation or new surface formation to occur, and the importance of non-polar interactions in ILs (non-polar interactions are known to cause problems for *δ*_H_ [15,49]). These last reasons would suggest that an approach similar to the Hansen solubility parameter approach might prove useful, if reliable data can be obtained.

## 8. Insights from cohesive energy densities of ionic liquids

### 8.1. Ionic liquids have very large total intermolecular interactions

As explained in §2.2 and §4.3, *ced*_ML_ and *ced*_C+A_ (but not *ced*_IP_) represent the total intermolecular interactions, i.e. the total cohesion in a unit volume. *ced*_ML,298_ values range from 195 J cm^−3^ for squalane, through 385 J cm^−3^ for acetone, 679 J cm^−3^ for ethanol and finishing with 2293 J cm^−3^ for water [32]. For [C_2_C_1_Im][NTf_2_] experimentally derived *ced*_C+A_ = 1576 J cm^−3^ at approximately 490 K. Clearly, [C_2_C_1_Im][NTf_2_] has stronger intermolecular interactions than most molecular liquids. Furthermore, based upon computationally derived *ced*_C+A_ values, some ILs have stronger intermolecular interactions than water. However, ILs certainly have significantly weaker intermolecular interactions than classical molten salts; Marcus has estimated *ced*_C+A_ for molten salts ranging from 6241 for CsI to 64 520 J cm^−3^ for LiF [229]. Based upon the values for both *ced*_IP_ and *ced*_C+A_ presented here, ILs have very strong intermolecular interactions (many larger than water). Calculations and simulations indicated that these very strong intermolecular interactions are because ILs are ionic (§6.3); overcoming the electrostatic interactions to break charge neutrality costs a great deal of enthalpy per ion pair.

### 8.2. Intermolecular interactions for ions in the liquid phase versus ions in the vapour phase

A very simplistic, but potentially insightful, model can be developed for [C_2_C_1_Im][NTf_2_], given the large amount of data available for this IL. Liquid-phase molecular dynamics simulations for [C_2_C_1_Im][NTf_2_] gives the number of neighbouring cations surrounding an anion as approximately seven [230]. Therefore, Δ_des_*H*_490_(total) = 413 kJ mol^−1^ can be viewed as the enthalpy to break 14 cation–anion intermolecular interactions (as seven cation–anion intermolecular interactions need to be broken for the cation and seven for the anion). Consequently, each cation–anion intermolecular interaction is approximately 30 kJ mol^−1^. Δ_diss_*H*_490_(CA) = 296 kJ mol^−1^ is the enthalpy to break one cation–anion intermolecular interaction in the vapour phase. Very clearly, the vapour phase CA intermolecular interaction is substantially stronger than the average liquid-phase CA intermolecular interaction. This stronger vapour phase intermolecular interaction is most likely because having just one cation and one anion, with no other electrostatic intermolecular interactions that might weaken this one interaction, would lead to a particularly strong single electrostatic CA intermolecular interaction.

Given the strength of the liquid-phase total intermolecular interactions for ILs, Δ_vap_*H* values are actually smaller than one might expect. The reason for this is that vapour phase cation–anion intermolecular interactions are very strong.

### 8.3. Ionic liquids are associated liquids

*r*(*ced*_IP_) and *r*(*ced*_C+A_) are the ratios between *P*_int_ and *ced*_IP_ and *ced*_C+A_, respectively. For ILs, 0.60 < *r*(*ced*_IP_) < 0.85 when *ced*_IP_ values are used, e.g. for [C_2_C_1_Im][NTf_2_] *r*(*ced*_IP_) = 0.81. Therefore, *ced*_IP_ is considerably larger than *P*_int_ for all ILs [54]. These *r*(*ced*_IP_) values are similar to what are collectively termed associated/tight/stiff liquids, e.g. acetone (*r* = 0.67), *n*-hexanol (*r* = 0.65) [54]. For [C_2_C_1_Im][NTf_2_] *ced*_C+A,490_ = 1580 J cm^−3^ and *P*_int_ = 392 J cm^−3^ [54,231]; therefore, *r*(*ced*_C+A_) = 0.25. This *r*(*ced*_C+A_) value still leads to categorization of [C_2_C_1_Im][NTf_2_] as an associated liquid, but puts [C_2_C_1_Im][NTf_2_] in the region of methanol (*r* = 0.33) and formamide (*r* = 0.36); *r* = 0.07 for water, *r* = 0.24 ± 0.02 for liquid metals [232] and *r* < 0.10 for molten salts [232]. Overall, ILs would be classified as having stronger attractive intermolecular interactions than fluorocarbons, but weaker attractive intermolecular interactions than liquid metals, molten salts and water.

### 8.4. The importance of charge neutrality

*ced*_C+A_ values are much larger than *ced*_IP_ values (tables 2 and 3). *V*_m_ used to calculate both *ced*_IP_ and *ced*_C+A_ are obviously the same, so the very large difference is caused by the intermolecular interactions that are broken for vaporization of neutral ion pairs and isolated ions, respectively.

Values obtained from both intrinsic viscosity and solubility measurements are in the range of 350 J cm^−3^ < *ced*_indirect_ <  J cm^−3^. These values match the magnitude of the experimental values for *ced*_IP_ (250 J cm^−3^ < *ced*_IP_ < 1000 J cm^−3^) and are very different from both the experimental value for *ced*_C+A_ and also the *ced*_C+A_ values obtained via indirect methods (1550 J cm^−3^ < *ced*_C+A_ < 3400 J cm^−3^). In addition, there is the good linear correlation between *ced* and *G* for a dataset including both ILs and molecular liquids, where *ced*_IP_ is used for ILs. All of this evidence indicates that the intermolecular interactions that are broken for ILs when measuring *ced*_IP_ are similar to those broken for ILs when measuring *ced*_indirect_.

A possible rationalization for the above observations centres on maintaining charge neutrality. When breaking intermolecular interactions to either form holes in a liquid or to form a new IL–gas surface, charge neutrality will be maintained, i.e. all ions will always have at least two close-contact neighbours [230], and no ion will be left without a counterion. In essence, in the liquid phase, charge neutrality is always maintained. For IL vaporization the ionic vapour is composed almost exclusively of neutral ion pairs; therefore, charge neutrality is also maintained, as every ion has a counterion present. Therefore, the physical processes that underpin *ced*_indirect_, *G* and *ced*_IP_ all maintain charge neutrality. However, for ion desorption all intermolecular interactions are broken; therefore, the processes that underpin *ced*_C+A_ break charge neutrality. Therefore, it is possible that *ced*_IP_—rather than *ced*_C+A_—is the key value for understanding other properties that are underpinned by the strength of IL intermolecular interactions, even though *ced*_IP_ is not a measure of the total IL intermolecular interactions.

### 8.5. Why are ionic liquids involatile compared to molecular liquids?

A key question is: why are ILs so involatile compared to molecular liquids? Is it because ILs have very strong electrostatic intermolecular interactions in the liquid phase? *ced*_IP_ is a measure of the strength of intermolecular interactions that control IL vaporization. There are both liquid-phase and vapour-phase intermolecular interactions that contribute to Δ_vap_*H* and therefore, *ced*_IP_ (see equation (8.1), which is simply equation (4.4) rearranged).

8.1 $$\Delta_{\mathrm{vap}}H=\Delta_{\mathrm{des}}H(\text{total})-\Delta_{\mathrm{diss}}H(\text{CA}).$$

For ILs, electrostatic intermolecular interactions dominate the total intermolecular interactions in the liquid phase, relative to the vdW intermolecular interactions (i.e. electrostatic intermolecular interactions control Δ_des_*H*, see §6.3). However, contributions of electrostatic intermolecular interactions are relatively large for the vapour phase of ILs (i.e. for Δ_diss_*H*). Consequently, the contribution of electrostatic intermolecular interactions to Δ_vap_*H* for ILs is not dominant; as the liquid and vapour phase electrostatic contributions are similar in magnitude they subtract (see equation (8.1)) to give a relatively small electrostatic contribution to Δ_vap_*H*. Certain ILs have electrostatic contributions to Δ_vap_*H* of approximately 60 kJ mol^−1^ (see §5.4), which is significantly less than Δ_vap_*H* for certain molecular liquids, e.g. Δ_vap_*H* = 102 kJ mol^−1^ for triethanolamine [32]. Hence, it can be concluded that electrostatic intermolecular interactions are not the dominant factor controlling the large Δ_vap_*H* of ILs (relative to molecular liquids); vdW intermolecular interactions are important. These observations contradict the theory that the low volatility of ILs is due to their ionic nature, i.e. their strong electrostatic intermolecular interactions [24–27].

Having established that vdW intermolecular interactions matter for ILs for controlling Δ_vap_*H*, it is important to understand what determines the contribution of these vdW intermolecular interactions. For the intermolecular interaction vdW contributions, size matters. The vdW contributions to Δ_vap_*H* for many, if not all, ILs are expected to be significantly larger than Δ_vap_*H* for water. [C_8_C_1_Im][NTf_2_] is far larger than water, so for [C_8_C_1_Im][NTf_2_] the vdW contribution to Δ_vap_*H* is much larger than for water. The relatively large vdW contributions in ILs are not due to stronger vdW intermolecular interactions; there are simply more such vdW intermolecular interactions for ILs, as ILs are significantly larger than most molecular liquids. It is the same argument as to why certain hydrocarbons, e.g. squalene, have relatively large Δ_vap_*H* but small *ced*_ML_ (see §2.3) [33].

Clearly, ILs are less volatile than molecular liquids. However, ILs can actually be considered more volatile than their liquid-phase intermolecular interactions alone would suggest. The large contribution from the vapour phase intermolecular interactions, determined by Δ_diss_*H*(CA), effectively cancels out a large amount of the liquid-phase intermolecular interactions contributing to Δ_vap_*H* (see equation (8.1)).

### 8.6. Why are *ced*_IP_ and *V*_m_^−1^ linearly correlated?

Both Δ_vap_*H* and *V*_m_ feature in the *ced*_IP_ equation (equation (4.1)). However, there is a very good linear correlation between *ced*_IP_ and $V_{m}^{-1}$ (figure 3*a*, and no correlation between Δ_vap_*H*_298_ and $V_{m}^{-1}$), showing that *V*_m_ is a far more important variable for *ced*_IP_ than Δ_vap_*H*. The range of Δ_vap_*H*_298_ values for ILs is relatively small, 130 to 200 kJ mol^−1^, approximately 50% increase from the smallest to the largest value. The range of *V*_m_, 150 to 600 cm^3^ mol^−1^ (table 1), approximately 400% increase from the smallest to the largest value. Hence, *V*_m_ is a far more important variable in *ced*_IP_ than Δ_vap_*H*_298_. *V*_m_ does not dominate *ced*_ML_, i.e. Δ_vap_*H*_298_ is an important variable for *ced*_ML_ (electronic supplementary material, table S5) [32]. It is important to unpack why this is the case.

The relatively small variation in Δ_vap_*H*_298_ for ILs with a large variation in structures reflects that all ILs have considerable electrostatic and vdW intermolecular interaction contributions to Δ_vap_*H*_298_ (§5.4), and that large changes in IL structure, and hence large changes in *V*_m_, do not have a significant impact on these contributions. Additionally, increasing IL size can either increase or decrease Δ_vap_*H*. When *n* is increased (i.e. $V_{m}^{-1}$ decreased) from *n* = 2 to *n* = 8, Δ_vap_*H*_298_ increases (§5.1). When the anion size is increased (i.e. $V_{m}^{-1}$ decreased) from [BF_4_] ^−^ to [NTf_2_] ^−^, Δ_vap_*H*_298_ decreases (§5.1). So by increasing *V*_m_, it is possible either to increase or decrease Δ_vap_*H*_298_. Hence, a large change in *V*_m_ can lead to a small change in Δ_vap_*H*_298_. Therefore, it is understandable why Δ_vap_*H*_298_ and $V_{m}^{-1}$ are not correlated, and furthermore, why $V_{m}^{-1}$ dominates *ced*_IP_.

## 9. Outlook: the challenges remaining

Knowledge of cohesive energy density, *ced*, for ILs has advanced a great deal in recent years. It is no longer acceptable to state that *ced* cannot be directly experimentally measured for ILs. Significant progress has been made on using *ced* values for ILs to test theories of intermolecular interactions developed for molecular liquids.

There are many challenges ahead for experimental measurements of *ced* for ILs; there are also challenges for calculations and simulations related to *ced*. Many of these challenges are focused around making vaporization measurements for ILs that do not contain the [NTf_2_] ^−^ anion; IL vaporization using Knudsen effusion apparatus has only been successfully achieved for [cation][NTf_2_] ILs to date. A summary of areas that could be explored are given in table 6.

**Table 6.** Summary of the challenges still remaining for intermolecular interactions of ILs.

experiments/calculations needed	ILs	information that could be accessed about intermolecular interactions of ILs
high sensitivity MS to determine ionic vapour composition at *T* = 298 K	any ILs	Calculations have suggested that neutral ion pairs are not the most favoured ionic vapour composition at *T* = 298 K. Such data are needed to have complete confidence in extrapolation of Δ_vap_*H* data to *T* = 298 K.
Knudsen effusion MS to determine ionic vapour composition	any ILs apart from [C*_n_*C_1_Im][NTf_2_]; ILs expected to have low Δ_vap_*H* and high thermal stability would be good candidates (perhaps [C_2_C_1_Im][FAP])	To demonstrate that the equilibrium ionic vapour composition of ILs (other than [C*_n_*C_1_Im][NTf_2_]) is neutral ion pairs. Langmuir vaporization studies for a wide range of ILs strongly suggest that the equilibrium ionic vapour composition for all ILs is neutral ion pairs; however, no experimental has been recorded.
Knudsen effusion Δ_vap_*H*	any ILs apart from [C*_n_*C_1_Im][NTf_2_] for which Δ_vap_*H* has already been measured using Langmuir vaporization; ILs expected to have low Δ_vap_*H* and high thermal stability would be good candidates (perhaps [C_2_C_1_Im][FAP])	To demonstrate that measuring Δ_vap_*H* using the non-equilibrium Langmuir gives the same Δ_vap_*H* values as the Knudsen effusion method, i.e. that there is no significant kinetic effect for Langmuir vaporization for ILs other than [C*_n_*C_1_Im][NTf_2_].
Δ_vap_*H* at very different temperatures	any ILs apart from [C*_n_*C_1_Im][NTf_2_]	To obtain Δ^g^_l_*C*_p_ directly from experiment for ILs other than [C*_n_*C_1_Im][NTf_2_]. Such data would allow validation of Δ^g^_l_*C*_p_ values from other methods.
Δ_vap_*H*	ILs with small anions and long alkyl chains, e.g. [C_8_C_1_Im][SCN]	More data for such ILs has the potential to give significant insight into the underlying interactions that determine *G*_IL_, and therefore, *γ*.
*γ* and *ρ*	any IL for which Δ_vap_*H* has already been measured, but either *γ* or *ρ* has not been measured	To provide more *ced*_IP_ and *G*_IL_ data, which will allow further insight into intermolecular interactions without the need for further challenging measurements of Δ_vap_*H*.
Δ_des_*H*	any ILs	Delta;_des_*H*(total) provides a measure of the total intermolecular interaction for an IL; hence, *ced*_C+A_ can be obtained. Currently Δ_des_*H*(total) data have only been published for [C_2_C_1_Im][NTf_2_]. Having stated in §8.4 that *ced*_IP_ may be more useful than *ced*_C+A_ for correlating with other IL properties, it might appear that knowledge of *ced*_C+A_ is not useful; that is very much not the case. A key question is: are *ced*_IP_ versus *ced*_C+A_ linearly correlated? Do they essentially measure the same thing? Does size matter for *ced*_C+A_ as much as it does for *ced*_IP_? How much does counterion affect Δ_des_*H*? Is desorption really enthalpically favourable for larger ion clusters?
calculations of Δ_diss_*H*(CA)	any ILs	How does Δ_diss_*H*(CA) vary with alkyl chain length? How does Δ_diss_*H*(CA) vary with the anion? Do electrostatic intermolecular interactions dominate Δ_diss_*H*(CA) for all ILs?
calculations of Δ_des_*H*(total) to get *ced*_C+A_	any ILs	*ced*_C+A_ is very hard to measure experimentally, so insight into *ced*_C+A_ (and, therefore, the total intermolecular interactions for ILs) is needed.
calculations of vdW and electrostatic contributions to Δ_des_*H*(total) and, therefore, to *ced*_C+A_	any ILs	Knowledge of the vdW and electrostatic contributions to IL total intermolecular interaction will give significant insight into the underlying reasons for trends. Do electrostatic intermolecular interactions dominate *ced*_C+A_ for all ILs?

## 10. Conclusion

*ced*_ML_ values have historically been very useful for understanding properties of molecular liquids, both as liquids and as solvents. For ILs, a general conclusion would be that many attempts at correlations between *ced*_IP_ and another property have proven unsuccessful to date. The lack of a correlation in itself can provide useful information about the underlying assumptions involved in those theories, and provide insight into IL properties and intermolecular interactions.

In spite of the general lack of success in finding correlations of *ced*_IP_ to other IL properties, several positive linear correlations have been found. The excellent linear correlation between two variables derived solely from experimental data, *ced*_IP_ and $V_{m}^{-1}$, is an exciting development for ILs. Clearly, size matters when judging intermolecular interaction strengths for ILs. From this correlation, the ability to make *a priori* predictions of *ced*_IP_ without the need for synthesizing or characterizing an IL is a huge step forward in understanding IL intermolecular interactions, both in terms of being able to readily predict *ced*_IP_ in itself, but also giving the ability to test theories for ILs against other physical property data.

The good linear correlation between two variables derived solely from experimental data, *ced*_IP_ and *G*, is a further exciting development for ILs. This correlation gives significant insight into IL intermolecular interactions. However, further insight could be gained if more data were available for *ced*_IP_ and *G*, particularly for ILs with long alkyl chains and small anions.

ILs have very strong intermolecular interactions relative to most molecular liquids, as evidenced by the very large *ced*_C+A_ value, presented here for the first time. Electrostatic intermolecular interactions dominate for ILs, as might be expected for liquids composed solely of ions.

ILs are not involatile solely due to their ionic nature, and therefore, due to strong electrostatic interactions. Their vapour composition—neutral ions pairs—and their relatively large size (giving considerable vdW intermolecular interactions) contribute greatly too.

## Supplementary Material

Quantifying intermolecular interactions of ionic liquids using cohesive energy densities

## Data accessibility

All data used in this publication is available in the literature. A summary of the data used is accessible in the electronic supplementary material.
