# Supplementary material for: Quantifying intermolecular interactions of ionic liquids using cohesive energy densities
Source: R Soc Open Sci. 2017 Dec 6;4(12):171223. doi: 10.1098/rsos.171223 (PMC5750021; doi:10.1098/rsos.171223)
Supplement: Quantifying intermolecular interactions of ionic liquids using cohesive energy densities [file rsos171223supp1.pdf]

Quantifying intermolecular interactions of ionic liquids using cohesive energy densities

Kevin R. J. Lovelock
Department of Chemistry, University of Reading, UK
Contact E-mail: k.r.j.lovelock@reading.ac.uk

Electronic Supplementary Information

Section S1. IL vaporisation mechanism Page 2

Section S2. IL abbreviations used in this Review Pages 3 to 4

Section S3. Summaries of the $\Delta_{vap}H$ data used in this Review Pages 5 to 22

Section S4. Further figures showing correlations Page 23

Section S5. References Pages 24 to 26

Section S1. IL vaporisation mechanism

The kinetics of vaporisation will be affected by the IL vaporisation mechanism. However, long–lived ion pairs do not exist in the liquid phase for ILs.[1] Most computational studies of IL vaporisation have focused on the equilibrium composition, with only a few studies focusing on the vaporisation mechanism.[2-4] Kirchner and co–workers have proposed a mechanism for vaporisation where cations and anions diffuse separately as individual ions to the IL–gas surface. The ion pair is then formed at the IL–gas surface, and the ion pair vaporises, *i.e*. an ion diffuses to the IL–gas surface and then picks up a counterion (that has also diffused to the IL–gas surface) to form an ion pair and vaporise. Hessey and Jones measured the entropy change on forming the vaporisation transition state as +50 J K^{-1} mol^{-1} for $[C_2C_1Im][NTf_2]$.[5] They concluded that this large positive value is indicative of a large increase in mobility on forming the vaporisation transition state, indicative of vaporisation of an ion pair from within the IL–gas surface, rather than on top of the IL–gas surface.[5] ILs that are labelled as pure in the bulk (using techniques such as NMR spectroscopy) can show IL–gas surface contamination;[6] such contamination may have an effect on the vaporisation mechanism and therefore, the non–equilibrium energetics. For IL vaporisation under non–equilibrium conditions (followed by vapour phase IL ion pair cooling) a range of vapour phase neutral ion pair conformers have been shown to form.[7] Under equilibrium conditions it might be expected that the lowest energy conformer forms; however, for non–equilibrium conditions the presence of a range of conformers may affect the energetics of vaporisation.

Section S2. IL abbreviations used in this Review

Cation abbreviation	Cation name
$[C_1C_1Im]^+$	1,3–dimethylimidazolium
$[C_2C_1Im]^+$	1–ethyl–3–methylimidazolium
$[C_3C_1Im]^+$	1–propyl–3–methylimidazolium
$[C_4C_1Im]^+$	1–butyl–3–methylimidazolium
$[C_5C_1Im]^+$	1–pentyl–3–methylimidazolium
$[C_6C_1Im]^+$	1–hexyl–3–methylimidazolium
$[C_7C_1Im]^+$	1–heptyl–3–methylimidazolium
$[C_8C_1Im]^+$	1–octyl–3–methylimidazolium
$[C_{10}C_1Im]^+$	1–decyl–3–methylimidazolium
$[C_{12}C_1Im]^+$	1–dodecyl–3–methylimidazolium
$[C_{14}C_1Im]^+$	1–tetradecyl–3–methylimidazolium
$[C_{16}C_1Im]^+$	1–hexadecyl–3–methylimidazolium
$[C_{18}C_1Im]^+$	1–octadecyl–3–methylimidazolium
$[C_3C_2Im]^+$	1–ethyl–3–propylimidazolium
$[C_1C_1C_1Im]$	1,2,3–trimethylimidazolium
$[C_3C_1C_1Im]^+$	1,2–dimethyl–3–propylimidazolium
$[C_4C_1C_1Im]^+$	1,2–dimethyl–3–butylimidazolium
$[C_3(C_1Im)_2]^{2+}$	1,3–bis(3–methylimidazolium–1–yl)propane
$[C_1C_1C_1BIm]^+$	1,2,3–trimethyl–benzimidazolium
$[Me(EG)_1C_1Im]^+$	3–(2–methoxy–ethyl)–1–methylimidazolium
$[Me(EG)_2C_1Im]^+$	3–[2–(2–methoxy–ethoxy)–ethyl]–1–methylimidazolium
$[Me(EG)_3C_1Im]^+$	3–[2–[2–(2–methoxy–ethoxy)–ethoxy]–ethyl]–1–methylimidazolium
$[Me(EG)_1C_1C_1Im]^+$	3–(2–methoxy–ethyl)–1,2–dimethylimidazolium
$[Me(EG)_2C_1C_1Im]^+$	3–[2–(2–methoxy–ethoxy)–ethyl]–1,2–dimethylimidazolium
$[Me(EG)_3C_1C_1Im]^+$	3–[2–[2–(2–methoxy–ethoxy)–ethoxy]–ethyl]–1,2–dimethylimidazolium
$[(C_2F_5C_2H_4)C_1Im]^+$	1–methyl–3–(3,3,4,4,4–pentafluorobutyl)–imidazolium
$[(C_4F_9C_2H_4)C_1Im]^+$	1–methyl–3–(3,3,4,4,5,5,6,6,6–nonafluorohexyl)–imidazolium
$[(C_6F_{13}C_2H_4)C_1Im]^+$	1–methyl–3–(3,3,4,4,5,5,6,6,7,7,8,8,8–tridecafluorooctyl)–imidazolium
$[(C_8F_{17}C_2H_4)C_1Im]^+$	1–methyl–3–(3,3,4,4,5,5,6,6,7,7,8,8,9,9,10,10,10–heptadecafluorodecyl)–imidazolium
$[i–C_3C_1Im]^+$	1–methyl–3–(1–methylethyl)–imidazolium
$[i–C_4C_1Im]^+$	1–methyl–3–(2–methylpropyl)–imidazolium
$[s–C_4C_1Im]^+$	1–methyl–3–(1–methylpropyl)–imidazolium
$[cyclo–C_3–CH_2–C_1Im]^+$	1–methyl–3–cyclopropylmethyl–imidazolium
$[cyclo–C_5C_1Im]^+$	1–methyl–3–cyclopentyl–imidazolium
$[cyclo–C_6–CH_2–C_1Im]^+$	1–methyl–3–cyclohexylmethyl–imidazolium
$[Phenyl–Im]^+$ 1	3–butyl–1–phenyl–imidazolium
$[Phenyl–Im]^+$ 2	3-butyl–1–(2–methylphenyl)–imidazolium
$[Phenyl–Im]^+$ 3	3-butyl–1–(4–methylphenyl)–imidazolium
$[Phenyl–Im]^+$ 4	3-butyl–1–(2–methoxyphenyl)–imidazolium
$[Phenyl–Im]^+$ 5	3-butyl–1–(4–methoxyphenyl)–imidazolium
$[C_2C_2Im]^+$	1,3–diethylimidazolium
$[C_3C_3Im]^+$	1,3–dipropylimidazolium
$[C_4C_4Im]^+$	1,3–dibutylimidazolium
$[C_5C_5Im]^+$	1,3–dipentylimidazolium
$[C_6C_6Im]^+$	1,3–dihexylimidazolium
$[C_7C_7Im]^+$	1,3–diheptylimidazolium
$[C_8C_8Im]^+$	1,3–dioctylimidazolium
$[C_9C_9Im]^+$	1,3–dinonylimidazolium
$[C_{10}C_{10}Im]^+$	1,3–didecylimidazolium
$[C_3C_1Pyrr]^+$	1–propyl–1–methylpyrrolidinium
$[C_4C_1Pyrr]^+$	1–butyl–1–methylpyrrolidinium
$[C_5C_1Pyrr]^+$	1–pentyl–1–methylpyrrolidinium
$[C_6C_1Pyrr]^+$	1–hexyl–1–methylpyrrolidinium
$[C_7C_1Pyrr]^+$	1–heptyl–1–methylpyrrolidinium
$[C_8C_1Pyrr]^+$	1–octyl–1–methylpyrrolidinium
$[C_{10}C_1Pyrr]^+$	1–decyl–1–methylpyrrolidinium
$[C_2Py]^+$	1–ethylpyridinium
$[C_3Py]^+$	1–propylpyridinium
$[C_4Py]^+$	1–butylpyridinium
$[C_5Py]^+$	1–pentylpyridinium
$[C_6Py]^+$	1–hexylpyridinium
$[{}^2C_2{}^1C_2Py]^+$	1,2–diethylpyridinium
$[{}^2C_3{}^1C_2Py]^+$	1–ethyl–2–propylpyridinium

$[^2C_4{}^1C_2Py]^+$	1–ethyl–2–butylpyridinium
$[^2C_5{}^1C_2Py]^+$	1–ethyl–2–pentylpyridinium
$[^2C_6{}^1C_2Py]^+$	1–ethyl–2–hexylpyridinium
$[^2C_7{}^1C_2Py]^+$	1–ethyl–2–heptylpyridinium
$[^2C_8{}^1C_2Py]^+$	1–ethyl–2–octylpyridinium
$[^2C_9{}^1C_2Py]^+$	1–ethyl–2–nonylpyridinium
$[^2C_{10}{}^1C_2Py]^+$	1–ethyl–2–decylpyridinium
$[^3C_1{}^1C_4Py]^+$	1–butyl–3–methylpyridinium
$[^4C_1{}^1C_4Py]^+$	1–butyl–4–methylpyridinium
$[N_{4,4,4,4}]^+$	tetrabutylammonium
$[P_{6,6,6,14}]^+$	trihexyl(tetradecyl)phosphonium
$[C_2(C_1)_4iU]^+$	O–ethyl–N,N,N',N'–tetramethylisouronium

Table S1. Cation abbreviations and names for ionic liquids included in this Review.

Anion abbreviation	Anion name
$[NTf_2]^-$	bis[(trifluoromethane)sulfonyl]imide
$[NPf_2]^-$	bis[(pentafluoroethane)sulfonyl]imide
$[BF_4]^-$	tetrafluoroborate
$[PF_6]^-$	hexaflurophosphate
$[TfO]^-$	trifluoromethanesulfonate
$[N(CN)_2]^-$	dicyanamide
Cl^-	chloride
I^-	iodide
$[C_1SO_4]^-$	methylsulfate
$[C_2SO_4]^-$	ethylsulfate
$[C_4SO_4]^-$	butylsulfate
$[C_8SO_4]^-$	octylsulfate
$[(C_2F_5)_2PO_2]^-$	bis(pentafluoroethyl)phosphinate
$[(C_2H_5O)_2PO_2]^-$	diethylphosphate
$[CF_3CO_2]^-$	trifluroacetate
$[TOS]^-$	tosylate
$[SCN]^-$	thiocyanate
$[N(CN)_2]^-$	dicyanamide
$[C(CN)_3]^-$	tricyanomethanide
$[B(CN)_4]^-$	tetracyanoborate
$[FeCl_4]^-$	tetrachloroferrate
$[FAP]^-$	tris(pentafluoroethyl)trifluorophosphate
$[PO_2(C_2F_5)_2]^-$	bis(pentafluoroethyl)phosphinate

Table S2. Anion abbreviations and names for ionic liquids included in this Review.

Section S3. Summaries of the $\Delta_{vap}H$ data used in this Review

	Ionic liquid	Method	$\Delta_{vap}H_T$ / kJ mol^{-1}	*T* / K	Year	Ref.	DOI
1	[C_1C_1Im][NTf_2]	QCM	120	381.6	2013	[8]	http://pubs.acs.org/doi/abs/10.1021/jp311429r
1	[C_1C_1Im][NTf_2]	KEQCM	117	484.65	2014	[9]	http://dx.doi.org/10.1016/j.jct.2013.09.020
1	[C_1C_1Im][NTf_2]	TGA	104	590	2013	[8]	http://pubs.acs.org/doi/abs/10.1021/jp311429r
2	[C_2C_1Im][NTf_2]	QCM	119	378	2013	[8]	http://pubs.acs.org/doi/abs/10.1021/jp311429r
2	[C_2C_1Im][NTf_2]	TPD	121	398	2010	[10]	http://pubs.rsc.org/en/Content/ArticleLanding/2010/CP/c004197a
2	[C_2C_1Im][NTf_2]	TPD	122	430	2007	[11]	http://pubs.rsc.org/en/Content/ArticleLanding/2007/CP/b615137j
2	[C_2C_1Im][NTf_2]	KEMS	121	459	2015	[12]	http://onlinelibrary.wiley.com/doi/10.1002/rcm.7214/abstract
2	[C_2C_1Im][NTf_2]	Knudsen mass loss	119	463	2006	[13]	http://pubs.acs.org/doi/abs/10.1021/jp060896f
2	[C_2C_1Im][NTf_2]	KEQCM	115	464.36	2011	[14]	http://pubs.acs.org/doi/abs/10.1021/jp2049316
2	[C_2C_1Im][NTf_2]	TGA	121	495.5	2008	[15]	http://pubs.acs.org/doi/abs/10.1021/jp805340f
2	[C_2C_1Im][NTf_2]	transpiration	115	516.2	2007	[16]	http://pubs.acs.org/doi/abs/10.1021/ja0679174
2	[C_2C_1Im][NTf_2]	TGA	111	520.2	2013	[8]	http://pubs.acs.org/doi/abs/10.1021/jp311429r
2	[C_2C_1Im][NTf_2]	UV ab	112	573	2010	[17]	http://pubs.rsc.org/en/Content/ArticleLanding/2010/CP/c001101k
2	[C_2C_1Im][NTf_2]	Calorimetry (He)	108	625	2014	[18]	http://pubs.rsc.org/en/Content/ArticleLanding/2014/CP/c3cp54325k#!divAbstract
2	[C_2C_1Im][NTf_2]	Calorimetry (N2)	110	625	2014	[18]	http://pubs.rsc.org/en/Content/ArticleLanding/2014/CP/c3cp54325k#!divAbstract
3	[C_3C_1Im][NTf_2]	QCM	121	380.3	2013	[8]	http://pubs.acs.org/doi/abs/10.1021/jp311429r
3	[C_3C_1Im][NTf_2]	KEQCM	113	473.04	2011	[14]	http://pubs.acs.org/doi/abs/10.1021/jp2049316
3	[C_3C_1Im][NTf_2]	TGA	109	546.2	2013	[8]	http://pubs.acs.org/doi/abs/10.1021/jp311429r
4	[C_4C_1Im][NTf_2]	QCM	124	378	2013	[8]	http://pubs.acs.org/doi/abs/10.1021/jp311429r
4	[C_4C_1Im][NTf_2]	KEML	118	427	2014	[19]	http://pubs.rsc.org/en/Content/ArticleLanding/2014/CP/c4cp01673d#!divAbstract
4	[C_4C_1Im][NTf_2]	TPD	121	440	2007	[11]	http://pubs.rsc.org/en/Content/ArticleLanding/2007/CP/b615137j
4	[C_4C_1Im][NTf_2]	KEQCM	118	475.01	2011	[14]	http://pubs.acs.org/doi/abs/10.1021/jp2049316
4	[C_4C_1Im][NTf_2]	Knudsen mass loss	118	477.6	2006	[13]	http://pubs.acs.org/doi/abs/10.1021/jp060896f
4	[C_4C_1Im][NTf_2]	TGA	119	495.5	2008	[15]	http://pubs.acs.org/doi/abs/10.1021/jp805340f
4	[C_4C_1Im][NTf_2]	TGA	114	541.8	2013	[8]	http://pubs.acs.org/doi/abs/10.1021/jp311429r
4	[C_4C_1Im][NTf_2]	UV ab	114	553	2010	[17]	http://pubs.rsc.org/en/Content/ArticleLanding/2010/CP/c001101k
4	[C_4C_1Im][NTf_2]	TGA	108	600	2009	[20]	http://pubs.rsc.org/en/Content/ArticleLanding/2009/CP/b909624h
5	[C_5C_1Im][NTf_2]	QCM	128	381.7	2013	[8]	http://pubs.acs.org/doi/abs/10.1021/jp311429r

5	$[C_5C_1Im][NTf_2]$	KEQCM	123	474.99	2011	[14]	http://pubs.acs.org/doi/abs/10.1021/jp2049316
5	$[C_5C_1Im][NTf_2]$	TGA	111	560.8	2013	[8]	http://pubs.acs.org/doi/abs/10.1021/jp311429r
6	$[C_6C_1Im][NTf_2]$	QCM	132	383	2013	[8]	http://pubs.acs.org/doi/abs/10.1021/jp311429r
6	$[C_6C_1Im][NTf_2]$	TPD	125	445	2007	[11]	http://pubs.rsc.org/en/Content/ArticleLanding/2007/CP/b615137j
6	$[C_6C_1Im][NTf_2]$	Knudsen mass loss	123	461.8	2006	[13]	http://pubs.acs.org/doi/abs/10.1021/jp060896f
6	$[C_6C_1Im][NTf_2]$	KEQCM	126	477.35	2011	[14]	http://pubs.acs.org/doi/abs/10.1021/jp2049316
6	$[C_6C_1Im][NTf_2]$	TGA	124	503	2008	[15]	http://pubs.acs.org/doi/abs/10.1021/jp805340f
6	$[C_6C_1Im][NTf_2]$	TGA	118	541.8	2013	[8]	http://pubs.acs.org/doi/abs/10.1021/jp311429r
7	$[C_7C_1Im][NTf_2]$	QCM	134	385.3	2013	[8]	http://pubs.acs.org/doi/abs/10.1021/jp311429r
7	$[C_7C_1Im][NTf_2]$	KEQCM	129	478.92	2011	[14]	http://pubs.acs.org/doi/abs/10.1021/jp2049316
7	$[C_7C_1Im][NTf_2]$	TGA	114	553.7	2013	[8]	http://pubs.acs.org/doi/abs/10.1021/jp311429r
8	$[C_8C_1Im][NTf_2]$	QCM	137	387	2013	[8]	http://pubs.acs.org/doi/abs/10.1021/jp311429r
8	$[C_8C_1Im][NTf_2]$	TPD	135	450	2007	[11]	http://pubs.rsc.org/en/Content/ArticleLanding/2007/CP/b615137j
8	$[C_8C_1Im][NTf_2]$	Knudsen mass loss	132	475.2	2006	[13]	http://pubs.acs.org/doi/abs/10.1021/jp060896f
8	$[C_8C_1Im][NTf_2]$	KEQCM	129	484.1	2011	[14]	http://pubs.acs.org/doi/abs/10.1021/jp2049316
8	$[C_8C_1Im][NTf_2]$	TGA	132	503	2008	[15]	http://pubs.acs.org/doi/abs/10.1021/jp805340f
8	$[C_8C_1Im][NTf_2]$	TGA	123	541.8	2013	[8]	http://pubs.acs.org/doi/abs/10.1021/jp311429r
9	$[C_{10}C_1Im][NTf_2]$	QCM	143	394.6	2013	[8]	http://pubs.acs.org/doi/abs/10.1021/jp311429r
9	$[C_{10}C_1Im][NTf_2]$	KEQCM	136	487.38	2011	[14]	http://pubs.acs.org/doi/abs/10.1021/jp2049316
9	$[C_{10}C_1Im][NTf_2]$	TGA	134	510	2008	[15]	http://pubs.acs.org/doi/abs/10.1021/jp805340f
9	$[C_{10}C_1Im][NTf_2]$	TGA	125	522	2013	[8]	http://pubs.acs.org/doi/abs/10.1021/jp311429r
10	$[C_{12}C_1Im][NTf_2]$	QCM	147	408.4	2013	[8]	http://pubs.acs.org/doi/abs/10.1021/jp311429r
10	$[C_{12}C_1Im][NTf_2]$	KEQCM	140	486.85	2011	[14]	http://pubs.acs.org/doi/abs/10.1021/jp2049316
10	$[C_{12}C_1Im][NTf_2]$	TGA	126	533.5	2013	[8]	http://pubs.acs.org/doi/abs/10.1021/jp311429r
11	$[C_{14}C_1Im][NTf_2]$	QCM	153	416.2	2013	[8]	http://pubs.acs.org/doi/abs/10.1021/jp311429r
11	$[C_{14}C_1Im][NTf_2]$	KEQCM	141	513.06	2016	[21]	http://www.sciencedirect.com/science/article/pii/S0021961416000598
11	$[C_{14}C_1Im][NTf_2]$	TGA	133	541.8	2013	[8]	http://pubs.acs.org/doi/abs/10.1021/jp311429r
12	$[C_{16}C_1Im][NTf_2]$	QCM	156	424.8	2013	[8]	http://pubs.acs.org/doi/abs/10.1021/jp311429r
12	$[C_{16}C_1Im][NTf_2]$	KEQCM	147	520.57	2016	[21]	http://www.sciencedirect.com/science/article/pii/S0021961416000598

12	[$C_{16}C_1$Im][NTf_2]	TGA	136	541.8	2013	[8]	http://pubs.acs.org/doi/abs/10.1021/jp311429r
13	[$C_{18}C_1$Im][NTf_2]	QCM	162	430.3	2013	[8]	http://pubs.acs.org/doi/abs/10.1021/jp311429r
13	[$C_{18}C_1$Im][NTf_2]	TGA	139	582.5	2013	[8]	http://pubs.acs.org/doi/abs/10.1021/jp311429r
14	[$C_1C_1C_1$Im][NTf_2]	QCM	122	396	2011	[22]	http://pubs.rsc.org/en/Content/ArticleLanding/2011/CP/c1cp20732f#!divAbstract
15	[$C_3C_1C_1$Im][NTf_2]	KEMS	124	464	2015	[12]	http://onlinelibrary.wiley.com/doi/10.1002/rcm.7214/abstract
15	[$C_3C_1C_1$Im][NTf_2]	KEQCM	117	485.57	2017	[23]	http://pubs.rsc.org/en/Content/ArticleLanding/2017/CP/C6CP08451F#!divAbstract
15	[$C_3C_1C_1$Im][NTf_2]	TGA	130	503	2008	[15]	http://pubs.acs.org/doi/abs/10.1021/jp805340f
15	[$C_4C_1C_1$Im][NTf_2]	KEQCM	121	483.09	2017	[23]	http://pubs.rsc.org/en/Content/ArticleLanding/2017/CP/C6CP08451F#!divAbstract
16	[$C_4C_1C_1$Im][NTf_2]	QCM	130	503	2011	[22]	http://pubs.rsc.org/en/Content/ArticleLanding/2011/CP/c1cp20732f#!divAbstract
17	[$C_3(C_1Im)_2$][NTf_2]$_2$	TPD	158	545	2009	[24]	http://onlinelibrary.wiley.com/doi/10.1002/cphc.200800690/abstract
18	[$C_1C_1C_1$BIm][NTf_2]	QCM	134	397.5	2013	[25]	http://dx.doi.org/10.1524/zpch.2013.0312
18	[$C_1C_1C_1$BIm][NTf_2]	TGA	122	581.8	2013	[25]	http://dx.doi.org/10.1524/zpch.2013.0312
19	[$Me(EG)_1C_1$Im][NTf_2]	QCM	118	365.5	2013	[26]	dx.doi.org/10.1021/ie402664c
19	[$Me(EG)_1C_1$Im][NTf_2]	TGA	110	542.4	2013	[26]	dx.doi.org/10.1021/ie402664c
20	[$Me(EG)_2C_1$Im][NTf_2]	QCM	127	378.3	2013	[26]	dx.doi.org/10.1021/ie402664c
20	[$Me(EG)_2C_1$Im][NTf_2]	TGA	114	543.9	2013	[26]	dx.doi.org/10.1021/ie402664c
21	[$Me(EG)_3C_1$Im][NTf_2]	QCM	134	388.2	2013	[26]	dx.doi.org/10.1021/ie402664c
21	[$Me(EG)_3C_1$Im][NTf_2]	TGA	118	551	2013	[26]	dx.doi.org/10.1021/ie402664c
22	[$Me(EG)_1C_1C_1$Im][NTf_2]	QCM	123	381.1	2013	[26]	dx.doi.org/10.1021/ie402664c
22	[$Me(EG)_1C_1C_1$Im][NTf_2]	TGA	103	543.5	2013	[26]	dx.doi.org/10.1021/ie402664c
23	[$Me(EG)_2C_1C_1$Im][NTf_2]	QCM	129	390.9	2013	[26]	dx.doi.org/10.1021/ie402664c
23	[$Me(EG)_2C_1C_1$Im][NTf_2]	TGA	110	543.9	2013	[26]	dx.doi.org/10.1021/ie402664c
24	[$Me(EG)_3C_1C_1$Im][NTf_2]	QCM	134	400.9	2013	[26]	dx.doi.org/10.1021/ie402664c
25	[$(C_2F_5C_2H_4)C_1$Im][NTf_2]	QCM−Langmuir	130	370.1	2015	[27]	http://www.sciencedirect.com/science/article/pii/S0040603115001653
25	[$(C_2F_5C_2H_4)C_1$Im][NTf_2]	TGA	114	527.7	2015	[27]	http://www.sciencedirect.com/science/article/pii/S0040603115001653

26	$[(C_4F_9C_2H_4)C_1Im][NTf_2]$	QCM−Langmuir	138	371.1	2015	[27]	http://www.sciencedirect.com/science/article/pii/S0040603115001653
26	$[(C_4F_9C_2H_4)C_1Im][NTf_2]$	TGA	116	532.48	2015	[27]	http://www.sciencedirect.com/science/article/pii/S0040603115001653
27	$[(C_6F_{13}C_2H_4)C_1Im][NTf_2]$	QCM−Langmuir	143	376.2	2015	[27]	http://www.sciencedirect.com/science/article/pii/S0040603115001653
27	$[(C_6F_{13}C_2H_4)C_1Im][NTf_2]$	TGA	126	532.3	2015	[27]	http://www.sciencedirect.com/science/article/pii/S0040603115001653
28	$[(C_8F_{17}C_2H_4)C_1Im][NTf_2]$	QCM−Langmuir	148	381.4	2015	[27]	http://www.sciencedirect.com/science/article/pii/S0040603115001653
28	$[(C_8F_{17}C_2H_4)C_1Im][NTf_2]$	TGA	128	531.8	2015	[27]	http://www.sciencedirect.com/science/article/pii/S0040603115001653
29	$[i–C_3C_1Im][NTf_2]$	QCM−Langmuir	123	366.3	2016	[28]	http://www.sciencedirect.com/science/article/pii/S0021961415003638
30	$[i–C_4C_1Im][NTf_2]$	QCM−Langmuir	126	368.7	2016	[28]	http://www.sciencedirect.com/science/article/pii/S0021961415003638
31	$[s–C_4C_1Im][NTf_2]$	QCM−Langmuir	123	368.6	2016	[28]	http://www.sciencedirect.com/science/article/pii/S0021961415003638
32	$[cyclo–C_3–CH_2–C_1Im][NTf_2]$	QCM−Langmuir	127	373.5	2016	[28]	http://www.sciencedirect.com/science/article/pii/S0021961415003638
33	$[cyclo–C_5C_1Im][NTf_2]$	QCM−Langmuir	127	373.7	2016	[28]	http://www.sciencedirect.com/science/article/pii/S0021961415003638
34	$[cyclo–C_6–CH_2–C_1Im][NTf_2]$	QCM−Langmuir	134	383.6	2016	[28]	http://www.sciencedirect.com/science/article/pii/S0021961415003638
35	$[Phenyl–Im][NTf_2]$ 1	QCM−Langmuir	132	384.8	2017	[29]	dx.doi.org/10.1002/zaac.201600333
36	$[Phenyl–Im][NTf_2]$ 2	QCM−Langmuir	134	373.3	2017	[29]	dx.doi.org/10.1002/zaac.201600333
37	$[Phenyl–Im][NTf_2]$ 3	QCM−Langmuir	142	381	2017	[29]	dx.doi.org/10.1002/zaac.201600333
38	$[Phenyl–Im][NTf_2]$ 4	QCM−Langmuir	137	387.3	2017	[29]	dx.doi.org/10.1002/zaac.201600333
39	$[Phenyl–Im][NTf_2]$ 5	QCM−Langmuir	142	396.1	2017	[29]	dx.doi.org/10.1002/zaac.201600333
40	$[C_2C_2Im][NTf_2]$	KEQCM	109	467.61	2012	[30]	http://pubs.acs.org/doi/abs/10.1021/jp306937f
41	$[C_3C_3Im][NTf_2]$	KEQCM	114	471.6	2012	[30]	http://pubs.acs.org/doi/abs/10.1021/jp306937f
42	$[C_4C_4Im][NTf_2]$	KEQCM	117	471.58	2012	[30]	http://pubs.acs.org/doi/abs/10.1021/jp306937f
43	$[C_5C_5Im][NTf_2]$	KEQCM	124	479.54	2012	[30]	http://pubs.acs.org/doi/abs/10.1021/jp306937f
44	$[C_6C_6Im][NTf_2]$	KEQCM	138	479.6	2012	[30]	http://pubs.acs.org/doi/abs/10.1021/jp306937f
45	$[C_3C_2Im][NTf_2]$	KEQCM	115	476.6	2014	[9]	http://dx.doi.org/10.1016/j.jct.2013.09.020
46	$[C_7C_7Im][NTf_2]$	KEQCM	131	493.39	2014	[31]	http://aip.scitation.org/doi/10.1063/1.4896704
47	$[C_8C_8Im][NTf_2]$	KEQCM	126	494.33	2014	[31]	http://aip.scitation.org/doi/10.1063/1.4896704
48	$[C_9C_9Im][NTf_2]$	KEQCM	129	508.1	2014	[31]	http://aip.scitation.org/doi/10.1063/1.4896704
49	$[C_{10}C_{10}Im][NTf_2]$	KEQCM	125	502.37	2014	[31]	http://aip.scitation.org/doi/10.1063/1.4896704

50	[C_3C_1Pyrr][NTf_2]	QCM	131	415.7	2014	[32]	http://dx.doi.org/10.1016/j.molliq.2013.07.018
50	[C_3C_1Pyrr][NTf_2]	TGA	126	554.2	2014	[32]	http://dx.doi.org/10.1016/j.molliq.2013.07.018
51	[C_4C_1Pyrr][NTf_2]	QCM	134	415.8	2014	[32]	http://dx.doi.org/10.1016/j.molliq.2013.07.018
51	[C_4C_1Pyrr][NTf_2]	TPD	136	470	2009	[33]	http://pubs.rsc.org/en/Content/ArticleLanding/2009/CP/b908209c
51	[C_4C_1Pyrr][NTf_2]	TGA	124	554.3	2014	[32]	http://dx.doi.org/10.1016/j.molliq.2013.07.018
52	[C_5C_1Pyrr][NTf_2]	QCM	135	405.7	2014	[32]	http://dx.doi.org/10.1016/j.molliq.2013.07.018
53	[C_6C_1Pyrr][NTf_2]	QCM	138	405.9	2014	[32]	http://dx.doi.org/10.1016/j.molliq.2013.07.018
53	[C_6C_1Pyrr][NTf_2]	TPD	141	460	2009	[33]	http://pubs.rsc.org/en/Content/ArticleLanding/2009/CP/b908209c
53	[C_6C_1Pyrr][NTf_2]	TGA	124	554.7	2014	[32]	http://dx.doi.org/10.1016/j.molliq.2013.07.018
54	[C_7C_1Pyrr][NTf_2]	QCM	142	408.3	2014	[32]	http://dx.doi.org/10.1016/j.molliq.2013.07.018
54	[C_7C_1Pyrr][NTf_2]	TGA	131	554.9	2014	[32]	http://dx.doi.org/10.1016/j.molliq.2013.07.018
55	[C_8C_1Pyrr][NTf_2]	QCM	144	410.9	2014	[32]	http://dx.doi.org/10.1016/j.molliq.2013.07.018
55	[C_8C_1Pyrr][NTf_2]	TPD	145	470	2009	[33]	http://pubs.rsc.org/en/Content/ArticleLanding/2009/CP/b908209c
55	[C_8C_1Pyrr][NTf_2]	TGA	130	562.1	2014	[32]	http://dx.doi.org/10.1016/j.molliq.2013.07.018
56	[$C_{10}C_1$Pyrr][NTf_2]	QCM	149	418.4	2014	[32]	http://dx.doi.org/10.1016/j.molliq.2013.07.018
56	[$C_{10}C_1$Pyrr][NTf_2]	TGA	135	562.3	2014	[32]	http://dx.doi.org/10.1016/j.molliq.2013.07.018
57	[C_2Py][NTf_2]	QCM	125	400.6	2012	[34]	http://link.springer.com/article/10.1007%2Fs11426-012-4662-2
57	[C_2Py][NTf_2]	KEQCM	120	498.58	2013	[35]	http://www.sciencedirect.com/science/article/pii/S0009261413011081
58	[C_3Py][NTf_2]	QCM	128	398.2	2012	[34]	http://link.springer.com/article/10.1007%2Fs11426-012-4662-2
58	[C_3Py][NTf_2]	KEQCM	124	504.5	2013	[35]	http://www.sciencedirect.com/science/article/pii/S0009261413011081
59	[C_4Py][NTf_2]	QCM	131	399.5	2012	[34]	http://link.springer.com/article/10.1007%2Fs11426-012-4662-2
59	[C_4Py][NTf_2]	KEQCM	122	506.82	2013	[35]	http://www.sciencedirect.com/science/article/pii/S0009261413011081
59	[C_4Py][NTf_2]	UV ab	120	553	2010	[17]	http://pubs.rsc.org/en/Content/ArticleLanding/2010/CP/c001101k
60	[C_5Py][NTf2]	QCM	134	400.6	2012	[34]	http://link.springer.com/article/10.1007%2Fs11426-012-4662-2
61	[C_6Py][NTf_2]	QCM	137	405.7	2012	[34]	http://link.springer.com/article/10.1007%2Fs11426-012-4662-2

61	$[C_6Py][NTf_2]$	TPD	139	440	2009	[33]	http://pubs.rsc.org/en/Content/ArticleLanding/2009/CP/b908209c
62	$[^2C_2{}^1C_2Py][NTf_2]$	KEQCM	125	508.11	2015	[36]	http://pubs.rsc.org/en/Content/ArticleLanding/2015/CP/C4CP05191B#!divAbstract
63	$[^2C_3{}^1C_2Py][NTf_2]$	KEQCM	121	510.59	2015	[36]	http://pubs.rsc.org/en/Content/ArticleLanding/2015/CP/C4CP05191B#!divAbstract
64	$[^2C_4{}^1C_2Py][NTf_2]$	KEQCM	122	503.12	2015	[36]	http://pubs.rsc.org/en/Content/ArticleLanding/2015/CP/C4CP05191B#!divAbstract
65	$[^2C_5{}^1C_2Py][NTf_2]$	KEQCM	127	510.63	2015	[36]	http://pubs.rsc.org/en/Content/ArticleLanding/2015/CP/C4CP05191B#!divAbstract
66	$[^2C_6{}^1C_2Py][NTf_2]$	KEQCM	128	505.65	2015	[36]	http://pubs.rsc.org/en/Content/ArticleLanding/2015/CP/C4CP05191B#!divAbstract
67	$[^2C_7{}^1C_2Py][NTf_2]$	KEQCM	131	508.27	2015	[36]	http://pubs.rsc.org/en/Content/ArticleLanding/2015/CP/C4CP05191B#!divAbstract
68	$[^2C_8{}^1C_2Py][NTf_2]$	KEQCM	138	505.73	2015	[36]	http://pubs.rsc.org/en/Content/ArticleLanding/2015/CP/C4CP05191B#!divAbstract
69	$[^2C_9{}^1C_2Py][NTf_2]$	KEQCM	140	523.03	2015	[36]	http://pubs.rsc.org/en/Content/ArticleLanding/2015/CP/C4CP05191B#!divAbstract
70	$[^2C_{10}{}^1C_2Py][NTf_2]$	KEQCM	144	520.5	2015	[36]	http://pubs.rsc.org/en/Content/ArticleLanding/2015/CP/C4CP05191B#!divAbstract
71	$[C_2C_1Im][NPf_2]$	TGA	115	503	2008	[15]	http://pubs.acs.org/doi/abs/10.1021/jp805340f
71	$[C_2C_1Im][NPf_2]$	UV ab	110	553	2010	[17]	http://pubs.rsc.org/en/Content/ArticleLanding/2010/CP/c001101k
72	$[C_4C_1Im][NPf_2]$	TGA	114	503	2008	[15]	http://pubs.acs.org/doi/abs/10.1021/jp805340f
72	$[C_4C_1Im][NPf_2]$	UV ab	114	553	2010	[17]	http://pubs.rsc.org/en/Content/ArticleLanding/2010/CP/c001101k
73	$[C_6C_1Im][NPf_2]$	TGA	118	503	2008	[15]	http://pubs.acs.org/doi/abs/10.1021/jp805340f
74	$[C_6C_1Im][NPf_2]$	TGA	125	499.3	2008	[15]	http://pubs.acs.org/doi/abs/10.1021/jp805340f
75	$[C_{10}C_1Im][NPf_2]$	TGA	127	510	2008	[15]	http://pubs.acs.org/doi/abs/10.1021/jp805340f
76	$[C_3C_1C_1Im][NPf_2]$	TGA	122	503	2008	[15]	http://pubs.acs.org/doi/abs/10.1021/jp805340f
77	$[C_2C_1Im][BF_4]$	QCM	122	431.6	2012	[37]	http://onlinelibrary.wiley.com/doi/10.1002/cphc.201100879/abstract
77	$[C_2C_1Im][BF_4]$	TPD	128	515	2009	[33]	http://pubs.rsc.org/en/Content/ArticleLanding/2009/CP/b908209c
78	$[C_4C_1Im][BF_4]$	TPD	134	500	2012	[38]	http://pubs.rsc.org/en/content/articlelanding/2012/cp/c2cp23705a
79	$[C_8C_1Im][BF_4]$	TPD	141	520	2007	[11]	http://pubs.rsc.org/en/Content/ArticleLanding/2007/CP/b615137j
80	$[P_{6,6,6,14}][BF_4]$	TPD	180	490	2012	[38]	http://pubs.rsc.org/en/content/articlelanding/2012/cp/c2cp23705a
81	$[C_2Py][BF_4]$	QCM−Langmuir	136	459.4	2017	[39]	http://onlinelibrary.wiley.com/doi/10.1002/zaac.201600335/abstract
82	$[C_4Py][BF_4]$	QCM−Langmuir	140	433.8	2017	[39]	http://onlinelibrary.wiley.com/doi/10.1002/zaac.201600335/abstract

82	$[C_4Py][BF_4]$	TPD	146	510	2009	[33]	http://pubs.rsc.org/en/Content/ArticleLanding/2009/CP/b908209c
83	$[C_6Py][BF_4]$	QCM−Langmuir	145	434	2017	[39]	http://onlinelibrary.wiley.com/doi/10.1002/zaac.201600335/abstract
84	$[^3C_1{}^1C_4Py][BF_4]$	QCM−Langmuir	139	438.2	2017	[39]	http://onlinelibrary.wiley.com/doi/10.1002/zaac.201600335/abstract
85	$[^4C_1{}^1C_4Py][BF_4]$	QCM−Langmuir	140	421.2	2017	[39]	http://onlinelibrary.wiley.com/doi/10.1002/zaac.201600335/abstract
86	$[C_2C_1Im][C_1SO_4]$	QCM	135	442.0	2012	[37]	http://onlinelibrary.wiley.com/doi/10.1002/cphc.201100879/abstract
87	$[C_2C_1Im][C_2SO_4]$	QCM	144	422.0	2012	[37]	http://onlinelibrary.wiley.com/doi/10.1002/cphc.201100879/abstract
87	$[C_2C_1Im][C_2SO_4]$	TPD	139	454	2010	[10]	http://pubs.rsc.org/en/Content/ArticleLanding/2010/CP/c004197a
88	$[C_2C_1Im][C_4SO_4]$	QCM	144	437.9	2012	[37]	http://onlinelibrary.wiley.com/doi/10.1002/cphc.201100879/abstract
89	$[C_2C_1Im][C_8SO_4]$	QCM	157	447.8	2012	[37]	http://onlinelibrary.wiley.com/doi/10.1002/cphc.201100879/abstract
90	$[C_4C_1Im][C_8SO_4]$	TPD	161	510	2012	[38]	http://pubs.rsc.org/en/content/articlelanding/2012/cp/c2cp23705a
91	$[C_4Py][C_1SO_4]$	TPD	144	510	2012	[38]	http://pubs.rsc.org/en/content/articlelanding/2012/cp/c2cp23705a
92	$[C_2C_1Im][TfO]$	QCM	126	412.8	2012	[37]	http://onlinelibrary.wiley.com/doi/10.1002/cphc.201100879/abstract
93	$[C_8C_1Im][TfO]$	TPD	132	495	2007	[11]	http://pubs.rsc.org/en/Content/ArticleLanding/2007/CP/b615137j
94	$[C_2(C_1)_4iU][TfO]$	TPD	110	425	2012	[38]	http://pubs.rsc.org/en/content/articlelanding/2012/cp/c2cp23705a
95	$[C_2C_1Im][PF_6]$	QCM−Langmuir	130	435.2	2016	[40]	http://pubs.acs.org/doi/abs/10.1021/acs.jpcb.6b06081
96	$[C_4C_1Im][PF_6]$	QCM−Langmuir	137	425.7	2016	[40]	http://pubs.acs.org/doi/abs/10.1021/acs.jpcb.6b06081
97	$[C_6C_1Im][PF_6]$	QCM−Langmuir	140	430.7	2016	[40]	http://pubs.acs.org/doi/abs/10.1021/acs.jpcb.6b06081
98	$[C_8C_1Im][PF_6]$	QCM−Langmuir	143	433.7	2016	[40]	http://pubs.acs.org/doi/abs/10.1021/acs.jpcb.6b06081
98	$[C_8C_1Im][PF_6]$	TPD	147	530	2007	[11]	http://pubs.rsc.org/en/Content/ArticleLanding/2007/CP/b615137j
99	$[C_{10}C_1Im][PF_6]$	QCM−Langmuir	149	436.3	2016	[40]	http://pubs.acs.org/doi/abs/10.1021/acs.jpcb.6b06081
100	$[C_2C_1Im][SCN]$	QCM	142	413.2	2012	[37]	http://onlinelibrary.wiley.com/doi/10.1002/cphc.201100879/abstract
100	$[C_2C_1Im][SCN]$	TPD	133	490	2009	[33]	http://pubs.rsc.org/en/Content/ArticleLanding/2009/CP/b908209c

101	$[N_{4,4,4,4}][SCN]$	QCM−Langmuir	151	424.8	2015	[41]	http://link.springer.com/article/10.1007%2Fs10953-015-0316-2
102	$[C_8C_1Im][N(CN)_2]$	TPD	141	520	2009	[33]	http://pubs.rsc.org/en/Content/ArticleLanding/2009/CP/b908209c
103	$[C_4C_1Pyrr][N(CN)_2]$	TPD	142	500	2008	[42]	http://pubs.acs.org/doi/abs/10.1021/jp803238t
104	$[C_2C_1Im][C(CN)_3]$	QCM	126	423.2	2011	[43]	http://pubs.acs.org/doi/abs/10.1021/jp207335m
105	$[C_4C_1Im][C(CN)_3]$	QCM	130<br>130	428.3	2011	[43]	http://pubs.acs.org/doi/abs/10.1021/jp207335m
106	$[C_2C_1Im][B(CN)_4]$	QCM	125	403.9	2012	[37]	http://onlinelibrary.wiley.com/doi/10.1002/cphc.201100879/abstract
107	$[C_4C_1Im][FeCl_4]$	TPD	150	510	2009	[33]	http://pubs.rsc.org/en/Content/ArticleLanding/2009/CP/b908209c
108	$[C_2C_1Im][FAP]$	QCM	118	373.4	2012	[37]	http://onlinelibrary.wiley.com/doi/10.1002/cphc.201100879/abstract
109	$[C_6C_1Im][FAP]$	TPD	131	430	2012	[38]	http://pubs.rsc.org/en/content/articlelanding/2012/cp/c2cp23705a
110	$[C_4C_1Pyrr][FAP]$	TPD	138	450	2009	[33]	http://pubs.rsc.org/en/Content/ArticleLanding/2009/CP/b908209c
111	$[C_2C_1Im][(C_2F_5)_2PO_2]$	TPD	120	400	2012	[38]	http://pubs.rsc.org/en/content/articlelanding/2012/cp/c2cp23705a
112	$[C_2C_1Im][(C_2H_5O)_2PO_2]$	QCM	137	392.9	2012	[37]	http://onlinelibrary.wiley.com/doi/10.1002/cphc.201100879/abstract
113	$[C_2C_1Im][CF_3CO_2]$	QCM	121	384.0	2012	[37]	http://onlinelibrary.wiley.com/doi/10.1002/cphc.201100879/abstract
114	$[C_2C_1Im][TOS]$	QCM	150	460.5	2012	[37]	http://onlinelibrary.wiley.com/doi/10.1002/cphc.201100879/abstract
115	$[C_8C_1Im]Cl$	TPD	151	455	2014	[44]	http://pubs.rsc.org/en/Content/ArticleLanding/2014/CP/C3CP52950A#!divAbstract

116	$[C_8C_1Im]I$	TPD	149	480	2014	[44]	http://pubs.rsc.org/en/Content/ArticleLanding/2014/CP/C3CP52950A#!divAbstract

Table S3. Method to obtain $\Delta_{vap}H_T$, $\Delta_{vap}H_T$, T, year published, literature reference and article DOI.

	Ionic liquid	Method	$\Delta_{vap}H_T$ / kJ mol^{-1}	T / K	$\Delta_{vap}H_{298}$ (variable $\Delta_g^l C_p$) / kJ mol^{-1}	$\Delta_g^l C_p$ / J K^{-1} mol^{-1}	$\Delta_{vap}H_{298}$ (constant $\Delta_g^l C_p$) / kJ mol^{-1}	$\Delta_{vap}H_{450}$ (variable $\Delta_g^l C_p$) / kJ mol^{-1}	$\Delta_{vap}H_{450}$ (constant $\Delta_g^l C_p$) / kJ mol^{-1}
1	$[C_1C_1Im][NTf_2]$	QCM	120	381.6	126	–73	128	115	113
1	$[C_1C_1Im][NTf_2]$	KEQCM	117	484.65	136	–106	135	120	120
1	$[C_1C_1Im][NTf_2]$	TGA	104	590	126	–73	134	115	118
			114		129		132	117	117
2	$[C_2C_1Im][NTf_2]$	QCM	119	378	123	–56	127	115	111
2	$[C_2C_1Im][NTf_2]$	TPD	121	398	130	–94	131	116	116
2	$[C_2C_1Im][NTf_2]$	TPD	122	430	134	–94	135	120	120
2	$[C_2C_1Im][NTf_2]$	KEMS	121	459	130	–56	137	122	122
2	$[C_2C_1Im][NTf_2]$	Knudsen mass loss	119	463	135	–100	135	120	120
2	$[C_2C_1Im][NTf_2]$	KEQCM	115	464.36	133	–112	131	116	116
2	$[C_2C_1Im][NTf_2]$	TGA	121	495.5			140		125
2	$[C_2C_1Im][NTf_2]$	transpiration	115	516.2	137	–100	137	122	122
2	$[C_2C_1Im][NTf_2]$	TGA	111	520.2	123	–56	133	114	118
2	$[C_2C_1Im][NTf_2]$	UV ab	112	573			139		124
2	$[C_2C_1Im][NTf_2]$	Calorimetry (He)	108	625	127	–56	141	118	126
2	$[C_2C_1Im][NTf_2]$	Calorimetry (N2)	110	625	128	–56	143	120	128
			116		130		136	118	121
3	$[C_3C_1Im][NTf_2]$	QCM	121	380.3	128	–76	130	116	114
3	$[C_3C_1Im][NTf_2]$	KEQCM	113	473.04	134	–117	131	116	116
3	$[C_3C_1Im][NTf_2]$	TGA	109	546.2	128	–76	134	116	118
			115		130		131	116	116
4	$[C_4C_1Im][NTf_2]$	QCM	124	378	130	–67	132	120	117
4	$[C_4C_1Im][NTf_2]$	KEML	118	427	127	–66.8	131	117	116
4	$[C_4C_1Im][NTf_2]$	TPD	121	440	134	–94	135	120	120
4	$[C_4C_1Im][NTf_2]$	KEQCM	118	475.01	139	–121	135	121	120
4	$[C_4C_1Im][NTf_2]$	Knudsen mass loss	118	477.6	136	–100	136	121	121
4	$[C_4C_1Im][NTf_2]$	TGA	119	495.5			138		123
4	$[C_4C_1Im][NTf_2]$	TGA	114	541.8	130	–67	138	120	123

4	$[C_4C_1Im][NTf_2]$	UV ab	114	553			140		124
4	$[C_4C_1Im][NTf_2]$	TGA	108	600			138		123
			117		133		136	120	121
5	$[C_5C_1Im][NTf_2]$	QCM	128	381.7	136	–96	136	121	121
5	$[C_5C_1Im][NTf_2]$	KEQCM	123	474.99	145	–127	141	126	125
5	$[C_5C_1Im][NTf_2]$	TGA	111	560.8	136	–96	137	121	122
			120		139		138	123	123
6	$[C_6C_1Im][NTf_2]$	QCM	132	383	139	–86	140	126	125
6	$[C_6C_1Im][NTf_2]$	TPD	125	445	139	–94	140	125	125
6	$[C_6C_1Im][NTf_2]$	Knudsen mass loss	123	461.8	140	–100	140	125	125
6	$[C_6C_1Im][NTf_2]$	KEQCM	126	477.35	150	–134	144	130	129
6	$[C_6C_1Im][NTf_2]$	TGA	124	503			145		129
6	$[C_6C_1Im][NTf_2]$	TGA	118	541.8	139	–86	142	126	127
			125		141		142	126	127
7	$[C_7C_1Im][NTf_2]$	QCM	134	385.3	143	–114	142	126	127
7	$[C_7C_1Im][NTf_2]$	KEQCM	129	478.92	154	–138	147	133	132
7	$[C_7C_1Im][NTf_2]$	TGA	114	553.7	143	–114	140	126	125
			126		147		143	128	128
8	$[C_8C_1Im][NTf_2]$	QCM	137	387	145	–92	146	131	131
8	$[C_8C_1Im][NTf_2]$	TPD	135	450	149	–94	150	135	135
8	$[C_8C_1Im][NTf_2]$	Knudsen mass loss	132	475.2	150	–100	150	135	135
8	$[C_8C_1Im][NTf_2]$	KEQCM	129	484.1	155	–143	147	133	132
8	$[C_8C_1Im][NTf_2]$	TGA	132	503			153		138
8	$[C_8C_1Im][NTf_2]$	TGA	123	541.8	145	–92	147	131	132
			131		149		149	133	134
9	$[C_{10}C_1Im][NTf_2]$	QCM	143	394.6	156	–136	152	135	137
9	$[C_{10}C_1Im][NTf_2]$	KEQCM	136	487.38	165	–154	155	142	140
9	$[C_{10}C_1Im][NTf_2]$	TGA	134	510			155		140
9	$[C_{10}C_1Im][NTf_2]$	TGA	125	522	156	–136	148	135	132
			134		159		152	137	137
10	$[C_{12}C_1Im][NTf_2]$	QCM	147	408.4	165	–167	158	140	143

10	$[C_{12}C_1Im][NTf_2]$	KEQCM	140	486.85	172	–165	159	146	144
10	$[C_{12}C_1Im][NTf_2]$	TGA	126	533.5	165	–167	150	140	134
			138		167		156	142	140
11	$[C_{14}C_1Im][NTf_2]$	QCM	153	416.2	172	–161	164	147	149
11	$[C_{14}C_1Im][NTf_2]$	KEQCM	141	513.06	178	–176	162	152	147
11	$[C_{14}C_1Im][NTf_2]$	TGA	133	541.8	172	–161	157	147	142
			142		174		161	149	146
12	$[C_{16}C_1Im][NTf_2]$	QCM	156	424.8	178	–170	169	152	154
12	$[C_{16}C_1Im][NTf_2]$	KEQCM	147	520.57	189	–187	169	160	154
12	$[C_{16}C_1Im][NTf_2]$	TGA	136	541.8	178	–170	161	152	146
			147		181		166	155	151
13	$[C_{18}C_1Im][NTf_2]$	QCM	162	430.3	183	–157	176	159	160
13	$[C_{18}C_1Im][NTf_2]$	TGA	139	582.5	183	–157	167	159	152
			150		183		171	159	156
14	$[C_1C_1C_1Im][NTf_2]$	QCM	122	396	132	–100	132	117	117
15	$[C_3C_1C_1Im][NTf_2]$	KEMS	124	464	135	–67	140	125	125
15	$[C_3C_1C_1Im][NTf_2]$	KEQCM	117	485.57	140	–121	136	122	121
15	$[C_3C_1C_1Im][NTf_2]$	TGA	130	503			151		135
			124		137		142	123	127
15	$[C_4C_1C_1Im][NTf_2]$	KEQCM	121	483.09	145	–127	140	125	124
16	$[C_4C_1C_1Im][NTf_2]$	QCM	130	503	151	–100	151	135	135
			126		148		145	130	130
17	$[C_3(C_1Im)_2][NTf_2]_2$	TPD	158	545	190	–130	183	170	168
18	$[C_1C_1C_1BIm][NTf_2]$	QCM	134	397.5	140	–62.9	143	130	128
18	$[C_1C_1C_1BIm][NTf_2]$	TGA	122	581.8	140	–62.9	150	130	135
			128		140		147	130	132
19	$[Me(EG)_1C_1Im][NTf_2]$	QCM	118	365.5	122	–48	125	114	110
19	$[Me(EG)_1C_1Im][NTf_2]$	TGA	110	542.4	122	–48	134	114	119
			114		122		130	114	115

20	$[Me(EG)_2C_1Im][NTf_2]$	QCM	127	378.3	133	–79	135	121	119
20	$[Me(EG)_2C_1Im][NTf_2]$	TGA	114	543.9	133	–79	138	121	123
			120		133		136	121	121
21	$[Me(EG)_3C_1Im][NTf_2]$	QCM	134	388.2	142	–98	143	128	127
21	$[Me(EG)_3C_1Im][NTf_2]$	TGA	118	551	143	–98	143	128	128
			126		143		143	128	128
22	$[Me(EG)_1C_1C_1Im][NTf_2]$	QCM	123	381.1	133	–124	131	114	116
22	$[Me(EG)_1C_1C_1Im][NTf_2]$	TGA	103	543.5	133	–124	127	114	112
			113		133		129	114	114
23	$[Me(EG)_2C_1C_1Im][NTf_2]$	QCM	129	390.9	140	–126	138	121	123
23	$[Me(EG)_2C_1C_1Im][NTf_2]$	TGA	110	543.9	140	–126	134	121	119
			119		140		136	121	121
24	$[Me(EG)_3C_1C_1Im][NTf_2]$	QCM	134	400.9	148	–130	145	128	129
25	$[(C_2F_5C_2H_4)C_1Im][NTf_2]$	QCM−Langmuir	130	370.1	137.9	–109	137	121	122
25	$[(C_2F_5C_2H_4)C_1Im][NTf_2]$	TGA	114	527.7	139	–109	137	122	122
			122		138		137	122	122
26	$[(C_4F_9C_2H_4)C_1Im][NTf_2]$	QCM−Langmuir	138	371.1	146.1	–116	145	128	130
26	$[(C_4F_9C_2H_4)C_1Im][NTf_2]$	TGA	116	532.48	143	–116	140	126	124
			127		145		142	127	127
27	$[(C_6F_{13}C_2H_4)C_1Im][NTf_2]$	QCM−Langmuir	143	376.2	152.1	–123	150	133	135
27	$[(C_6F_{13}C_2H_4)C_1Im][NTf_2]$	TGA	126	532.3	155	–123	149	136	134
			134		153		150	135	135
28	$[(C_8F_{17}C_2H_4)C_1Im][NTf_2]$	QCM−Langmuir	148	381.4	158.9	–130	156	139	141
28	$[(C_8F_{17}C_2H_4)C_1Im][NTf_2]$	TGA	128	531.8	158	–130	151	138	136
			138		159		154	139	139
29	$[i–C_3C_1Im][NTf_2]$	QCM−Langmuir	123	366.3	128	–69.1	130	118	115
30	$[i–C_4C_1Im][NTf_2]$	QCM−Langmuir	126	368.7	131	–76.1	133	120	118
31	$[s–C_4C_1Im][NTf_2]$	QCM−Langmuir	123	368.6	128	–76.1	130	117	115

32	[cyclo–C_3–CH_2–C_1Im][NTf_2]	QCM−Langmuir	127	373.5	132	–71.5	134	121	119
33	[cyclo–C_5C_1Im][NTf_2]	QCM−Langmuir	127	373.7	133	–77.1	135	122	120
34	[cyclo–C_6–CH_2–C_1Im][NTf_2]	QCM−Langmuir	134	383.6	142	–91.7	143	128	127
35	[Phenyl–Im][NTf_2] 1	QCM−Langmuir	132	384.8	138	–65	141	128	126
36	[Phenyl–Im][NTf_2] 2	QCM−Langmuir	134	373.3	139	–65	142	129	127
37	[Phenyl–Im][NTf_2] 3	QCM−Langmuir	142	381	148	–65	151	138	135
38	[Phenyl–Im][NTf_2] 4	QCM−Langmuir	137	387.3	142	–65	145	132	130
39	[Phenyl–Im][NTf_2] 5	QCM−Langmuir	142	396.1	148	–65	152	138	136
40	[C_2C_2Im][NTf_2]	KEQCM	109	467.61	129	–117	126	111	111
41	[C_3C_3Im][NTf_2]	KEQCM	114	471.6	136	–127	131	117	116
42	[C_4C_4Im][NTf_2]	KEQCM	117	471.58	141	–138	134	120	119
43	[C_5C_5Im][NTf_2]	KEQCM	124	479.54	151	–149	142	128	127
44	[C_6C_6Im][NTf_2]	KEQCM	138	479.6	167	–160	157	143	141
45	[C_3C_2Im][NTf_2]	KEQCM	115	476.6	136	–121	132	118	117
46	[C_7C_7Im][NTf_2]	KEQCM	131	493.39	164	–171	150	138	135
47	[C_8C_8Im][NTf_2]	KEQCM	126	494.33	162	–181	146	134	130
48	[C_9C_9Im][NTf_2]	KEQCM	129	508.1	169	–192	150	140	134
49	[$C_{10}C_{10}$Im][NTf_2]	KEQCM	125	502.37	167	–203	146	136	131
50	[C_3C_1Pyrr][NTf_2]	QCM	131	415.7	140	–75	143	129	128
50	[C_3C_1Pyrr][NTf_2]	TGA	126	554.2	145	–75	151	134	136
			129		143		147	131	132
51	[C_4C_1Pyrr][NTf_2]	QCM	134	415.8	144	–85	145	131	130
51	[C_4C_1Pyrr][NTf_2]	TPD	136	470	152	–94	153	138	138
51	[C_4C_1Pyrr][NTf_2]	TGA	124	554.3	146	–85	150	133	135
			131		147		150	134	134
52	[C_5C_1Pyrr][NTf_2]	QCM	135	405.7	145	–93	146	131	131

53	[C$_6$C$_1$Pyrr][NTf$_2$]	QCM	138	405.9	149	–102	149	134	134
53	[C$_6$C$_1$Pyrr][NTf$_2$]	TPD	141	460	156	–94	157	142	142
53	[C$_6$C$_1$Pyrr][NTf$_2$]	TGA	124	554.7	150	–102	150	135	135
			134		152		152	137	137
54	[C$_7$C$_1$Pyrr][NTf$_2$]	QCM	142	408.3	154	–109	153	137	137
54	[C$_7$C$_1$Pyrr][NTf$_2$]	TGA	131	554.9	159	–109	157	142	141
			136		156		155	140	139
55	[C$_8$C$_1$Pyrr][NTf$_2$]	QCM	144	410.9	158	–118	156	140	140
55	[C$_8$C$_1$Pyrr][NTf$_2$]	TPD	145	470	161	–94	162	147	147
55	[C$_8$C$_1$Pyrr][NTf$_2$]	TGA	130	562.1	161	–118	157	143	141
			140		160		158	143	143
56	[C$_{10}$C$_1$Pyrr][NTf$_2$]	QCM	149	418.4	165	–134	161	144	146
56	[C$_{10}$C$_1$Pyrr][NTf$_2$]	TGA	135	562.3	171	–134	162	150	146
			142		168		161	147	146
57	[C$_2$Py][NTf$_2$]	QCM	125	400.6	132	–61	136	122	120
57	[C$_2$Py][NTf$_2$]	KEQCM	120	498.58	140	–100	140	125	125
			123		136		138	124	123
58	[C$_3$Py][NTf$_2$]	QCM	128	398.2	135	–66	138	125	123
58	[C$_3$Py][NTf$_2$]	KEQCM	124	504.5	145	–100	145	130	130
			126		140		141	127	126
59	[C$_4$Py][NTf$_2$]	QCM	131	399.5	138	–70	141	128	126
59	[C$_4$Py][NTf$_2$]	KEQCM	122	506.82	143	–100	143	128	128
59	[C$_4$Py][NTf$_2$]	UV ab	120	553			145		130
			124		140		143	128	128
60	[C$_5$Py][NTf2]	QCM	134	400.6	142	–73	144	131	129
61	[C$_6$Py][NTf$_2$]	QCM	137	405.7	146	–77	148	134	133
61	[C$_6$Py][NTf$_2$]	TPD	139	440	152	–94	153	138	138
			138		149		151	136	135

62	$[^{2}C_2{}^{1}C_2Py][NTf_2]$	KEQCM	125	508.11	146	–100	146	130	130
63	$[^{2}C_3{}^{1}C_2Py][NTf_2]$	KEQCM	121	510.59	142	–100	142	127	127
64	$[^{2}C_4{}^{1}C_2Py][NTf_2]$	KEQCM	122	503.12	143	–100	143	128	128
65	$[^{2}C_5{}^{1}C_2Py][NTf_2]$	KEQCM	127	510.63	149	–100	149	133	133
66	$[^{2}C_6{}^{1}C_2Py][NTf_2]$	KEQCM	128	505.65	149	–100	149	134	134
67	$[^{2}C_7{}^{1}C_2Py][NTf_2]$	KEQCM	131	508.27	152	–100	152	137	137
68	$[^{2}C_8{}^{1}C_2Py][NTf_2]$	KEQCM	138	505.73	159	–100	159	144	144
69	$[^{2}C_9{}^{1}C_2Py][NTf_2]$	KEQCM	140	523.03	162	–100	162	147	147
70	$[^{2}C_{10}{}^{1}C_2Py][NTf_2]$	KEQCM	144	520.5	167	–100	167	152	152
71	$[C_2C_1Im][NPf_2]$	TGA	115	503			136		121
71	$[C_2C_1Im][NPf_2]$	UV ab	110	553			136		121
			113				136		121
72	$[C_4C_1Im][NPf_2]$	TGA	114	503			135		120
72	$[C_4C_1Im][NPf_2]$	UV ab	114	553			139		124
			114				137		122
73	$[C_6C_1Im][NPf_2]$	TGA	118	503			139		124
74	$[C_6C_1Im][NPf_2]$	TGA	125	499.3			145		130
75	$[C_{10}C_1Im][NPf_2]$	TGA	127	510			149		133
76	$[C_3C_1C_1Im][NPf_2]$	TGA	122	503			142		127
77	$[C_2C_1Im][BF_4]$	QCM	122	431.6	136	–100	136	120	120
77	$[C_2C_1Im][BF_4]$	TPD	128	515	148	–94	150	134	135
			125		142		143	127	127
78	$[C_4C_1Im][BF_4]$	TPD	134	500	153	–94	154	139	139
79	$[C_8C_1Im][BF_4]$	TPD	141	520	162	–94	163	148	148
80	$[P_{6,6,6,14}][BF_4]$	TPD	180	490	198	–94	199	184	184
81	$[C_2Py][BF_4]$	QCM−Langmuir	136	459.4	146	–61	152	136	137

82	$[C_4Py][BF_4]$	QCM−Langmuir	140	433.8	150	–70	154	139	139
82	$[C_4Py][BF_4]$	TPD	146	510	166	–94	167	152	152
			143		158		161	145	145
83	$[C_6Py][BF_4]$	QCM−Langmuir	145	434	155	–77	158	144	143
84	$[^3C_1{}^1C_4Py][BF_4]$	QCM−Langmuir	139	438.2	150	–73	153	138	138
85	$[^4C_1{}^1C_4Py][BF_4]$	QCM−Langmuir	140	421.2	149	–73	152	138	137
86	$[C_2C_1Im][C_1SO_4]$	QCM	135	442.0	150	–100	150	134	134
87	$[C_2C_1Im][C_2SO_4]$	QCM	144	422.0	156	–100	156	141	141
87	$[C_2C_1Im][C_2SO_4]$	TPD	139	454	154	–94	155	139	139
			141		155		155	140	140
88	$[C_2C_1Im][C_4SO_4]$	QCM	144	437.9	158	–100	158	143	143
89	$[C_2C_1Im][C_8SO_4]$	QCM	157	447.8	172	–100	172	157	157
90	$[C_4C_1Im][C_8SO_4]$	TPD	161	510	181	–94	182	167	167
91	$[C_4Py][C_1SO_4]$	TPD	144	510	164	–94	165	150	150
92	$[C_2C_1Im][TfO]$	QCM	126	412.8	138	–100	138	123	123
93	$[C_8C_1Im][TfO]$	TPD	132	495	151	–94	152	136	137
94	$[C_2(C_1)_4iU][TfO]$	TPD	110	425	122	–94	123	108	108
95	$[C_2C_1Im][PF_6]$	QCM−Langmuir	130	435.2	140	–74	144	129	128
96	$[C_4C_1Im][PF_6]$	QCM−Langmuir	137	425.7	147	–74	150	135	135
97	$[C_6C_1Im][PF_6]$	QCM−Langmuir	140	430.7	151	–81	153	138	138
98	$[C_8C_1Im][PF_6]$	QCM−Langmuir	143	433.7	155	–85	157	142	142
98	$[C_8C_1Im][PF_6]$	TPD	147	530	169	–94	170	155	155
			145		162		164	148	148

99	[$C_{10}C_1$Im][PF_6]	QCM−Langmuir	149	436.3	161	–93	162	147	147
100	[C_2C_1Im][SCN]	QCM	142	413.2	154	–100	154	139	139
100	[C_2C_1Im][SCN]	TPD	133	490	151	–94	152	137	137
			138		152		153	138	138
101	[$N_{4,4,4,4}$][SCN]	QCM−Langmuir	151	424.8	164	–100	164	149	149
102	[C_8C_1Im][$N(CN)_2$]	TPD	141	520	162	–94	163	148	148
103	[C_4C_1Pyrr][$N(CN)_2$]	TPD	142	500	161	–94	162	147	147
104	[C_2C_1Im][$C(CN)_3$]	QCM	126	423.2	139	–100	139	123	123
105	[C_4C_1Im][$C(CN)_3$]	QCM	130	428.3	143	–100	143	128	128
			130		143		143	128	128
106	[C_2C_1Im][$B(CN)_4$]	QCM	125	403.9	136	–100	136	120	120
107	[C_4C_1Im][$FeCl_4$]	TPD	150	510	170	–94	171	156	156
108	[C_2C_1Im][FAP]	QCM	118	373.4	126	–100	126	111	111
109	[C_6C_1Im][FAP]	TPD	131	430	143	–94	144	129	129
110	[C_4C_1Pyrr][FAP]	TPD	138	450	152	–94	153	138	138
111	[C_2C_1Im][$(C_2F_5)_2PO_2$]	TPD	120	400	130	–94	130	115	115
112	[C_2C_1Im][$(C_2H_5O)_2PO_2$]	QCM	137	392.9	146	–100	146	131	131
113	[C_2C_1Im][CF_3CO_2]	QCM	121	384.0	129	–100	129	114	114

114	$[C_2C_1Im][TOS]$	QCM	150	460.5	166	–100	166	151	151
115	$[C_8C_1Im]Cl$	TPD	151	455	166	–94	167	151	152
116	$[C_8C_1Im]I$	TPD	149	480	166	–94	167	152	152

Table S4. $\Delta_{vap}H_T$, T, $\Delta_{vap}H_{298}$ (using $\Delta^g{}_lC_p$ data provided in each article), $\Delta^g{}_lC_p$, $\Delta_{vap}H_{298}$ (using $\Delta^g{}_lC_p$ = –100 J K^{-1} mol^{-1}), $\Delta_{vap}H_{450}$ (using $\Delta^g{}_lC_p$ data provided in each article) and $\Delta_{vap}H_{450}$ (using $\Delta^g{}_lC_p$ = –100 J K^{-1} mol^{-1}). $\Delta_{vap}H_{298}$ values were determined for each IL by taking the average of all reliable individual $\Delta_{vap}H_{298}$ literature values (how these individual $\Delta_{vap}H_{298}$ values were chosen is explained in the main text, Section 4.4). The various average $\Delta_{vap}H$ values are given in boxes.

	Molecular liquid	$\Delta_{vap}H_{298}$ / kJ mol^{-1}	V_m / cm^3 mol^{-1}	V_{mol} / nm^3	γ / mN m^{-1}	G / J cm^{-3}	$ced_{ML,298}$ / J cm^{-3}
1	water	44	18	0.030	71.8	231.0	2293
2	formamide	61	40	0.066	58.2	143.8	1456
3	glycerol	86	73	0.122	63.3	127.8	1135
4	ethanolamine	66	60	0.100	48.3	104.0	1055
5	triethanolamine	102	133	0.221	45.2	74.7	745
6	ethanol	42	59	0.098	21.9	47.6	679
7	acetonitrile	33	53	0.088	28.3	63.7	581
8	acetone	31	74	0.123	22.7	45.7	385
9	THF	32	82	0.136	26.4	51.4	359
10	toluene	38	107	0.178	27.9	49.6	332
11	*n*–hexadecane	81	294	0.489	27.1	34.4	267
12	diethyl ether	27	105	0.174	16.5	29.6	236
13	*n*–hexane	31	132	0.219	17.9	29.7	220
14	squalane	105	525	0.873	27.8	29.1	195

Table S5. $\Delta_{vap}H_{298}$, V_m, V_{mol}, γ, G and $ced_{ML,298}$ data for select molecular liquids, data taken from ref. [45].

Section S4. Further figures showing correlations

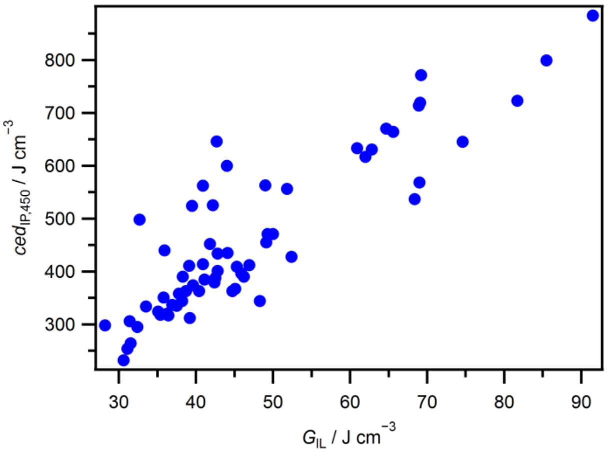

Figure S1. $ced_{IP,450}$ for ILs (extrapolated to T = 450 K using constant $\Delta^g_l C_p$ = –100 J K^{-1} mol^{-1}) *versus* G_{IL}.

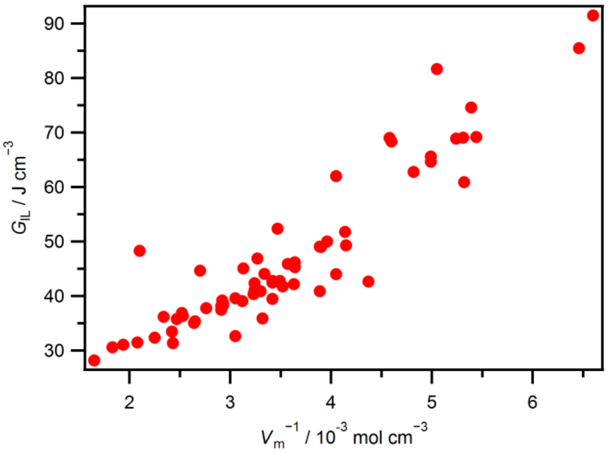

Figure S2. G_{IL} for ILs *versus* V_m^{-1}.

Section S5. References

1 Lui, M. Y., Crowhurst, L., Hallett, J. P., Hunt, P. A., Niedermeyer, H., Welton, T. 2011 Salts dissolved in salts: ionic liquid mixtures. *Chemical Science*. **2**, 1491-1496. (10.1039/c1sc00227a)

2 Malberg, F., Pensado, A. S., Kirchner, B. 2012 The bulk and the gas phase of 1-ethyl-3-methylimidazolium ethylsulfate: dispersion interaction makes the difference. *Phys. Chem. Chem. Phys.* **14**, 12079-12082. (10.1039/c2cp41878a)

3 Malberg, F., Brehm, M., Hollóczki, O., Pensado, A. S., Kirchner, B. 2013 Understanding the evaporation of ionic liquids using the example of 1-ethyl-3-methylimidazolium ethylsulfate. *Phys. Chem. Chem. Phys.* **15**, 18424-18436. (10.1039/c3cp52966e)

4 Malberg, F., Hollóczki, O., Thomas, M., Kirchner, B. 2015 En route formation of ion pairs at the ionic liquid-vacuum interface. *Struct. Chem.* **26**, 1343-1349. (10.1007/s11224-015-0662-0)

5 Hessey, S. G., Jones, R. G. 2013 On the evaporation, bonding, and adsorbate capture of an ionic liquid on Au(111). *Chemical Science*. **4**, 2519-2529. (10.1039/c3sc00072a)

6 Villar-Garcia, I. J., Fearn, S., De Gregorio, G. F., Ismail, N. L., Gschwend, F. J. V., McIntosh, A. J. S., Lovelock, K. R. J. 2014 The ionic liquid-vacuum outer atomic surface: a low-energy ion scattering study. *Chemical Science*. **5**, 4404-4418. (10.1039/c4sc00640b)

7 Booth, R. S., Annesley, C. J., Young, J. W., Vogelhuber, K. M., Boatz, J. A., Stearns, J. A. 2016 Identification of multiple conformers of the ionic liquid emim tf2n in the gas phase using IR/UV action spectroscopy. *Phys. Chem. Chem. Phys.* **18**, 17037-17043. (10.1039/c6cp02657e)

8 Verevkin, S. P., Zaitsau, D. H., Emel'yanenko, V. N., Yermalayeu, A. V., Schick, C., Liu, H. J., Maginn, E. J., Bulut, S., Krossing, I., Kalb, R. 2013 Making Sense of Enthalpy of Vaporization Trends for Ionic Liquids: New Experimental and Simulation Data Show a Simple Linear Relationship and Help Reconcile Previous Data. *J. Phys. Chem. B*. **117**, 6473-6486. (10.1021/jp311429r)

9 Rocha, M. A. A., Ribeiro, F. M. S., Schröder, B., Coutinho, J. A. P., Santos, L. M. N. B. F. 2014 Volatility study of C(1)C(1)im NTf2 and C(2)C(3)im NTf2 ionic liquids. *J. Chem. Thermodyn.* **68**, 317-321. (10.1016/j.jct.2013.09.020)

10 Lovelock, K. R. J., Deyko, A., Licence, P., Jones, R. G. 2010 Vaporisation of an ionic liquid near room temperature. *Phys. Chem. Chem. Phys.* **12**, 8893-8901. (10.1039/c004197a)

11 Armstrong, J. P., Hurst, C., Jones, R. G., Licence, P., Lovelock, K. R. J., Satterley, C. J., Villar-Garcia, I. J. 2007 Vapourisation of ionic liquids. *Phys. Chem. Chem. Phys.* **9**, 982-990. (10.1039/B615137J)

12 Chilingarov, N. S., Medvedev, A. A., Deyko, G. S., Kustov, L. M., Chernikova, E. A., Glukhov, L. M., Markov, V. Y., Ioffe, I. Y. N., Senyavin, V. M., Polyakova, M. V.*, et al.* 2015 Mass spectrometric studies of 1-ethyl-3-methylimidazolium and 1-propyl-2,3-dimethylimidazolium bis(trifluoromethyl)-sulfonylimides. *Rapid Commun. Mass Spectrom.* **29**, 1227-1232. (10.1002/rcm.7214)

13 Zaitsau, D. H., Kabo, G. J., Strechan, A. A., Paulechka, Y. U., Tschersich, A., Verevkin, S. P., Heintz, A. 2006 Experimental vapor pressures of 1-alkyl-3-methylimidazolium bis(trifluoromethylsulfonyl) imides and a correlation scheme for estimation of vaporization enthalpies of ionic liquids. *J. Phys. Chem. A*. **110**, 7303-7306. (10.1021/jp060896f)

14 Rocha, M. A. A., Lima, C. F. R. A. C., Gomes, L. R., Schröder, B., Coutinho, J. A. P., Marrucho, I. M., Esperança, J. M. S. S., Rebelo, L. P. N., Shimizu, K., Canongia Lopes, J. N.*, et al.* 2011 High-Accuracy Vapor Pressure Data of the Extended [C(n)C(1)im][Ntf(2)] Ionic Liquid Series: Trend Changes and Structural Shifts. *J. Phys. Chem. B*. **115**, 10919-10926. (10.1021/jp2049316)

15 Luo, H. M., Baker, G. A., Dai, S. 2008 Isothermogravimetric determination of the enthalpies of vaporization of 1-alkyl-3-methylimidazolium ionic liquids. *J. Phys. Chem. B*. **112**, 10077-10081. (10.1021/jp805340f)
16 Emel'yanenko, V. N., Verevkin, S. P., Heintz, A. 2007 The gaseous enthalpy of formation of the ionic liquid 1-butyl-3-methylimidazolium dicyanamide from combustion calorimetry, vapor pressure measurements, and ab initio calculations. *J. Am. Chem. Soc.* **129**, 3930-3937. (10.1021/ja0679174)
17 Wang, C. M., Luo, H. M., Li, H. R., Dai, S. 2010 Direct UV-spectroscopic measurement of selected ionic-liquid vapors. *Phys. Chem. Chem. Phys.* **12**, 7246-7250. (10.1039/c001101k)
18 Ahrenberg, M., Brinckmann, M., Schmelzer, J. W. P., Beck, M., Schmidt, C., Kessler, O., Kragl, U., Verevkin, S. P., Schick, C. 2014 Determination of volatility of ionic liquids at the nanoscale by means of ultra-fast scanning calorimetry. *Phys. Chem. Chem. Phys.* **16**, 2971-2980. (10.1039/c3cp54325k)
19 Brunetti, B., Ciccioli, A., Gigli, G., Lapi, A., Misceo, N., Tanzi, L., Ciprioti, S. V. 2014 Vaporization of the prototypical ionic liquid BMImNTf(2) under equilibrium conditions: a multitechnique study. *Phys. Chem. Chem. Phys.* **16**, 15653-15661. (10.1039/c4cp01673d)
20 Seeberger, A., Andresen, A. K., Jess, A. 2009 Prediction of long-term stability of ionic liquids at elevated temperatures by means of non-isothermal thermogravimetrical analysis. *Phys. Chem. Chem. Phys.* **11**, 9375-9381. (10.1039/B909624H)
21 Paulechka, E., Blokhin, A. V., Rodrigues, A. S. M. C., Rocha, M. A. A., Santos, L. M. N. B. F. 2016 Thermodynamics of long-chain 1-alkyl-3-methylimidazolium bis(trifluoromethanesulfonyl)imide ionic liquids. *J. Chem. Thermodyn.* **97**, 331-340. (10.1016/j.jct.2016.02.009)
22 Fumino, K., Peppel, T., Geppert-Rybczynska, M., Zaitsau, D. H., Lehmann, J. K., Verevkin, S. P., Köckerling, M., Ludwig, R. 2011 The influence of hydrogen bonding on the physical properties of ionic liquids. *Phys. Chem. Chem. Phys.* **13**, 14064-14075. (10.1039/c1cp20732f)
23 Rodrigues, A. S. M. C., Lima, C. F. R. A. C., Coutinho, J. A. P., Santos, L. M. N. B. F. 2017 Nature of the C2-methylation effect on the properties of imidazolium ionic liquids. *Phys. Chem. Chem. Phys.* **19**, 5326-5332. (10.1039/c6cp08451f)
24 Lovelock, K. R. J., Deyko, A., Corfield, J. A., Gooden, P. N., Licence, P., Jones, R. G. 2009 Vaporisation of a Dicationic Ionic Liquid. *ChemPhysChem*. **10**, 337-340. (10.1002/cphc.200800690)
25 Zaitsau, D. H., Yermalayeu, A. V., Emel'yanenko, V. N., Schick, C., Verevkin, S. P., Samarov, A. A., Schlenk, S., Wasserscheid, P. 2013 Structure-Property Relations in Ionic Liquids: 1,2,3-Trimethyl-imidazolium and 1,2,3-Trimethyl-benzimidazolium bis-(trifluorsulfonyl)imide. *Z. Phys. Chemie-Int. J. Res. Phys. Chem. Chem. Phys.* **227**, 205-215. (10.1524/zpch.2013.0312)
26 Zaitsau, D. H., Yermalayeu, A. V., Verevkin, S. P., Bara, J. E., Stanton, A. D. 2013 Structure-Property Relationships in Ionic Liquids: A Study of the Influence of N(1) Ether and C(2) Methyl Substituents on the Vaporization Enthalpies of Innidazolium-Based Ionic Liquids. *Ind. Eng. Chem. Res.* **52**, 16615-16621. (10.1021/ie402664c)
27 Zaitsau, D. H., Yermalayeu, A. V., Verevkin, S. P., Bara, J. E., Wallace, D. A. 2015 Structure-property relationships in ionic liquids: Chain length dependence of the vaporization enthalpies of imidazolium-based ionic liquids with fluorinated substituents. *Thermochim. Acta*. **622**, 38-43. (10.1016/j.tca.2015.04.021)
28 Zaitsau, D. H., Varfolomeev, M. A., Verevkin, S. P., Stanton, A. D., Hindman, M. S., Bara, J. E. 2016 Structure-property relationships in ionic liquids: Influence of branched and cyclic groups on vaporization enthalpies of imidazolium-based ILs. *J. Chem. Thermodyn.* **93**, 151-156. (10.1016/j.jct.2015.09.033)
29 Zaitsau, D. H., Kaliner, M., Lerch, S., Strassner, T., Emel'yanenko, V. N., Verevkin, S. P. 2017 Thermochemical Properties of Tunable Aryl Alkyl Ionic Liquids (TAAILs) based on Phenyl-1H-imidazoles. *Z. Anorg. Allg. Chem.* **643**, 114-119. (10.1002/zaac.201600333)

30 Rocha, M. A. A., Coutinho, J. A. P., Santos, L. M. N. B. F. 2012 Cation Symmetry effect on the Volatility of Ionic Liquids. *J. Phys. Chem. B*. **116**, 10922-10927. (10.1021/jp306937f)
31 Rocha, M. A. A., Coutinho, J. A. P., Santos, L. M. N. B. F. 2014 Vapor pressures of 1,3-dialkylimidazolium bis(trifluoromethylsulfonyl)imide ionic liquids with long alkyl chains. *J. Chem. Phys.* **141**, 8. (10.1063/1.4896704)
32 Zaitsau, D. H., Yermalayeu, A. V., Emel'yanenko, V. N., Heintz, A., Verevkin, S. P., Schick, C., Berdzinski, S., Strehmel, V. 2014 Structure-property relationships in ILs: Vaporization enthalpies of pyrrolidinium based ionic liquids. *J. Mol. Liq.* **192**, 171-176. (10.1016/j.molliq.2013.07.018)
33 Deyko, A., Lovelock, K. R. J., Corfield, J. A., Taylor, A. W., Gooden, P. N., Villar-Garcia, I. J., Licence, P., Jones, R. G., Krasovskiy, V. G., Chernikova, E. A.*, et al.* 2009 Measuring and predicting Delta H-vap(298) values of ionic liquids. *Phys. Chem. Chem. Phys.* **11**, 8544-8555. (10.1039/b908209c)
34 Zaitsau, D. H., Yermalayeu, A. V., Emel'yanenko, V. N., Verevkin, S. P., Welz-Biermann, U., Schubert, T. 2012 Structure-property relationships in ILs: A study of the alkyl chain length dependence in vaporisation enthalpies of pyridinium based ionic liquids. *Sci. China-Chem.* **55**, 1525-1531. (10.1007/s11426-012-4662-2)
35 Rocha, M. A. A., Santos, L. M. N. B. F. 2013 First volatility study of the 1-alkylpyridinium based ionic liquids by Knudsen effusion. *Chem. Phys. Lett.* **585**, 59-62. (10.1016/j.cplett.2013.08.095)
36 Vilas, M., Rocha, M. A. A., Fernandes, A. M., Tojo, E., Santos, L. M. N. B. F. 2015 Novel 2-alkyl-1-ethylpyridinium ionic liquids: synthesis, dissociation energies and volatility. *Phys. Chem. Chem. Phys.* **17**, 2560-2572. (10.1039/c4cp05191b)
37 Zaitsau, D. H., Fumino, K., Emel'yanenko, V. N., Yermalayeu, A. V., Ludwig, R., Verevkin, S. P. 2012 Structure-Property Relationships in Ionic Liquids: A Study of the Anion Dependence in Vaporization Enthalpies of Imidazolium-Based Ionic Liquids. *ChemPhysChem*. **13**, 1868-1876. (10.1002/cphc.201100879)
38 Deyko, A., Hessey, S. G., Licence, P., Chernikova, E. A., Krasovskiy, V. G., Kustov, L. M., Jones, R. G. 2012 The enthalpies of vaporisation of ionic liquids: new measurements and predictions. *Phys. Chem. Chem. Phys.* **14**, 3181-3193. (10.1039/c2cp23705a)
39 Zaitsau, D. H., Yermalayeu, A. V., Emel'yanenko, V. N., Schulz, A., Verevkin, S. P. 2017 Thermochemistry of Pyridinium Based Ionic Liquids with Tetrafluoroborate Anion. *Z. Anorg. Allg. Chem.* **643**, 87-92. (10.1002/zaac.201600335)
40 Zaitsau, D. H., Yermalayeu, A. V., Emel'yanenko, V. N., Butler, S., Schubert, T., Verevkin, S. P. 2016 Thermodynamics of Imidazolium-Based Ionic Liquids Containing PF6 Anions. *J. Phys. Chem. B*. **120**, 7949-7957. (10.1021/acs.jpcb.6b06081)
41 Yermalayeu, A. V., Zaitsau, D. H., Emel'yanenko, V. N., Verevkin, S. P. 2015 Thermochemistry of Ammonium Based Ionic Liquids: Thiocyanates-Experiments and Computations. *J. Solut. Chem.* **44**, 754-768. (10.1007/s10953-015-0316-2)
42 Emel'yanenko, V. N., Verevkin, S. P., Heintz, A., Corfield, J. A., Deyko, A., Lovelock, K. R. J., Licence, P., Jones, R. G. 2008 Pyrrolidinium-based ionic liquids. 1-butyl-1-methyl pyrrolidinium dicyanoamide: Thermochemical measurement, mass spectrometry, and ab initio calculations. *J. Phys. Chem. B*. **112**, 11734-11742. (10.1021/jp803238t)
43 Emel'yanenko, V. N., Zaitsau, D. H., Verevkin, S. P., Heintz, A., Voss, K., Schulz, A. 2011 Vaporization and Formation Enthalpies of 1-Alkyl-3-methylimidazolium Tricyanomethanides. *J. Phys. Chem. B*. **115**, 11712-11717. (10.1021/jp207335m)
44 Lovelock, K. R. J., Armstrong, J. P., Licence, P., Jones, R. G. 2014 Vaporisation and thermal decomposition of dialkylimidazolium halide ion ionic liquids. *Phys. Chem. Chem. Phys.* **16**, 1339-1353. (10.1039/c3cp52950a)
45 Marcus, Y. 1998 *The properties of solvents*. Chichester: Wiley.
